# Scale-Up to Pilot of a Non-Axenic Culture of Thraustochytrids Using Digestate from Methanization as Nitrogen Source

**DOI:** 10.3390/md20080499

**Published:** 2022-08-02

**Authors:** Denis de la Broise, Mariana Ventura, Luc Chauchat, Maurean Guerreiro, Teo Michez, Thibaud Vinet, Nicolas Gautron, Fabienne Le Grand, Antoine Bideau, Nelly Le Goïc, Adeline Bidault, Christophe Lambert, Philippe Soudant

**Affiliations:** 1 CNRS, IRD, Ifremer, Université de Bretagne Occidentale, UMR6539 LEMAR, F-29280 Plouzané, France; mariana.ventura@univ-brest.fr (M.V.); luc.chauchat@univ-brest.fr (L.C.); guerreiro.maurean@outlook.com (M.G.); michez.teo@gmail.com (T.M.); nicolas.gautron.37@gmail.com (N.G.); fabienne.legrand@univ-brest.fr (F.L.G.); bideau.antoine56@gmail.com (A.B.); nelly.legoic@univ-brest.fr (N.L.G.); adeline.bidault@univ-brest.fr (A.B.); christophe.lambert@univ-brest.fr (C.L.); 2 DENITRAL, F-22400 Lamballe, France; thibaud.vinet@orange.fr

**Keywords:** Thraustochytrids, *Aurantiochytrium*, PUFA, DHA, digestate, oxygen, scale-up, pilot

## Abstract

The production of non-fish based docosahexaenoic acid (DHA) for feed and food has become a critical need in our global context of over-fishing. The industrial-scale production of DHA–rich Thraustochytrids could be an alternative, if costs turned out to be competitive. In order to reduce production costs, this study addresses the feasibility of the non-axenic (non-sterile) cultivation of *Aurantiochytrium mangrovei* on industrial substrates (as nitrogen and mineral sources and glucose syrup as carbon and energy sources), and its scale-up from laboratory (250 mL) to 500 L cultures. Pilot-scale reactors were airlift cylinders. Batch and fed-batch cultures were tested. Cultures over 38 to 62 h achieved a dry cell weight productivity of 3.3 to 5.5 g.L^−1^.day^−1^, and a substrate to biomass yield of up to 0.3. DHA productivity ranged from 10 to 0.18 mg.L^−1^.day^−1^. Biomass productivity appears linearly related to oxygen transfer rate. Bacterial contamination of cultures was low enough to avoid impacts on fatty acid composition of the biomass. A specific work on microbial risks assessment (in supplementary files) showed that the biomass can be securely used as feed. However, to date, there is a law void in EU legislation regarding the recycling of nitrogen from digestate from animal waste for microalgae biomass and its usage in animal feed. Overall, the proposed process appears similar to the industrial yeast production process (non-axenic heterotrophic process, dissolved oxygen supply limiting growth, similar cell size). Such similarity could help in further industrial developments.

## 1. Introduction

Oceans are today exposed to dramatic pressure on fishing resources. The unsustainability of marine fish catches rose from 10–15% in the 1970s to 35% in 2017 [1]. From the approximately 85 million tons of marine fishes caught in 2018, 18 million were used for fish meal and fish oil production, mainly in aquaculture and breeding.

Fish oil is part of a large panel of the aquaculture diet, as it supplies long-chain n-3 polyunsaturated fatty acids (LC n-3 PUFA), a prerequisite for fish growth and for the nutritional quality of the final products [2]. 

Docosahexaenoic acid (DHA) is the most abundant LC n-3 PUFA in the mammalian central and peripheral nervous system and plays a crucial role in brain and retina development, cognitive function and visual acuity [3]. DHA is involved in pre- and postnatal development and is particularly important in the later stages of pregnancy and early infancy. Recent studies have also demonstrated that food intake of DHA has a beneficial effect on a number of psychological, behavioral, and neural disorders, such as Alzheimer’s disease [4]. DHA might have played a critical role in human evolution, as DHA is a determinant of neuronal migration, neurogenesis and the expression of several genes involved in brain growth and function [5]. Although human beings can synthesize DHA from n-3 PUFA precursors, it is more efficient to have a dietary supply of DHA to meet our health requirements [2]. This high demand results in a clear risk that DHA supply, mostly from small pelagic fishes, may become insufficient for a growing human population by 2040 [6].

As the supply of PUFA from fish catches is becoming a hurdle [7], alternative sources are being investigated, and among them, research has focused on Thraustochytrids [8], planktonic microorganisms that exhibit high DHA content [9,10] and has led to recent industrial application [11]. Obviously, as annual fish oil production in the world accounts for as high as 1 million tons per year [1], the Thraustochytrids production could be a significant and realistic alternative only if developed up to a large industrial scale and at a competitive market price. Then, production cost at industrial scale for DHA-containing biomass would be equal or lower than the current market price for an equivalent DHA supply in products obtained from small pelagic fisheries.

For this to happen, the Thraustochytrids industrial production process should fulfill the requirements for both high productivity and low production cost, as observed for example, in the yeast industry.

One of the usual means to reduce microorganism production cost is, when possible, to run the microbial cultivation under non-axenic (or “non-sterile”) conditions, as it leads to significant reductions in both investment and running costs. Such conditions were pointed out as key to competitive process development in “next generation biotechnology” [12]. When this approach is usual for large-scale phototrophic cultivations, its application to heterotrophic cultures can be more difficult, as massive bacterial contamination can rapidly appear in organic nutrient-rich culture media. However, this can be solved by establishing a competitive advantage for the cultivated strain. A competitive advantage can be obtained by means of high inoculation rate and short-term culture as applied for yeast production [13], by harsh environmental conditions (i.e., pH, temperature, salinity) that the cultivated strain can competitively withstand [14], or even by strain engineering for its growth on a specific substrate that competitors cannot use [15]. High inoculation rates and short-term culture could be applied to Thraustochytrids. 

Another approach to reduce production cost can be the use of fed-batch or continuous cultures [16,17], as they allow one to increase the productivity and thus reduce investment and process costs.

Substrates for culture medium often strongly affect the biomass production cost. As Thraustochytrids exhibit a heterotrophic metabolism, the organic substrates, sources of carbon and energy, are a key point in the final production cost. Therefore, they must be cheap and allow a high substrate-to-product conversion yield. Low-cost by-products from agriculture and from the food industry are available in large amounts and were investigated for Thraustochytrids production [18,19]. These include by-products from the food industry (whey and derivatives, plant process by-products) and from the anaerobic digestion (AD) process (digestates). They could be used in non-sterile microbial processes [12,20]. Using such by-products also contributes to the development of a circular economy as part of sustainability. The digestate is an emerging by-product, produced in large amounts in northwestern Europe. Its disposal as agricultural fertilizer leads, when in excess, to nitrogen and phosphorus pollution, resulting in eutrophication of natural waters. 

As partner in the INTERREG North West Europe ALG-AD project, we investigated the scale-up of Thraustochytrids production using AD digestate as a source of nitrogen and minerals. As recycling animal waste is a sensitive issue in Europe, a regulatory review on the use of digestate to cultivate algal biomass for animal feed was realized within the framework of the ALG-AD project (see report in supplementary files). With the aim of industrial scalability, we focused on economically sound process development by complying with the above-described goals and constraints. The objective was thus to assess the feasibility of the biomass production process at pilot scale in non-axenic conditions, and to achieve high productivity and cell density on AD digestate and corn syrup hydrolysate as main substrates, the ultimate objective being the industrial production of biomass for DHA and protein supplementation in feed. We describe in this paper the successive steps, up to a non-axenic process at 500 L scale. We present the identified key points of the process and the productivity and conversion yield observed. 

## 2. Results

### 2.1. Cultures Submitted to Air Bubbling and Shaking Exhibit Linear Growth

**Figure 1:**
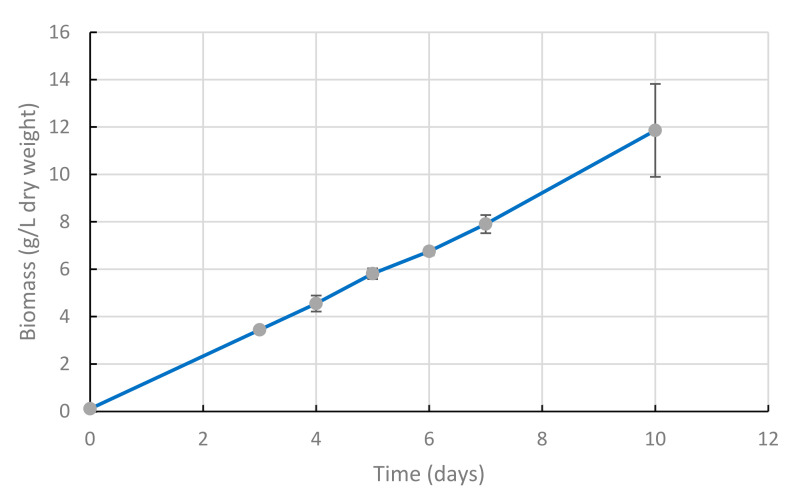
Biomass production kinetics of *A. mangrovei* cultivation in a modified YEP medium, with air bubbling 0.4 VVM. Volume of culture was 8 L before day 3, and 10 L from day 3 to day 10, due to addition of 2 L of a 500 g.L^−1^ glucose solution on day 3. Three replicates, error bars represent S.D.

The first scale-up step was to run 8 L cultures in a modified yeast extract and peptone (YEP) medium with continuous air supply (Cf. Section 4.4.1). The specific growth rate (Figure 1) exhibited an unusual linear shape, as compared to common microbial exponential growth rates. In usual exponential growth conditions, the biomass production per time unit increase as the cell density increases, usually expressed as:

X = X° × Exp µ × (t − t°), where X° is the biomass at time t°, X is the biomass at time t and µ is the growth rate.

In this experiment, we observed a different, stable biomass production rate over time, which can be expressed as:

X = X° + A × (t − t°). where productivity is A = 0.05 g.L^−1^.h^−1^.

In exponential growth, as described by Monod [21] the growth rate is determined by the limiting nutrient concentration. In this experiment, glucose was in large excess (16 g/L total added), and initial proteins from yeast extract (Y.E.) and peptones (approximately 3.5 g/L) represented 50% of the 7 g/L biomass dry cell weight (DCW) measured after 6 h of cultivation. This implies that the limiting factor for the growth rate was likely not related to nutrients. The constant biomass production per time unit suggests that the limitation is related to the constant and limiting supply of an external component. Preliminary experiments showed that growth remained poor when bubbling was lacking and dissolved oxygen values were below 10% after 2 days in bubbling cultures. This critical role of oxygen supply in Thraustochytrids cultures was already reported [11]. The volumetric oxygen mass transfer coefficient can be described as: 

dCl/dt = Kl.a × (C* − Cl) where dCl/dt is the oxygen transfer rate, Kl.a is the volumetric oxygen mass transfer coefficient, C* is the dissolved oxygen value when medium is saturated, and Cl is dissolved oxygen value at time t [22]. Thus, in this experiment, the volumetric oxygen mass transfer coefficient Kl.a that drove oxygen transfer at constant rate, as dissolved oxygen was low and stable, was likely the limiting factor that drove the linear biomass production rate. 

Then, the productivity can be expressed as a linear function of the oxygen transfer Kl.a, and the biomass production rate can be expressed as: 

X = X° + α × Kl.a × (t − t°)

where α is a strain related constant value when the substrate is not limiting. 

This model points out the oxygen transfer rate as the factor that drives the productivity in such cultures, when nutrients are not limiting. Therefore, during the further scale-up steps, special attention was dedicated to oxygen transfer efficiency. Further experiments should be run to confirm this proposed model. 

### 2.2. Comparison of Performance of Cultures in Digestates from Three Different Sources

**Figure 2:**
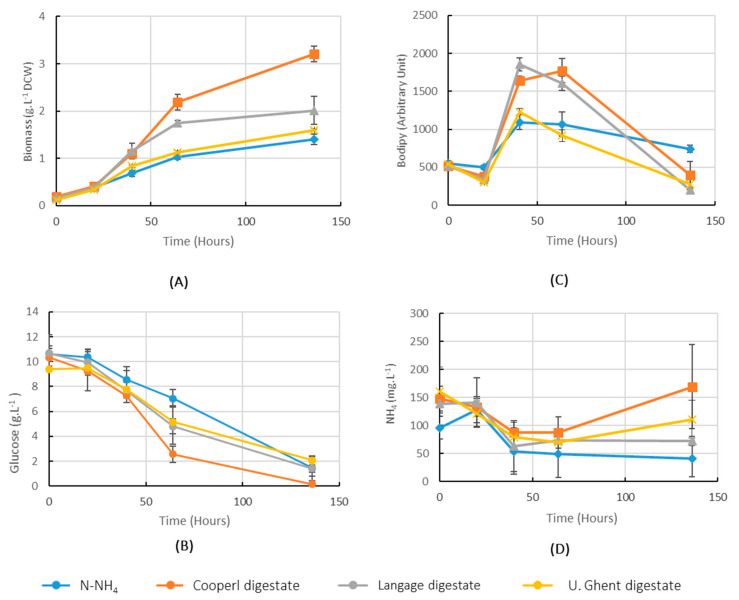
Cultures in bubbling and shaking 800 mL supplemented with digestates from three different sources and with 0.297 g.L^−1^ NH_4_Cl (final concentration) as control source of nitrogen (see Section 4.1.2 for detailed description of culture medium). Mean values (*n* = 3, error bars = S.D.). (**A**): biomass production kinetics; (**B**): glucose concentration in cultures; (**C**): relative lipid content in cells as measured by BODIPY-stained cell fluorescence; (**D**): NH_4_ concentrations in cultures.

The performance of digestates, made from three different anaerobic digestion biomass sources, was compared over 136 h in 800 mL of aerated and agitated cultures in flasks. Based on previous NH_4_ content analysis, the digestate volumes added in cultures were adjusted in order to start the culture with 100 mg.L^−1^ of NH_4_ in each culture. The added volume of NH_4_Cl solution was calculated to obtain 100 mg.L^−1^ in control culture (Cf. Section 4.1.2). Biomass production (Figure 2A) and glucose consumption (Figure 2B) showed the Cooperl (pig manure) digestate gave the highest biomass (3.2 g.L^−1^ DCW), while Langage digestate (agricultural and food by-products) and plant-based digestate (U. Ghent) allowed 2 g/L and 1.6 g/L, respectively. However, all digestates allowed a biomass production higher than the NH_4_Cl control (1.5 g.L^−1^ DCW) (as digestates were filtered at 0.2 µm, their contribution to biomass was negligible). This suggests that digestates did not only supply N-NH_4_, but also other growth-promoting nutrients. Lipid content, as illustrated by fluorescence level of BODIPY-stained cells (Figure 2C), dropped at the end of culture. Together with the increase in N-NH_4_ values (Figure 2D), these drops could be due to cell stress as other nutrient availability dropped (Figure 2B). The following experiments were all performed with Cooperl digestate.

### 2.3. The Digestate Concentration Influences the Growth Kinetics in Flask Cultures

**Figure 3:**
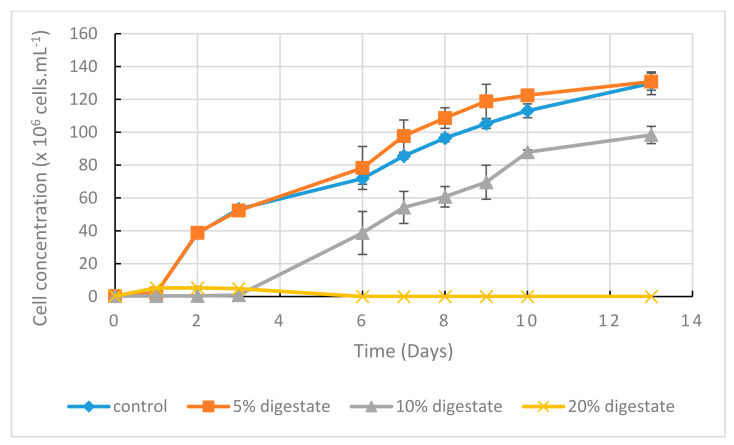
Biomass growth kinetics of *A. mangrovei* in 150 mL cultures in 500 mL shaken flasks. Digestate as % *v*/*v*. Mean values (*n* = 3, error bars = S.D.). Cell concentrations were estimated using O.D. measurement at 620 nm. Relationship between cell concentration and O.D. at 620 nm was previously established by the following equation: cell concentration = 1 × 10^8^ × (O.D. at 620 nm) − 1 × 10^7^ (*R*^2^ = 0.98).

The influence of digestate supply quantity was investigated in flask cultures (Figure 3). The addition of 5% digestate allowed, as compared to YEP modified control condition, a slightly higher cell concentration between 7 and 10 days, and the same final cell concentration after 13 days.

Higher digestate concentrations induced a reduction in cell concentration (10% digestate), or worse, inhibited growth (20% digestate). We concluded that digestate exhibited a positive effect only for the lowest percentage tested (5%), while a toxic effect seemed to appear for higher percentages. This is possibly due to the toxicity of ammonium in solution [23], and/or possibly to other unidentified deterrent compounds. We then decided to run the following experiments with 2.5% digestate in batch cultures and up to 5% in fed-batch cultures.

### 2.4. Batch Cultures Can Be Run in Non-Axenic Airlift Cylinders 

In order to study the scalability of the culture on digestate and in non-axenic conditions, cultures were run in 10 L PMMA cylinders. Two options were tested for air supply: large bubbles (4.5 mm diameter bottom tube) similar to carboy cultivation, and smaller bubbles (five holes, 1.5 mm diameter, in a bottom PVC plate). Three flow rates (0.8, 0.4 and 0.2 VVM) were tested with the small bubbles. As an axenic control, a cultivation was run in an agitated carboy with the large bubbles for aeration. These cultures were preliminary tests prior to pilot scale-up and were not replicated. 

**Figure 4:**
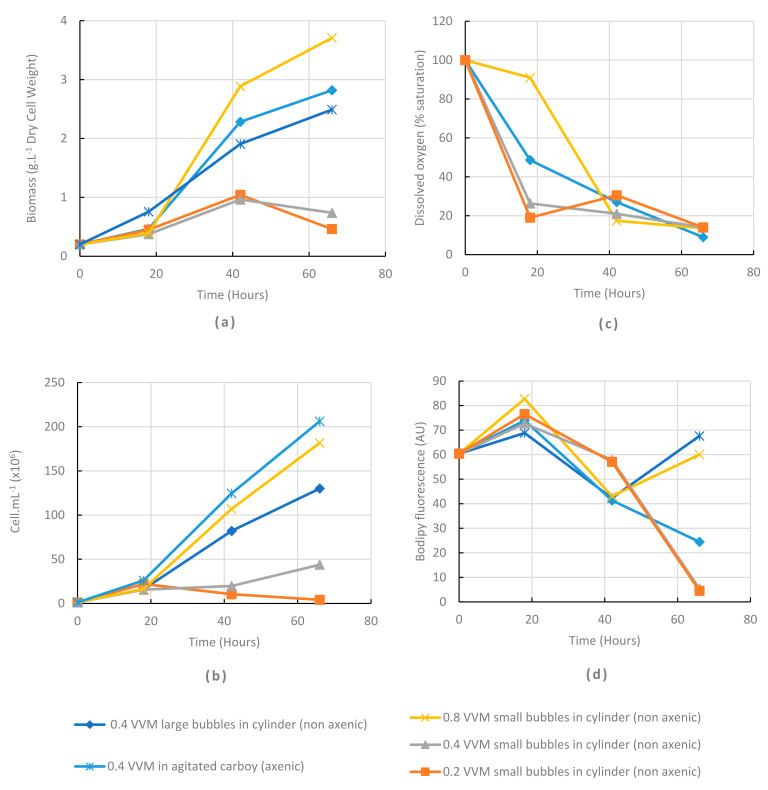
Effect of air bubbling systems on cultures (6 L) in non-axenic airlift cylinders and in an axenic carboy with air bubbling and 100 rpm shaking as control. (**a**): biomass production kinetics; (**b**): Cell concentration kinetics; (**c**): Dissolved oxygen concentration; (**d**): relative lipid content in cells as measured by BODIPY stained cell fluorescence.

Results (Figure 4a) showed that the highest biomass concentration value (3.7 g.L^−1^) in non-axenic cylinder was obtained after 66 h of culture, with small bubbles at the maximum tested flow rate (0.8 VVM). Both cylinders with small bubbles at lower flow rates (0.4 and 0.2 VVM) allowed only poor cell and biomass production (Figure 4a,b). As the dissolved oxygen values were also the lowest in these two cylinders (Figure 4c), oxygen transfer limitation likely explained this lower growth performance. At 0.4 VVM, large bubbles, as compared to small bubbles, allowed higher dissolved oxygen values, with higher cell growth and biomass values. We hypothesized that the airlift effect was less efficient when small bubbles were used, then reducing oxygen transfer and mixing. 

Intracellular lipid content was measured by flow cytometry (green fluorescence level in arbitrary unit after staining with BODIPY [20]). The BODIPY values were the highest (Figure 4d) at 66 h in the three cultures that exhibited the highest cell density, and were similar in axenic and non-axenic cultures. This suggests that cell size and/or cell lipid content were increased in these highly aerated cultures and that the potential bacterial presence did not dramatically modify the kinetics as compared to axenic cultures. Bacterial presence, as observed by microscopy (with a 40× objective and 10× eyepiece), was detected but low (few bacteria in the field) in all cylinders. Because we were using a large culture volume (6 L), the experiment was performed without replicates. Therefore, it should only be considered as indicative. Nevertheless, it showed that non-axenic cultivation appeared feasible and permitted running further tests at pilot scale.

### 2.5. Non-Axenic Cultivation in Pilot-Scale (500 L) Culture in Batch and Fed-Batch Modes Appears Feasible

**Figure 5:**
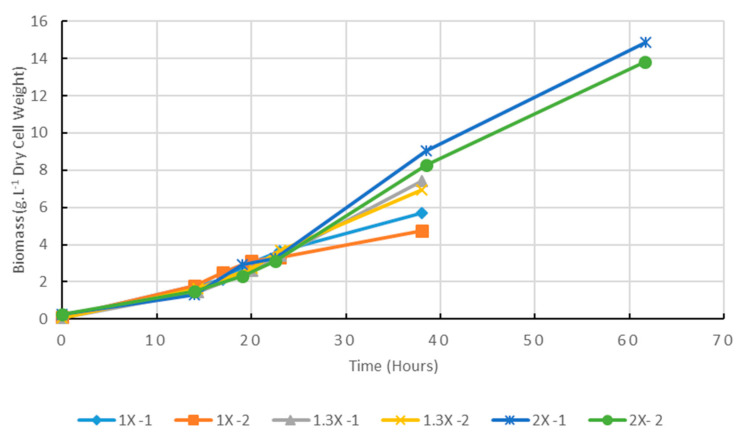
Effect of medium supplementation on biomass production in 500 L reactor cultures (2 replicates). 1X-1, 1X-2: simple batch (no feeding); 1.3X-1, 1.3X-2: feeding with 30% of initial medium organic concentrate after 16 h; 2X-1, 2X-2: feeding with 100% of initial medium organic concentrate after 16 h.

Cultures were performed in 800 L cylinders, in 500 L culture medium. Three culturing modes were tested: (1) simple batch; (2) batch with feeding after 16 h of cultivation with 30% of the initial media nutrients (glucose, peptone, yeast extract and digestate); or (3) batch with feeding after 16 h with 100% of initial media nutrients. The cultures were harvested after 38 h for (1) and (2) and 62 h for (3). Before 16 h, growth rates appeared similar (Figure 5). Dissolved oxygen measurement showed, in fed cultures after 24 h, values ranging from 2 to 9% of saturation (range values obtained from 11 simple batches; kinetics of dissolved oxygen values during 24 h of 11 simple batches are presented in Figure S1, supplementary material). Stability of dissolved oxygen level after 9 h of culture revealed that the oxygen transfer rate was equal to the oxygen uptake rate. On the other hand, as aeration rate does not change, and as the physical properties of the liquid medium, and its volume can be considered as stable, then the oxygen transfer rate does not change. These stable oxygen transfer rate and uptake rate could induce a linear growth, as already discussed above (Cf. Section 2.1).

However, as the number of samples collected over time was too scarce, data only allowed suggesting that a similar linear growth would occur in this pilot experiment after 15–20 h. Productivity and yields are discussed below (Cf. Section 2.9).

### 2.6. Bacterial Contamination Assessment in Non-Axenic Cultures

Bacterial contaminations could be critical for the quality and the security of use of biomass from non-axenic cultures. A series of 14 cultures at pilot scale were performed from July 2020 to January 2021. Among these cultures, temperatures ranged from 26 to 30 °C at 0 h and from 27 to 34 °C at 24 h (Figure S2, supplementary material), pH ranged from 7.1 to 7.8 at 0 h and 3.8 to 6.3 at 24 h (Figure S3, supplementary material), culture volumes were 450L or 500L, mean inoculum concentration was 2.9 × 10^6^ ± 5.5 × 10^5^ cells.mL^−1^ (min = 2.1 × 10^6^ and max = 3.9 × 10^6^), and culture harvest was performed after 24 to 62 h of cultivation. The contaminations were first assessed during the process by microscopic observation of fresh cultures. The only detected contaminations were bacteria, which were always present. It must be noted that in some cultures, these contaminants were less present by the end of the culture. Some lab tests were run to study a putative antibacterial effect of culture supernatant, but were unsuccessful. We thus hypothesized a bacterivory impact of *A. mangrovei,* as such a function was previously reported in Thraustochytrids [24]. In order to allow the use of the biomass for feed trials, a microbial risk assessment was performed by ANSES (the food and environmental safety agency of France) on a September production run (and on a previous production run), within the framework of the ALG-AD program. The report established that products were safe for use as feed (Cf. Report Interreg NWE ALG-AD project on Safety analysis digestate and algal biomass produced by the three ALG-AD pilots in Supplementary Files). 

**Figure 6:**
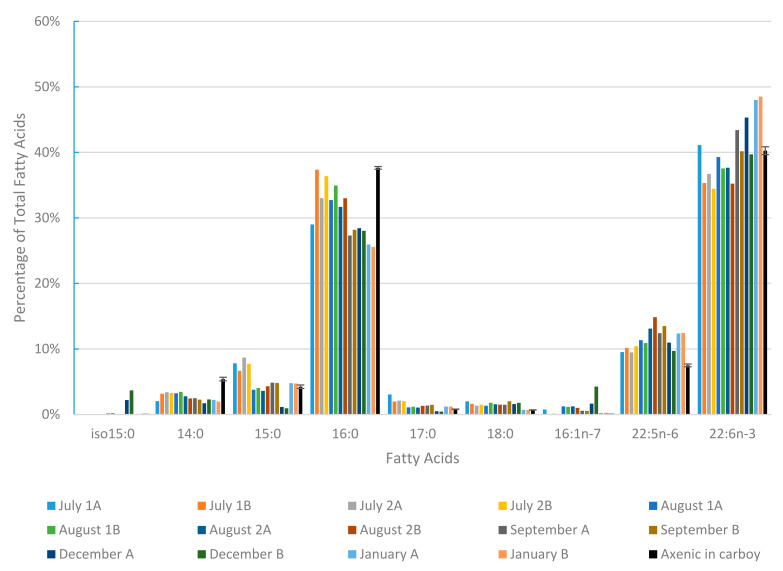
Relative distribution of the main fatty acids from 14 cultures of 450–500 L (black bars are an average of 3 axenic cultures in carboys on modified YEP medium. Error bars = S.D). January A and January B are the fed batch cultures (+30% of the initial media; see Section 2.5 for details).

In order to further estimate bacterial contamination, a detailed analysis of fatty acids distribution was performed for each culture endpoint sample. A focus was placed on iso 15:0, known as a specific proxy of marine bacterial contamination [25] in favorable growth conditions [26]. The fatty acid composition of an axenic culture (Cf. Section 2.1) was also measured and used as a reference. Results (Figure 6) showed that iso 15:0 was detected at a level below 0.04% in the axenic control. Two cultures exhibited values of 0.15–0.20%, and two cultures showed contamination in the range of 2–4% (December A and B). These cultures concomitantly exhibited an increase in 16:1n-7, suggesting that this fatty acid could be also a proxy for bacterial contamination in our culture conditions. However, even in the most contaminated cultures, the percentage of the main *Aurantiochytrium mangrovei* fatty acids remained within the range of the other cultures and axenic control. Only 15:0 and 17:0, generally present at low concentrations, were reduced when bacterial fatty acids were at their highest. 

Such results illustrate that this non-axenic process can be performed at pilot scale without significant impact of bacterial contamination on the lipid characteristics of the biomass. 

### 2.7. Glucose Conversion and Nitrogen Trapping Efficiency 

**Figure 7:**
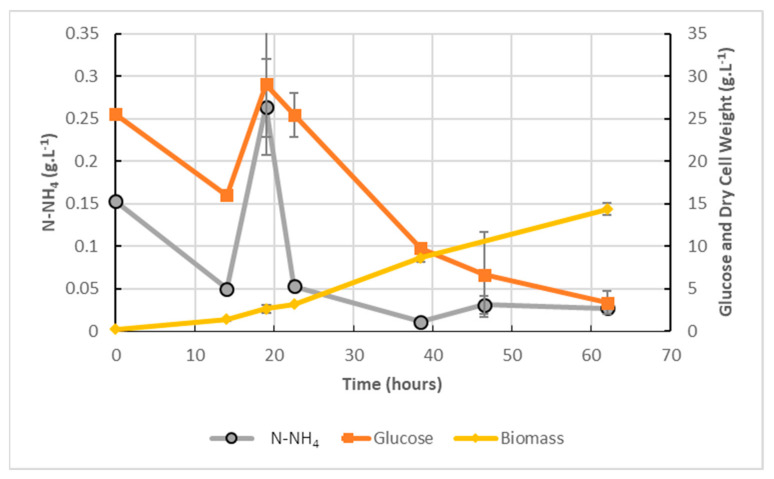
Biomass (DCW g.L^−1^), glucose (g.L^−1^) and nitrogen (g.L^−1^) concentrations in 500L airlift fed-batch culture; 100% of initial organic substrate was added at 16 h. Mean (*n* = 2, error bars = S.D.).

The most productive pilot-scale culture (fed-batch 2X, 5% digestate, 40 g.L^−1^ glucose) was assessed for glucose conversion and for N-NH_4_ depuration efficiency. Figure 7 shows that from 20 g.L^−1^ of glucose added at 0 h followed by a second addition of 20 g.L^−1^ at 20 h, only 3.3 g.L^−1^ remained at the end of culture, and that it was reduced progressively as biomass concentration increased. From the 0.30 g.L^−1^ N-NH_4_ added in medium, only 0.025 g.L^−1^ remained at the end of the culture (more than 90% depuration ratio). Ammonia stripping due to aeration could occur in cultures when pH values are above 7 [27]. However, as pH was maintained below pH 6.5 after 14 h (data not shown), the rapid drop in N-NH_4_ observed after feeding (16 h) could be assigned to cell trapping of N-NH_4_, illustrating the high efficiency of *A. mangrovei* in depuration of N-NH_4_ and the use of NH_4_ from digestate as part of the nitrogen source in medium. 

### 2.8. Lipids and Proteins in Pilot-Scale Production 

**Figure 8:**
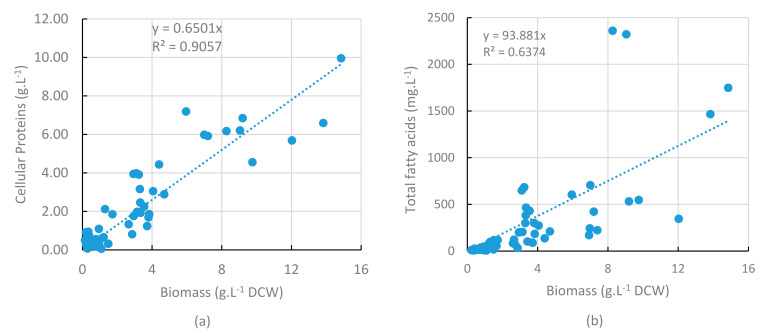
Cellular protein (**a**) and total fatty acid (**b**) concentrations versus biomass concentration, from a data set of 14 kinetics in 450 and 500 L cultures and 3 kinetics in 260 L cultures, over a 6 month period.

**Figure 9:**
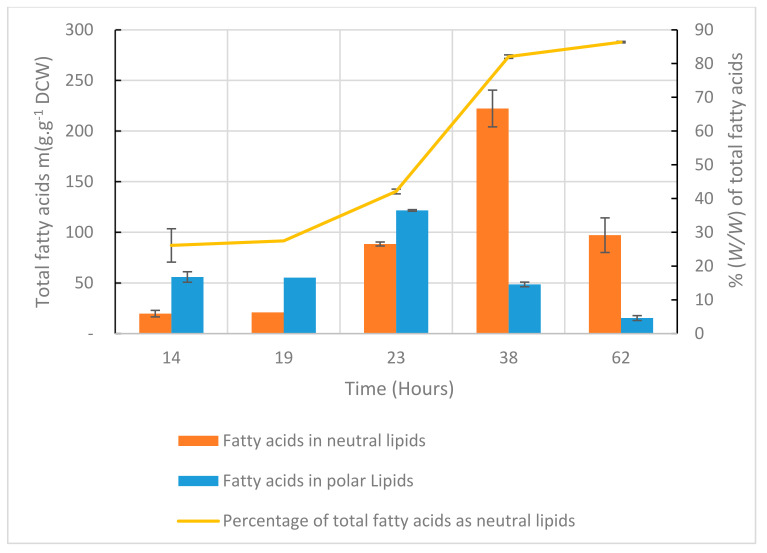
Mean concentration (*n* = 2, error bars = S.D.) over a 2X-fed-batch kinetic in a 500 L culture, of neutral and polar fatty acids in biomass, and percentage (*w*/*w*) of neutral lipids over total lipids.

In order to assess the characteristics of the biomass produced, analyses were run on a set of 280, 450 and 500 L pilot cultures, with samples collected over culture time. Figure 8a shows a correlation between the cell protein concentration and the DCW concentration in samples. Then, in spite of an important dispersion of values over the curve, it seems that the proportion of protein in cells remained stable in cultures. In contrast, the lipid concentration in samples appeared highly variable and could not confidently be related to DCW concentration (Figure 8b, *R*^2^= 0.51). This variability could be explained by a strong qualitative and quantitative change in lipids over the culture, as illustrated from the 2X fed-batch culture (Figure 9): both polar and neutral lipids increased (as mg.g^−1^ DCW) during the first phase of the culture. After 23 h for polar lipids and 38 h for neutral lipids, these values started to reduce when the neutral lipids/polar lipids ratio increased. This could be explained by a shift in the lipid synthesis process from membrane lipids (polar lipids) produced during the early phase to mostly reserve lipids (neutral lipids) synthesized during the late phase. The decrease in neutral lipids by the end of the culture could be due to the catabolism of this energy reserve when glucose was almost fully depleted. 

### 2.9. Productivity and Conversion Yields of Substrate to Biomass 

In Table 1, we compared the productivities from various experiments. The lowest productivities were observed when no air bubbling was applied. Highest productivities were observed in 500 L cultures. They were 3 times higher or more, as compared to productivities in 10 L cylinders. This resulted from higher cell densities obtained in a shorter time. These large cylinders allow a high water column (1 m), and their large diameter and center-focused bubbling allow an efficient airlift effect and, therefore, a high mixing and oxygen transfer efficiency. Productivities were further increased when fed batch mode was applied, both in axenic and non-axenic cultures, and with full substrate or glucose-only addition. This could be explained both by a longer part of the culture spent at maximum linear cell production rate (the less productive initial phase of the culture represented a shorter part of the whole culture time), and by a possible positive influence of the high concentration of organic substrates (Y.E and peptone and/or glucose) and/or digestate on this maximum cell production rate. Such positive effect of feeding on cultures was already reported [11,16,17]. 

Yields were calculated as DCW produced per initial organic substrate added in medium. Then, when substrates were remaining in media, the substrate to biomass conversion efficiencies were underestimated. Despite this, a yield (Table 1) of 0.30 was observed in 2X fed-batch 500 L culture, 0.32 was observed in glucose and salts medium, and 0.28 in YEP-modified medium. The two last yields were obtained when productivities were low. This suggests that the energy budget for long-term cell maintenance, needed when productivity is low, does not influence the yield. With such yields, 3.3 kg of organic substrate is needed to produce 1 kg of DCW Thraustochytrids. 

## 3. Discussion

### 3.1. Non-Axenic Cultivation

In our goal to develop an economically realistic industrial process for Thraustochytrids production, a key point was to succeed in running cultivation under non-axenic conditions. To our knowledge, such an approach has not been investigated to date. Microbial sanitary risks evaluation for feed use of our product did not point out any sanitary risks and confirmed the possibility of using this biomass as feed. Furthermore, lipid quality assessment over a 6 month survey of productions (Figure 6) showed that even when bacterial contamination was detected, the fatty acid profile was not significantly impacted. More details on seasonal variations in production can be found in the supplementary file “Interreg NWE ALG-AD project Seasonal variation of algal biomass cultivated using nutrient rich digestate”. Together with positive feed trials on this product [28], these results allow considering further investigations of performance and scale-up of non-axenic industrial production of Thraustochytrids. 

### 3.2. Substrates 

The use of by-products in order to reduce the costs of process and the environmental impacts of the agro-industry is a major concern. We have shown here that digestate can be used in non-axenic conditions, at pilot scale, in fed-batch mode. The fed-batch mode was already successfully investigated in Thraustochytrids studies [16]. We have shown here that, to avoid a negative impact on growth, digestate from anaerobic digestion (AD) should be added only in small amounts (Figure 3). However, the addition of higher amounts under progressive fed-batch mode should be investigated, as digestate could be progressively metabolized and toxicity avoided. Ultimately, it will allow reducing the usage of yeast extract and peptone to culture medium.

### 3.3. Limiting Factor for Growth and Productivity

We showed (Figure 1 and Figure 4) that the controlling factor for growth was the oxygen availability and that biomass productivity increased when culture volume increased (Table 1). This could be explained by an increase in the oxygen transfer coefficient (Kl.a) when increasing the bioreactor size. We can hypothesize that a further increase in reactor size (and height) could similarly allow a further increase in oxygen transfer, and then in productivity.

The maximum productivity (5.6 g.L^−1^.day^−1^), and maximum biomass concentration (14.3 g.L^−1^) observed in our 500 L fed-batch culture can be compared to reported values in the literature. We calculated, from a recent review from Du et al. [11], Thraustochytrids productivities ranging from 2.5 to 38 g.L^−1^.day^−1^ with biomass concentration from 4.8 to 152 g.L^−1^. It is difficult to comment on such data, as each value is related to a specific process. Furthermore, such values are only a part of the economic assessment. Productivities in our process were drastically increased from lab to pilot scale. Further scale-up should be investigated and could lead to further productivity improvements. 

This process shows similarities with the yeast production processes [13,29]. They include non-axenic last step(s) after axenic cultivation, fed-batch mode, oxygen as major limiting factor for growth and high substrate to biomass conversion yield. 

The yeast production industry uses low-cost substrates, whereas our process still used small amounts of costly peptone and yeast extract. However, we showed at lab scale (Figure 2 and Table 1) that such compounds are avoidable when maintaining yield.

### 3.4. Lipids

This work was focused on biomass production and process feasibility, and no specific attempt was made to increase lipids or DHA content. DHA productivity ranged from 0.10 to 0.18 mg.L^−1^.day^−1^. Fatty acids content in our cultures (i.e., 280 mg.g^−1^ DCW at maximum and 110 mg.g^−1^ DCW at harvest, in 2X fed-batch, Figure 9) was far below reported maximum values obtained when, for example, the C/N ratio or dissolved oxygen profiles over the culture were specifically designed [11]. However, harvested biomass from pilot productions was tested in feed trials [28] and proved suitable for fish feed. 

In order to further improve productivity and yield together with DHA content, further investigations should include tests on the C/N ratio over the culture phases, eventually coupled with air supply modification during late phase [30,31,32] and further increases in feeding rate. 

### 3.5. AD Digestate Regulation

The use of AD digestate as nitrogen and mineral substrate in industrial cultivation would be an opportunity to reduce adverse impacts such as eutrophication related to excessive agricultural spreading. Unfortunately, recent investigations about the applicable European regulation on AD digestate showed that animal-based digestates could not be used for feed production (see the report of Interreg NWE ALG-AD project “A Regulatory Review on the Use of Digestate to Cultivate Algal Biomass for Animal Feed” included in supplementary file). This limits the potential industrial scale–up of this process to only crop-based digestates. However, other by-products from the food industry, such as milk derivatives and vegetable processing by-products, could be investigated as potential alternatives to digestate.

This paper showed that the use of a process of the same kind as the widely used low-cost yeast technology, together with the use of low-cost substrates and by-products, could lead to the development of a low-cost process for industrial-scale production of biomass from Thraustochytrids. Low-cost substrates and by-products include dairy waste water, spent brewery yeast, and spent osmotic solution from the candied fruit industry. This process still needs an optimization step for DHA production. For instance, in order to be competitive in the fish feed industry, the production cost of our Thraustochytrid biomass (in dry matter) should range between EUR 3 and 6 per kg according to the targeted fish species and stages. Then, such economically sound development could turn to reality the hope for a reduction in industrial fishing for feed and food.

## 4. Materials and Methods

### 4.1. Culture Media 

#### 4.1.1. Modified YEP Medium for Lab Experiments and Pilot Precultures

The YEP-modified culture medium was prepared as follows: Yeast extract (VWR^TM^ 84601.5000): 2 g; peptone from casein (VWR^TM^, Rosny-sous-bois, France) peptone from casein 84610.0500): 2 g; sea salt (Sigma^TM^, Saint Louis, MO, USA; Sigma S9883):15 g; dextrose (TITOL chimica SpA^TM^, Pontecchio Polesine, Italy, Glucose anhydre pur CA:50-99-7): 20 g; water: 1 L, autoclaved for 20 min at 120 °C. When added, the digestate obtained from anaerobic digestion of pig manure was stored for settling of large particles, adjusted to pH 7 by H_2_SO_4_ addition, and autoclaved for 20 min at 120 °C.

#### 4.1.2. Modified Guillard and Ryther F/2 Medium for Digestate Comparison Experiment

The medium [33] was designed to allow the supply of nitrogen either from digestate or from NH_4_Cl as control. A digestate produced by anaerobic digestion (AD) from agriculture and food by-products was obtained from the Langage AD site (Plymouth, Great Britain), an animal waste-free AD digestate was obtained from University of Ghent (Belgium), and a pig manure digestate was obtained from the Cooperl site (Lamballe, France).

- Digestates and NH_4_Cl (297 g.L^−1^) solution were filter-sterilized through 0.2 µm filter.
- The vitamin solution was prepared as follows: cyanocobalamin: 5 mg; biotin: 5 mg; thiamine HCl: 1 g; distilled water: 500 mL, autoclaved 20 min at 120 °C.
- The trace element solution was prepared as follows: stock solution: CuSO_4_.5H_2_O: 100 mg, ZnSO_4_·7H_2_O: 220 mg, CoCl_2_·6H_2_O: 100 mg, MnCl_2_·4H_2_O: 1800 mg and Na_2_MoO_4_·2H_2_O: 60 mg. Distilled water: 200 mL. The final trace element solution contained stock solution: 20 mL; Na_2_EDTA: 4.36 g; FeCl_3_·6H_2_O: 3.15 g; distilled water: 980 mL. Solution was filter-sterilized through 0.2 µm.
- Each 800 mL culture was prepared independently as follows:

Twelve grams sea salt (Le Saulnier de Camargue^TM^, Clichy, France) was added in a volume of distilled water calculated to obtain 800 mL of final volume, and autoclaved for 20 min at 120 °C. Solutions were then added in a sterile manner: Na_2_HPO_4_·7H_2_O (56.8 g.L^−1^, autoclaved for 20 min at 120 °C): 20 mL; MgSO_4_·7H2O (42 g.L^−1^, filtered through 0.2 µm): 8 mL; glucose (500 g.L^−1^ autoclaved for 20 min at 120 °C): 16 mL; trace elements solution: 0.8 mL; vitamin solution: 0.8 mL. The nitrogen source was then added: either NH_4_Cl (0.297 g.L^−1^, final concentration): 0.8 mL, Cooperl digestate: 25.8 mL, Langage digestate: 16.7 mL, or U. Ghent digestate: 15.1 mL; pH was finally adjusted to 6.5 in each flask by successive sterile sampling and acetic acid addition.

#### 4.1.3. Culture Media for Digestate Concentration Tests

Yeast and peptone concentrate (4 g.L^−1^ each) in sea water and glucose concentrate (200 g.L^−1^) in sea water were prepared and autoclaved for 20 min at 120 °C. Digestate (unfiltered) was autoclaved for 20 min at 120 °C. Chemical characteristics of digestate are provided in Table S1. Solutions were mixed in a 500 mL sterile flask and adjusted with sterile sea water in order to obtain 150 mL of a solution with: 2 g.L^−1^ peptone; 2 g.L^−1^ Y.E.; 60 g.L^−1^ glucose and either 0 (control), 5, 10 or 20% digestate (*v*/*v*). 

#### 4.1.4. Medium for Non-Axenic Culture in 10 L Cylinder Experiments at Lab

Media (6 liters each) for non-axenic cultures in 10 L cylinder and for axenic culture in 20 L carboy were prepared as follows:

Solution 1: Sea salts from Le Saulnier de Camargue^TM^ untreated: 90 g; digestate from Cooperl AD pilot plant, centrifuged in order to eliminate large particles and then filtered through 300 KDa tangential flow filtration membrane: 150 mL (2.5% *v*/*v*); tap water: 5.12 L. For carboy culture, the medium was split into five 2 L bottles and autoclaved for 20 min at 120 °C.

Solution 2: Glucose: 150 g; water: 60 mL. Autoclaved for 20 min at 120 °C.

Solution 3: Peptone: 12 g; yeast extract: 12 g; water: 600 mL. Autoclaved for 20 min at 120 °C.

Solutions 1, 2, and 3 were mixed in each cylinder or mixed sterilely in a previously autoclaved (20 min at 120 °C) carboy. Autoclaved (20 min at 120 °C) silicone-based anti-foam was added (final concentration: 1 mL.L^−1^): Antimousse 426R, Labogros^TM^ (Coueron, France).

#### 4.1.5. Medium for Non-Axenic 400 or 500 L Culture in 800 L Cylinders at Pilot Plant

The medium was prepared by addition of the following solutions:

Solution 1: Filtered digestate (2.5% *v*/*v*), containing 48.3 mg nitrogen/L, 2.6 mg phosphorus/L with an N/P ratio of 18.6. 

Solution 2: Glucose syrup Isosweet 470 (Tereos ^TM^, Moussy-le-vieux, France): 24 g.L^−1^ of culture medium (as 20 g.L^−1^ dextrose replacement). 

Solution 3: Yeast extract and peptone at 90 g.L^−1^ each, autoclaved for 20 min at 120 °C (for initial concentration in culture medium: yeast extract 2 g.L^-1^ and peptone 2 g/L).

Solution 4: Sea salt from Le Saulnier^TM^ (15 g.L^-1^ of culture medium); Mg SO_4_ 7H_2_O (0.46 g.L^−1^ of culture medium), tap water as needed. 

Silicone-based anti-foam was added as soon as the yeast extract and peptone solutions were poured into the cylinder (final concentration: 1 mL.L^−1^, Antimousse 426R, Labogros^TM^, Coueron, France). For fed-batch feeding, only digestate and organic substrate (Isosweet, peptone and yeast extract) concentrates were added as 30% of initial volumes for 1.3X, and 100% of initial volumes for 2X.

### 4.2. Thraustochytrid Strain

The thraustochytrid *A. mangrovei* was obtained from the Roscoff culture collection (Strain RCC 893). Cryovials for pre-culture were prepared as follows:

A 150 mL modified YEP culture was run in a 500 mL round flask on a shaking table at 100 rpm, 25 °C. One milliliter of culture at approximately 2 × 10^7^ cells/mL was centrifuged. The pellet was resuspended in 600 µL of fresh medium and transferred to a cryovial, and 600 µL of autoclaved (20 min at 120 °C) glycerol 40% in water was added. Vials were vortexed and, after 30 min at room temperature, were stored at −80°C.

### 4.3. Pre-Cultures 

#### 4.3.1. Pre-Cultures for Lab Experiments

Two 500 mL round flasks with 250 mL of modified YEP medium (Cf. Section 4.1.1) were inoculated with the content of a strain cryovial freshly thawed at room temperature. Cultures were maintained for 2 days at 23–25 °C on a shaking table (100 rpm). Two milliliters of this first culture were then used to inoculate four new flasks in the same conditions for 64 h. Flasks were used as inoculum for culture experiments.

#### 4.3.2. Pre-Cultures for Pilot-Scale Experiments

Eight liters of modified YEP medium (Cf. Section 4.1.1) were prepared, dispatched as approximately 1.5 L in 2 L bottles, and autoclaved for 20 min at 120 °C. The medium was then transferred in a previously autoclaved (120 °C, 20 min) 20 L carboy equipped with inlet and outlet 0.2 µm gas filters (Millipore ^TM^) and silicone tubing connected to brass end tubing (4.5 mm internal diameter). This brass tubing was in contact with the inside bottom of the carboy in order to allow bubbling in the medium. A sampling bottle (250 mL) was also connected to the silicone tubing in order to allow sterile sampling. 

A 250 mL amount of pre-culture (Cf. Section 4.3.1) was inoculated in the 8 L of modified YEP medium in a 20 L carboy. Cultures were run on a shaking table at 100 rpm, with air bubbling at 0.4 volume of air per volume of culture per min (VVM). After 20 h of cultivation, they were transported to the pilot site, maintained under bubbling. Inoculation of pilot cultures occurred within 6 h. 

### 4.4. Cultures at Laboratory Scale

#### 4.4.1. Scale-Up of Axenic 8 L Cultures in 20 L Carboys

Cultures were prepared following the same protocol as described above (Cf. Section 4.3.2). 

Three carboy replicates were prepared and maintained at 25 °C on a shaking table (100 rpm), and air was supplied at 2.4 L.min^−1^ (0.4 VVM). Samples for analysis were collected under a sterile laminar flow hood. After 3 days, 2 L of a 500 g.L^−1^ autoclaved (20 min at 120 °C) dextrose solution was added in each carboy (total glucose in final medium: 216 g.L^−1^).

#### 4.4.2. Comparison of Three Sources of Digestate 

Cultures in triplicate were run in 800 mL culture medium (Cf. Section 4.1.5), in 1 L flasks with a stainless steel, 2 mm ID tube dispensing 1VVM air through a 0.2 µm inlet filter and maintained on a shaking table at 90 rpm, 25 ± 1 °C. Three different sources of digestate and NH_4_Cl as control nitrogen source were tested. 

A cryovial (−80 °C) was inoculated in 250 mL medium in a 500 mL flask and run for 72 h, 90 rpm, 25 ± 1 °C. This culture (10 mL) was then inoculated in a new YEP-modified medium flask for 24 h. The resulting culture was used as inoculum for culture (40 mL in each 800 mL culture). Cultures were run in triplicate. 

#### 4.4.3. Effect of Digestate Concentration on Growth Kinetics in Flask Cultures

Cultures were prepared in 500 mL flasks with 150 mL culture medium (Cf. Section 4.1.2).

Cultures were run in triplicate at 16 ± 1 °C and 90 rpm on a shaking table. In this experiment, cell concentrations were estimated using O.D. measurement at 620 nm. The relationship between cell concentration and O.D. at 620 nm was previously established by the following equation: cell concentration =1 × 10^8^ × (O.D. at 620 nm) − 1 × 10^7^ (*R*^2^ = 0.98).

#### 4.4.4. Scale-up to Non-Axenic 6 L Cultures in 10 L Cylinders

Cultures (6 liters) were run in 10 L cylinders. Cylinders were home-designed, made of PMMA (100 mm ID) tubing closed with a flat PVC bottom. They were built by AFE consulting, France. The bottom was equipped with inlet tubing for sampling and for air bubbling. Two bubbling systems were tested: a 50 mm PVC plate with 5 holes (1.5 mm) and a 4.5 mm I.D. brass tube. Before each culture, the cylinders were filled up to the top with water, and two drops of 3.3 g of dichloroisocyanuric disodium salt at 81% (N° CAS: 5180-86-0; disinfectant bleach) were added. Cylinders were kept under bubbling for 24 h and then thoroughly rinsed with tap water. The silicone-based anti-foam was added (1 mL.L^−1^ of culture) immediately after inoculation in cylinders and in one of the carboys.

### 4.5. Cultures at Pilot Scale

The two cylinders were home designed, made of PMMA (800 mm ID, 1700 mm height) tubing closed with a flat 20 mm thick PVC bottom. They were built by AFE consulting, France. Tap water (for process and for cleaning) was supplied by a pump and delivered at the top of the cylinders through a rotating nozzle. Agitation and oxygen supply in each cylinder were provided by airflow bubbling from the bottom of the cylinder, at a rate of 0.4 volume of air per volume of culture per minute (Air-lift system). The air was supplied through a 400 mm diameter ring of 16 mm diameter PVC tubing pierced with 500 holes (1.5 mm diameter). All other necessary inputs were introduced manually at the top of the two cylinders. 

Each cylinder was filled up with 500 L of the pilot-scale medium, and each inoculated with 8L culture as prepared above (Cf. Section 4.1.1). The temperature of the culture was maintained between 28 and 30 °C using automated control from an immersed stainless steel electric heater (2 kW for 500 L) and a temperature probe, while pH was maintained above 4.5 by manual pH measurement and addition of 10 N NaOH solution as needed. Three cultures were also run in 330 L cylinders (260 L cultures) in similar conditions. Protein and total lipids analysis were obtained from these three cultures.

### 4.6. Analysis

#### 4.6.1. Dry Cell Weight (DCW) 

Culture was filtered through Whatman GF/F glass fiber filter, rinsed with the same volume of an ammonium formate solution (0.26 M) and dried at 105 °C for 24 h. 

#### 4.6.2. Monitoring of Culture Concentration and Cellular Parameters

Concentration and cellular parameters (size, complexity, lipid content) of *A. mangrovei* were measured using an Easy-Cyte Plus 6HT flow cytometer (Guava Merck Millipore^®^, Darmstadt, Germany) equipped with a 488 nm blue laser, detectors of forward (FSC) and side (SSC) light scatter, and three fluorescence detectors: green (525/30 nm), yellow (583/26 nm) and red (680/30 nm). Cell morphological variables, i.e., forward scatter (FSC) and side scatter (SSC), were used to identify and select the *A. mangrovei* cell population. FSC and SSC give, respectively, information on the relative size and complexity of cells [34]. The flow cytometry measurements were performed on fresh (living) samples. The BODIPY probe (BODIPY 505/515 FL; Molecular Probes, Invitrogen, Eugene OR, USA, final concentration of 10 mM), which stains lipid droplets/bodies within Thraustochytrid cells, was used as a proxy of lipid reserves [34]. The green fluorescence emitted was proportional to the quantity of lipid reserve present in the cells. Measurements were performed at a flow rate of 59 µL.min^−1^. Concentration of Thraustochytrid was given as cells per mL, and cellular parameters (FSC, SCC, and lipid reserve) were expressed as fluorescence intensity in arbitrary units (A.U).

#### 4.6.3. Nutrient Content Analysis 

Glucose concentration was measured using the DNS colorimetric method. Dissolved ammonia in medium was measured following Hansen and Koroleff [35] and Eppley et al. [36]. Dissolved phosphate in medium was measured following Murphy and Riley [37].

#### 4.6.4. Lipid Content Analysis

- Lipid Extraction:

Fifty milligrams of freeze-dried Thraustochytrid biomass were extracted in 6 mL of solvent mixture (CHCl_3_:MeOH, 2:1, *v:v*) directly added into glass vials [38]. Extracts were flushed with nitrogen gas, vortexed, and sonicated for 10 min to ensure complete lipid extraction and stored at −20 °C overnight for further analysis.

- Separation of polar lipids (PL) and neutral lipids (NL):

To fractionate NL and PL, 1 mL of total lipid (TL) extract was evaporated to dryness under nitrogen, recovered with 3 washes of 0.5 mL of CHCl_3_:MeOH (98:2, *v:v*; final volume 1.5 mL) and spotted at the top of a silica gel column (40 mm × 4 mm, silica gel 60A 63–200 µm rehydrated with 6% H_2_O, 70–230 mesh, Sigma-Aldrich, Darmstadt, Germany). The NL was then eluted using CHCl_3_:MeOH (98:2, *v:v*; 10 mL) and PL fraction with methanol (20 mL). Both were collected in glass vials containing an internal standard (C23:0, 2.3 µg). Lipid fractions were then stored at −20 °C under nitrogen atmosphere until further analysis.

- Fatty acid analysis in total lipids (TL), polar lipids (PL) and neutral lipids (NL):

Polar and neutral fractions or total lipid extracts were dried under vacuum with an evaporator (Genevac). Dried lipid fractions were saponified with 1 mL of KOH:MeOH (0.5 M) heated for 30 min at 80 °C, then they were transesterified with 800 µL of MeOH:H_2_SO_4_ (3.4%; *v*/*v*) heated at 100 °C for 10 min. The fatty acid methyl esters (FAME) formed were recovered in hexane. FAME of the NL of Thraustochytrid biomass were separated from other unwanted compounds (e.g., sterols and alcohols, potentially present in the NL fraction) using high performance liquid chromatography (HPLC) equipped with two columns (LiChrospher Si 60 and LiChrospher 100 DIOL, both 5 µm). A Dionex HPLC system (P680 pump AS-100 auto sampler, UVD170U UV detector with deuterium lamp, Foxy fraction collector), was used. Details on the analytical methods (i.e., solvent proportions, flow rate) can be found in [39]. The purified FAME were recovered in new vials for gas chromatography analysis.

FAME composition was analyzed by gas chromatography coupled to a flame ionization detector (GC-FID; Varian CP8400 gas chromatograph, Agilent). Samples (2 µL) were injected at 250 °C in splitless mode at an oven temperature of 60 °C, with hydrogen as the carrier gas. Detector temperature was set at 280 °C. The GC was equipped with a ZB-WAX column (30 m in length, 0.25 mm internal diameter, 0.25 µm film thickness, Phenomenex). The oven temperature was raised to 150 °C at 50 °C.min^−1^, to 170 °C at 3.5 °C.min^−1^, to 185 °C at 1.5 °C.min^−1^, to 225 °C at 2.4 °C.min^−1^ and then to 250 °C at 5.5 °C.min^−1^. FAME were identified by comparing their retention times to those of external commercial standard mixtures (S37 FAME Mix, PUFA n°1, and PUFA n°3, Supelco) using the software Galaxie 1.9.3.2 (Agilent). FAME peak area was converted into µg of FA based on the peak area of the internal standard C23:0. Concentrations of total neutral and polar lipids were expressed in mg.g^−1^ of biomass dry weight, and FA compositions of pilot-scale cultures were expressed in percentage of total fatty acids.

#### 4.6.5. Protein Analysis

Two milliliters of culture (cells+ supernatant) and of supernatant previously filtered through 0.2 µm were thawed and hydrolyzed with 1 N NaOH for 1 h at room temperature. Two hundred microliters of resulting protein extracts were analyzed following the Lowry method, using a BIO-RAD^TM^ kit (Ref: 5000116 DC protein assays reagent package). Standard curve was prepared using bovine serum albumin between 0.1 and 1.5 mg.mL^−1^. Whenever necessary, protein extracts were diluted to fit within the standard curve. 

#### 4.6.6. Conversion Yield

Yields were calculated as follows: [biomass concentration dry cell weight (g.L^−1^)]/[sum of initial organic substrate concentrations (g.L^−1^)].

#### 4.6.7. On Line Measurements

For pilot scale, the temperature, pH and polarographic dissolved oxygen probes, connected on a multi-parameters system KM 3000, were obtained from Sensortechnik Meinsberg GmbH, Germany. 

For 10 L cylinders in the lab, temperature, pH and polarographic oxygen probes from Ingold^TM^ were connected to an Applikon^TM^ controller ADI 1030 (Getinge AB, Göteborg, Sweden).

## Figures and Tables

**Table 1:** Yields and productivities observed in cultures. Yields were calculated as kg of biomass obtained per kg of initial organic substrate (see Section 4.6.6 for calculation description). When S.D. is indicated, biomass is mean of 3 replicates for flasks and carboys, and 2 replicates for pilot cylinders. DCW = dry cell weight.

	Culture Condition	Initial-Culture Medium	Feed	Culture Volume (L)	Culture Time (Days)	Biomass Concentration (g.L^−1^ ± S.D.)	Productivity (gDCW.L^−1^.day^−1^)	Yield
Flask-500 mL axenic	100 rpm	YEP modified	/	0.25	14	4.3	0.31	0.18
Flask-1 L axenic	100 rpm + 0.4 VVM	Glucose + Salts	/	0.8	5.67	3.21 ± 0.16	0.57	0.32
Carboy-20 L axenic	100 rpm + 0.4 VVM	YEP modified + 2.5% digestate	/	6	2.75	2.49	0.91	0.10
Cylinder-10 L non-axenic	Air-lift + 0.4 VVM-large bubbles	YEP modified + 2.5% digestate	/	6	2.75	2.82	1.03	0.12
Cylinder-10 L non-axenic	Air-lift + 0.8 VVM-small bubbles	YEP modified + 2.5% digestate	/	6	2.75	3.71	1.35	0.15
Cylinder-800 L non-axenic	Air-lift + 0.4 VVM	YEP modified + 2.5% digestate	/	500	1.58	5.21 ± 0.66	3.29	0.22
Cylinder-800 L non-axenic	Air-lift + 0.4 VVM	YEP modified + 2.5% digestate	+0.3X medium	500	1.58	7.15 ± 0.35	4.52	0.23
Cylinder-800 L non-axenic	Air-lift + 0.4 VVM	YEP modified + 2.5% digestate	+1X medium	500	2.57	14.34 ± 0.72	5.57	0.30

## Data Availability

Not applicable.

## Supplementary Materials

The following supporting information can be downloaded at: https://www.mdpi.com/article/10.3390/md20080499/s1. It includes three reports named: Interreg NWE ALG-AD project Seasonal variation of algal biomass cultivated using nutrient rich digestate; Interreg NWE ALG-AD project Safety analysis digestate and algal biomass produced by the three ALG-AD pilots; Interreg NWE ALG-AD project A Regulatory Review on the Use of Digestate to Cultivate Algal Biomass for Animal Feed. One table (S1) and three figures (S1, S2, and S3) are also in a supplementary file with the following captions: Table S1: Chemical characterization of digestate from COOPERL organic biological waste (pig manure) in Lamballe (Brittany, France); Figure S1: Percentages of dissolved oxygen of *A. mangrovei* cultivated in 800 L cylinder at 0, 3, 6, 9, and 24 h (*n*=11 batches, error bars= S.D.)*;* Figure S2: Mean values of *A. mangrovei* culture temperature in 800 L cylinder at 0, 3, 6, 9 and 24 h (*n*=11 batches, error bars= S.D.)*;* Figure S3: Mean values of *A. mangrovei* culture pH in 800 L cylinder at 0, 3, 6, 9 and 24 h (*n*=11 batches, error bars= S.D.).
