# Supplementary material for: Scale-Up to Pilot of a Non-Axenic Culture of Thraustochytrids Using Digestate from Methanization as Nitrogen Source
Source: Mar Drugs. 2022 Aug 2;20(8):499. doi: 10.3390/md20080499 (PMC9410245; doi:10.3390/md20080499)
Supplement: Supplementary file 1 [file marinedrugs-20-00499-s001.zip › marinedrugs-1809880-reports/Interreg NWE ALG-AD project A Regulatory Review on the Use of Digestate to Cultivate Algal Biomass for Animal Feed.pdf]

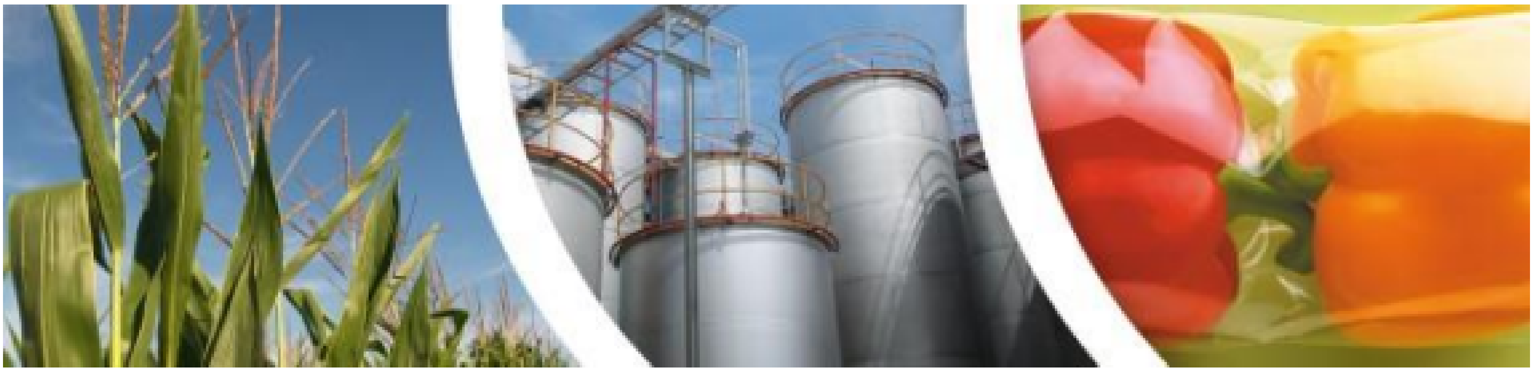

A Regulatory Review on the Use of Digestate to Cultivate Algal Biomass for Animal Feed

Prepared by NNFCC for ALG-AD

June 2020

Release Date: June 2020

Author(s): Sotirios Sourmelis Consultant

Robert Horton Research Analyst

Reviewer(s): Lucy Hopwood Lead Consultant for Bioenergy & Anaerobic Digestion

Disclaimer

While NNFCC considers that the information and opinions given in this work are sound, all parties must rely on their own skill and judgement when making use of it. NNFCC will not assume any liability to anyone for any loss or damage arising out of the provision of this report.

NNFCC is a leading international consultancy with expertise on the conversion of biomass to bioenergy, biofuels and biobased products.

NNFCC, Biocentre, York Science Park, Innovation Way, Heslington,
York, YO10 5NY
Phone: +44 (0)1904 435182
Fax: +44 (0)1904 435345
Email: enquiries@nnfcc.co.uk
Web: www.nnfcc.co.uk

Contents

1 Introduction..

.....................4 2 European legislation applicable to the ALG-AD value chain...5 2.1 Regulation on fertilising products – (EC) No 2019/1009...5 2.2 Regulations applicable to microalgae production...7 2.2.1 The Common Fisheries Policy ....7 2.2.2 Alien Species Legislation – (EC) No 708/2007 ....8 2.2.3 Environmental Impact Assessment – (EC) No 2011/92..9 2.2.4 Production and labelling of organic products – (EC) No 2018/848...10 2.3 Regulation on animal by-products and derived products – (EC) No 1069/2009 ....12 2.4 Regulations that apply to microalgae-based feed applications...15 2.4.1 Placing on the market and use of feed – (EC) No 767/2009 ....16 2.4.2 Feed Hygiene – (EC) No 183/2005 ....17 2.4.3 Undesirable substances in animal feed – Directive 2002/32/EC..18 2.4.4 Official Controls ....19 3 Implications of the EU legislation on the commercialisation of ALG-AD technology ....20 3.1 Key regulatory issues affecting the introduction of microalgae to the feed market ....21 3.2 Additional steps required for the roll-out of the ALG-AD technology...22 3.3 Key regulatory issues affecting the organic certification of microalgae...23 4 Conclusions ....24 5 References..29 6 Appendices ....31 6.1 Appendix 1: List of Competent Authorities ....31 6.2 Appendix 2: Procedure for the authorisation of an alternative method..33 6.3 Appendix 3: Guidelines for the distinction of different types of feed...34 6.4 Appendix 4: Mandatory labelling requirements for feed materials 35

CONFIDENTIAL

1 Introduction

Throughout the EU, anaerobic digestion (AD) is used as a means of managing food waste, livestock waste and other agricultural wastes via their conversion into biogas and nutrient-rich digestate (NRD).

Whilst biogas is the primary product of this process, digestate is widely recognised as a valuable by product and is used across the continent in fertiliser applications. Spreading of excess digestate is prohibited as it can have negative and potentially damaging impacts on the environment, with the most significant risk being posed to water resulting from eutrophication. Therefore, strict limits are imposed on the use of fertilisers around Europe and it is often not possible to return all digestate produced in AD facilities back to land, thus causing a build-up of material with no alternative application.

The Interreg North-West EU ALG-AD project intends to integrate microalgae cultivation plants with existing AD facilities, to create value from excess Nutrient-Rich Digestate (NRD) that currently has no industrial application. Nutrients contained within digestate could be used to feed microalgae, which in turn will be used as sustainable protein source for animal feed.

This study explores the EU legislation across the entire microalgae production value chain, focusing on how the requirements apply to the use of microalgae as a component of compound feed[1]; the main market being targeted by ALG-AD. The report is intended to inform project partners initially, then a broader set of stakeholders and end users with an interest in this value chain. This report specifically documents and summarises:

1. relevant legislation that producers and users of the ALG-AD project outputs (animal feed) would be required to comply with,
2. any regulatory issues affecting the introduction of NRD-grown, enzymatically-treated microalgae[2] as a non-GMO feed material into the animal feed market (including agricultural livestock, aquaculture and non-food producing animals)[3], and
3. the steps required for the roll-out of the ALG-AD technology, from a regulatory perspective.

A follow-up report will also examine how different regulations may apply to select alternative markets, for the ALG-AD developers to review when considering the development of other novel products, such as pharmaceuticals, nutraceuticals, and cosmetics. The follow-up report will also draw together a series of policy recommendations, to address barriers identified during the initial legislative review.

[1] 'Compound feed' means a mixture of at least two feed materials, whether or not containing feed additives, for oral animal feeding in the form of complete or complementary feed [19].

[2] The end-product will be microalgae biomass that has undergone an enzymatic treatment to break down cell walls and release peptides. Therefore, different components (e.g. proteins and lipid fractions) will not be separated as part of the project, and the product will be used in its whole form in compound feed formulations.

[3] EU legislation on animal feed applies to feed for food-producing animals, including farmed fish, and also non-food producing animals, including pets, zoo and circus animals and creatures living freely in the wild [28].

2 European legislation applicable to the ALG-AD value chain

This chapter deals with the EU legislation that applies to the entire microalgae production value chain,

from digestate production to microalgae cultivation and product application. However, it specifically focuses on the rules that apply to microalgae for use as animal feed, as this is the main market being targeted by ALG-AD; accessing different markets may require changes in the production process and, as a result, different legislation may apply.

Macroalgae, which are commonly referred to as ‘seaweeds’, are large and multicellular marine organisms that can be seen without the aid of a microscope and resemble big plants in the sea. Whereas microalgae, which are the targeted organisms within the ALG-AD project, are microscopic (mainly unicellular) photosynthetic organisms, commonly known as ‘phytoplankton’.

In general terms, microalgae and macroalgae fall within the same legislation when destined for animal feed production, although specific requirements sometimes differ on a case by case basis. As described in more detail in the following sections, animal feed legislation is typically common across both types of algae as the emphasis is put on the end-product, the production process and the operation of production facilities. Similarly, relevant legislation on animal by-products and derived products focus on the origin of feed materials, rather than their conversion to algae. Other legislation focuses on aquaculture and aquatic organisms in general, without using the term ‘algae’ and as such does not differentiate between microalgae and macroalgae. All such regulations and the nuances thereof are reviewed in detail in the following sections.

2.1 Regulation on fertilising products – (EC) No 2019/1009

Regulation (EU) 2019/1009 of the European Parliament and of the Council of 5 June 2019 laying down rules on the making available on the market of EU fertilising products and amending Regulations (EC) No 1069/2009 and (EC) No 1107/2009 and repealing Regulation (EC) No 2003/2003

This regulation focuses primarily on fertilising products, but it is the only EU legislation to make legal provisions for the safe generation of digestate from anaerobic digestion. Therefore, it is important to understand the requirements, as similar ones could potentially be applied to the safe usage of digestate to grow microalgae.

More specifically, this regulation lays down the conditions for the production and supply of fertiliser products, in order to minimise risks to human and animal health, and to avoid damage to the natural environment through application of fertilisers. It defines a fertilising product as “a substance, mixture, micro-organism or any other material, applied or intended to be applied on plants or their rhizosphere or on mushrooms or their mycosphere, or intended to constitute the rhizosphere or mycosphere, either on its own or mixed with another material, for the purpose of providing the plants or mushrooms with nutrient or improving their nutrition efficiency” [1].

Annex I of the regulation defines possible Product Function Categories (PFCs) for fertilising products, determining their role as nutrient sources, soil improvers, acidity regulators, etc. Annex I also lists the quality control requirements for each PFC; namely, the expected chemical composition, including minimum expected concentrations of certain nutrients, and maximum permitted concentrations of harmful substances.

Annex II of this regulation lists materials that can be used as fertilisers, broken into Component Material Categories (CMCs). Most conventionally produced digestate falls into CMC5, whereas

digestate from fresh plant material is considered CMC4. The Annex includes a strict list of feedstocks that may be used to produce digestate intended for use as fertiliser, as follows:

1. Separately collected bio-waste according to the definition in directive 2008/98/EC [2], which includes:
   a. Food waste from households, restaurants, caterers, etc
   b. Garden or park waste
   c. Comparable waste from food-processing plants
2. Products derived from animal by-products referred to in Article 32 of Regulation (EC) No 1069/2009 for which an endpoint in the manufacturing chain has been determined[4]. This is either the point at which they become subject to other regulation[5] or the point at which they no longer pose any significant risk to public or animal health[6].
3. Digestion additives that are necessary to improve the process performance or the environmental performance of the digestion process[7]
4. Living or dead organisms or parts thereof that are unprocessed or processed only by manual, mechanical or gravitational means, by dissolution in water, by flotation, by extraction with water, by steam distillation or by heating solely to remove water, or which are extracted from air by any means, except:
   a. Organic fraction of municipal solid waste[8]separated through mechanical, physicochemical, biological and/or manual treatment
   b. Sewage sludge, industrial sludge, dredging sludge
   c. Animal by-products or derived products without a determined endpoint in the manufacturing chain
5. Any material listed in points (1), (2) or (3) that has previously been composted or digested, and contains no more than 6 mg/kg dry matter of PAH16

In addition to this, Annex II states the conditions under which the digestion must take place and defines the quality control requirements for the resulting digestate. It is the responsibility of the Competent Authority in each EU member state to determine whether fertilising products from each specific plant are compliant, but the responsibility of the manufacturer to ensure compliance.

The procedure for testing for conformity is laid out in Annex IV of the regulation. However, where harmonised standards exist, the regulation assumes compliance if the fertilising product in question is compliant with other such standards informed by the relevant EU regulation. For example, in the UK, the standard for digestate is the publicly available specification (PAS110); PAS110 certified digestate is assumed to be compliant with the EU regulation.

[4] A full definition of an endpoint in the manufacturing chain is given in section 3.
[5]For example: cosmetics are no longer considered ABPs once they are subject to Directive 76/768/EEC. [6]For example: organic fertilisers, soil improvers, pet food and other derived products not intended for human consumption. [7] Provided that the total concentration of all additives does not exceed 5% of the total input material weight and these additives are registered pursuant to Regulation (EC) No 1907/2006, unless they are explicitly covered by one of the registration obligation exemptions provided for by Annex IV or by points 6, 7, 8 or 9 of Annex V of the same regulation. [8] May include food waste, paper, textiles, etc

2.2 Regulations applicable to microalgae production

The European Union does not have a single policy dedicated to algae, or more specifically microalgae production, but rather a suite of policies that touch upon different elements of production, either directly or indirectly [3]. Moreover, relevant policies and regulations do not necessarily apply to all microalgae producers or processors, due to differences in the end-product or the size and location of the operations.

For example, the microalgae specifically produced as part of the ALG-AD project are not genetically modified, and therefore EU directives on GM algae (2009/41/EC and 2001/18/EC) do not apply in this instance [4], [5]. In addition, considering that microalgae developed as part of the project are produced in closed reactors on land, the Maritime Spatial Planning Directive does not apply, as it is only relevant when algae farming happens at sea [6].

This section presents the key European policies related to production and harvesting of microalgae, specifically in relation to the ALG-AD project and the end markets being considered.

2.2.1 The Common Fisheries Policy

The Commission's Common Fisheries Policy (CFP) intends to boost the aquaculture sector and ensure that it is environmentally, economically, and socially sustainable, and provides a source of healthy food for EU citizens. Its goal is to foster a dynamic fishing industry and ensure a fair standard of living for fishing communities. The CFP was first introduced in the 1970s and went through successive reforms, the most recent of which took effect on 1 January 2014.

In 2013, the Commission also published Strategic Guidelines presenting common priorities and general objectives at EU level. The four priority areas identified are presented below [6]:

1. Reducing administrative burdens
2. Improving access to space and water
3. Increasing competitiveness
4. Exploiting competitive advantages due to high quality, health and environmental standards.

Based on the guidelines, the Commission and EU countries are collaborating to help increase the sector's production and competitiveness. In 2014-1015, the Member States developed Multiannual National Strategic Plans to promote aquaculture and propose actions to address the four priority areas identified by the Commission [7]. The Commission is helping with the identification of bottlenecks but also facilitates cooperation, coordination and exchange of best practices between EU countries. The strategic guidelines on aquaculture are currently being revised.

This regulation can be potentially supportive to ALG-AD, as aquaculture feed production is one of the main targeted markets for micro-algae cultivated as part of the project. This policy can also be applicable to ALG-AD, where cultivation of micro-algae is considered as an aquaculture activity.

2.2.2 Alien Species Legislation – (EC) No 708/2007

Council Regulation (EC) No 708/2007 of 11 June 2007 concerning use of alien and locally absent species in aquaculture

Note: This section investigates whether the cultivation of Algae in tubular reactors can be considered as an aquaculture activity for the purposes of the Alien Species Legislation. This should be distinguished from the use of micro-algae as feed in aquaculture.

Under this regulation [7], aquaculture operators intending to undertake the introduction of an alien species or the translocation of a locally absent species, not covered by Article 2(5), shall apply for a permit from the competent authority of the receiving Member State[9]. Based on this Regulation, alien species are defined as "…species or subspecies of an aquatic organism occurring outside its known natural range and the area of its natural dispersal potential", while locally absent species are defined as "a species or subspecies of an aquatic organism which is locally absent from a zone within its natural range of distribution for biogeographical reasons".

Alien Species Legislation refers to Council Regulation (EC) No 1198/2006 for the definition of aquaculture, meaning "the rearing or cultivation of aquatic organisms using techniques designed to increase the production of the organisms in question beyond the natural capacity of the environment; the organisms remain the property of a natural or legal person throughout the rearing or culture stage, up to and including harvesting". Furthermore, a 'closed aquaculture facility' means "a facility where aquaculture is conducted in an aquatic medium, which involves recirculation of water and which is separated from the wild aquatic medium by barriers preventing the escape of reared specimens or biological material that might survive and subsequently reproduce" suggesting different growing media such as tubular reactors and algal ponds would all fall within this definition.

In addition, Alien Species Legislation gives a definition of aquatic organisms to mean "any species living in water belonging to the animalia, plantae and protista kingdoms, including any part, gametes, seeds, eggs or propagules of their individuals that might survive and subsequently reproduce". The definition above covers all the Kingdoms of the Eukaryota (animalia, plantae, and Protista). Alien species legislation covers all aquaculture activities irrespective of their size, characteristics, and alien species used.

Microalgae would be considered aquatic organisms, while their cultivation systems can be considered as aquaculture. Although the microalgae will be cultivated outside their known natural range in closed land-based reactors, they will be "…separated from the wild aquatic medium by barrier…" as required by the legislation; therefore, it is likely the alien species legislation will not apply. However, as this relies heavily on interpretation of a number of definitions it is advised that developers should discuss individual cases with the competent authority of the receiving Member State, and subsequently apply for a permit if necessary.

[9] Competent authorities can vary in number, depending on the Member State where the application is made, meaning that developers should investigate the one relevant to the particular location of the plant. For example, the competent authority in England and Wales is the Fish Health Inspectorate at the Centre of Environment, Fisheries, and Aquaculture Science (Cefas) [29]; in France applications should be made at regional (county) level [30], while in Belgium, both the Federal authority and the Regions are competent for implementing the Regulation [31].

If required, applications may be submitted for multiple introductions and/or translocations to take place over a period of no longer than seven years, typically on a per site basis. The applicant[10] shall submit with the application a dossier following the indicative guidelines listed in Annex I of the regulation, while the advisory committee shall give an opinion on whether the application contains all the information required to assess whether the proposed movement is routine[11] or non-routine[12], and shall inform the competent authority of its opinion.

In the case of routine movements, the competent authority may grant a permit, indicating, where applicable, the requirement for quarantine or pilot release. In the case of non-routine movements, an environmental risk assessment shall be carried out as outlined in Annex II of the regulation. An environmental risk assessment is also required for discharge of effluents and wastewater from microalgae cultivation systems, to mitigate risks of target species and associated organisms being released inappropriately.

Finally, it is important to note that the measures provided for in the Regulation (EC) No 708/2007 should be without prejudice to Council Directive 85/337/EEC of 27 June 1985 on environmental impact assessment. This means that the obligation to apply for a permit under the Alien Species Legislation does not replace any requirement for an Environmental Impact Assessment, depending on thresholds/criteria or a case by case examination of each Member State.

2.2.3 Environmental Impact Assessment – (EC) No 2011/92

Directive 2011/92/EU of the European Parliament and of the Council of 13 December 2011 on the assessment of the effects of certain public and private projects on the environment

An Environmental Impact Assessment may also be required for setting up a new microalgae cultivation plant, based on the requirements of 2011/92/EU[13] [8]. Aquaculture projects are listed in Annex II of the Directive[14], meaning that national authorities have to decide whether an EIA is needed regardless of the nature or type of cultivation system, by conducting a "screening procedure", which determines the effects of projects on the basis of thresholds and criteria or a case by case evaluation.

There is an additional aspect of this; in accordance with (EU) 2018/848 (see 2.2.4), an environmental impact assessment that is appropriate to the production unit is compulsory for any new operators applying for organic production and producing more than 20 tonnes of aquaculture products per year. This is necessary to ascertain the conditions of the production unit and its immediate environment and the likely effects of its operation.

[10] The natural or legal person or entity proposing to conduct the introduction or translocation of an aquatic organism. [11] 'Routine movement' means the movement of aquatic organisms from a source which has a low risk of transferring non-target species and which, on account of the characteristics of the aquatic organisms and/or the method of aquaculture to be used, for example closed systems, does not give rise to adverse ecological effects.

[12] 'Non-routine movement' shall mean any movement of aquatic organisms which does not fulfil the criteria for routine movement.

[13] The initial Directive of 1985 and its three amendments have been codified by DIRECTIVE 2011/92/EU of 13 December 2011. Directive 2011/92/EU has been amended in 2014 by DIRECTIVE 2014/52/EU.

[14] Projects listed in Annex I are considered as having significant effects on the environment and require an EIA.

2.2.4 Production and labelling of organic products – (EC) No 2018/848

Regulation (EU) 2018/848 of the European Parliament and of the Council of 30 May 2018 on organic production and labelling of organic products and repealing Council Regulation (EC) No 834/2007

Microalgae producers can potentially obtain an organic label by complying with the rules laid down in this regulation [9], which comes into force on 1 January 2021 [10]. It should be noted that this regulation does not distinguish microalgae from macroalgae, as there is only reference to "algae" as a broader term. However, there is certainty that both categories are covered as existing rules on organic production and labelling, set by Regulation 834/2007, apply to both macroalgae and microalgae[15].

Operators shall comply with the general production rules laid down in Chapter II of regulation 2018/848, which, among other things, include:

(a) taking preventive measures at each stage of production, preparation and distribution,
(b) prohibition of the use of GMOs,
(c) the use in organic production only of products or substances that are authorised and included in restrictive lists[16],
(d) taking proportionate and precautionary measures to avoid contamination with unauthorised substances.

According to Article 15 of Regulation 2018/848, operators that produce (micro- and macro-) algae and aquaculture animals shall comply with additional requirements, which are presented in Part 3 of Annex II. The requirements that are most likely to apply to terrestrial microalgae producers are listed below:

(a) Operations shall be situated in locations that are not subject to contamination with products or substances not authorised for use in organic production, or with pollutants that would compromise the organic nature of the products.
(b) Operators shall provide a sustainable management plan proportionate to their production unit for aquaculture and algae harvesting, which shall be updated annually[17].
(c) Defensive and preventive measures taken against predators shall be recorded in the sustainable management plan.
(d) Aquaculture and algae business operators shall draw up, as part of the sustainable management plan, a waste reduction schedule to be put in place at the commencement of operations. Where possible, residual heat shall be supplied from renewable sources.
(e) Only nutrients of plant or mineral origin authorised for organic production may be used. (f) In facilities on land where external nutrient sources are used, the nutrient levels in the effluent water shall be verifiably the same, or lower, than the inflowing water.

It should be noted that, according to Article 10, when a farm wishes to produce organic products, it has to go through a conversion period during which it must be managed according to organic production rules, although its products at this stage cannot be marketed as organics[18]. Based on the

[15] Guidance on the rules for the implementation of Regulation (EC) No 834/2007 can be found in Regulation 2016/673. [16] Given that the use of such external inputs in non-organic production is not prohibited by either the EU or national law. [17] This plan shall detail the environmental effects of the operation and the environmental monitoring to be undertaken, and shall list the measures to be taken to minimise negative impacts on the surrounding aquatic and terrestrial environments, including, where applicable, nutrient discharge into the environment per production cycle or per annum. [18] According to Article 10 (2), the conversion period shall start at the earliest when the operator that produces algae has notified the activity to the competent

authorities in the Member State in which the activity is carried out, and in which that holding is subject to the control system.

specific requirements that apply to algae producers (Part 3 of Annex II), the conversion period for a production unit for algae cultivation shall be six months or one full production cycle, whichever is longer.

Finally, organic production is only credible if accompanied by effective verification and controls at all stages of production, processing and distribution. As such, operators and groups of operators, except those that sell pre-packed organic products directly to the final user and potentially those referred to in article 35(8)[19], shall be subject to a verification of compliance through physical on-the-spot inspections. The frequency of such inspections shall be at least once a year or every 2 years if no fraud has been detected over the previous 3 years[20].

[19] Animal feed operators do not fall into this category.

[20] According to Article 41, if a control body suspects an operator of trying to place a non-authorised product on the market as 'organic', it must formally investigate and temporarily ban the placing on the market of that product pending the investigation's

outcome. In the event of serious or repeated infringements, operators may be prohibited from selling products described as organic for a given period or may have their certificate withdrawn.

2.3 Regulation on animal by-products and derived products – (EC) No 1069/2009

Regulation (EC) No 1069/2009 of the European Parliament and of the Council of 21 October 2009 laying down health rules as regards animal by-products and derived products not intended for human consumption and repealing Regulation (EC) No 1774/2002 (Animal by-products Regulation)

In order to reduce potential adverse effects to human and animal health, this regulation aims to lay down strict controls on how animal by-products (ABP) can be processed, and what end-uses are acceptable for them [11]. As animal by-products are often used as feedstock for AD, biogas generators must abide by these regulations, as must producers of any products derived from animal by-products.

Acceptable disposal or treatment methods for ABPs are determined by what Category they fall into, based on the risks they pose. Any mixture of ABPs from multiple categories is considered to be of the lowest-numbered category and thus subject to the strictest available regulations.

- Category 3 (classed as Low Risk):
    - carcasses or body parts passed fit for humans to eat, at a slaughterhouse
    - products or foods of animal origin originally meant for human consumption but withdrawn for commercial reasons, not because it's unfit to eat
    - domestic catering waste
    - shells from shellfish with soft tissue
    - eggs, egg by-products, hatchery by-products and eggshells
    - aquatic animals, aquatic and terrestrial invertebrates
    - processed animal proteins (PAP)

- Category 2 (classed as High Risk):
    - animals rejected from abattoirs due to having infectious diseases
    - carcasses containing residues from authorised treatments
    - unhatched poultry that has died in its shell
    - carcasses of animals killed for disease control purposes
    - carcasses of dead livestock
    - digestive tract content
    - manure

- Category 1 (classed as High Risk):
    - carcasses and all body parts of animals suspected of being infected with TSE (transmissible spongiform encephalopathy)
    - carcasses of wild animals suspected of being infected with a disease that humans or animals could contract
    - carcasses of animals used in experiments
    - parts of animals that are contaminated due to illegal treatments
    - carcasses and body parts from zoo and circus animals or pets
    - body parts that pose a particular disease risk, e.g. cows' spinal cords
    - international catering waste (from international transport operators, e.g. airlines)

Categories 2 and 3 are acceptable for use in biogas generation. However, only Category 3 ABPs can

be used to produce animal feed, with the exception of catering (food) waste. This exception specifically applies to catering waste, which is likely to contain animal by-products, and not food supply chain

waste from non-ABP facilities, such as vegetable wastes; however, specific streams need to be well defined. Despite being listed as a Category 3 ABP, catering waste is specifically prohibited from being used to make animal feed, but may, if authorised by a member state's "competent authority", be fed to fur animals (Article 31) that will not re-enter the food chain.

It should be noted that according to Article 7(3) amendments to the lists of categorisation of animal by-products and derived products are possible, to account for progress in science and technology, but this requires a risk assessment to be carried out by an appropriate scientific institution, such as EFSA and the European Medicine Agency.

According to Article 5 of this regulation, for certain end-uses of ABPs, an end point in the manufacturing chain is defined, after which the regulation does not apply. For products referred to in Article 33 (e.g. cosmetic products and veterinary medical products), this is the point at which they become subject to other regulation[21], while for products referred in Articles 32 (organic fertilisers and soil improvers), 35 (pet food), and 36 (other derived products), this is the point at which they no longer pose any significant risk to public or animal health[22]. However, according to article 35, there is an additional requirement that pet food should not be derived from materials referred to in Article 10 (n), (o), and (p), which include catering (food) waste.

Considering the inclusion of an end point in the manufacturing chain for organic fertilisers that do not pose any significant risk to human and animal health, it is unclear whether digestate, or algae grown using digestate, continue to be classified as ABP-derived products and thus subject to the regulation, when used in animal feed applications. However, to the best of our understanding, an end point in the manufacturing chain cannot be applied to products derived from Category 2 feedstock and intended to be used in animal feed applications.

Firstly, there is no such end point defined for placing animal feed on the market, referred to in Article 31; this regulation applies to both animal by-products and derived products[23]. Moreover, as mentioned earlier, only Category 3 material should be used for the manufacturing of feed for farmed animals. However, the preamble (22) of the Regulation 1069/2009 lays down that, for reasons of legal certainty and proper control of potential risks, an end point in the manufacturing chain should be determined for products that no longer have direct relevance for the safety of the feed chain. Article 11 of this Regulation, which lays down additional restrictions on disposal and use of animal by products and derived products, also prohibits the feeding of farmed animals, other than fur animals, with catering waste or feed material containing or derived from catering waste, which, along with animal manure, are the most commonly used AD feedstock that are under the scope of this regulation.

Additionally, in Regulation 1069/2009, 'organic fertiliser' and 'soil improvers' are defined as materials of animal origin used to maintain or improve plant nutrition and the physical and chemical properties and biological activities of soils, either separately or together. Microalgae do not fall within the

[21] For example: cosmetics are no longer considered ABPs once they are subject to Directive 76/768/EEC. [22] Originally, Article 5 did not include any provision for organic fertilisers, but it has been amended in 2019, introducing an end manufacturing point for the ones that no longer pose significant risks to human and animal health. [23]‘**Derived products’** means products obtained from one or more treatments, transformations or steps of processing of animal by-products.

definition of plants[24], given in Regulation (EU) 2016/2031 [12], and therefore digestate used for their cultivation cannot be strictly regarded as organic fertiliser or soil improver.

It should be mentioned that only for the purpose of PFC4 in Annex I of Regulation 2019/1009[25], which lays down rules on the market for EU fertilising product, algae are included in the definition of plants. However, it is specifically mentioned in this regulation that digestate cannot be used as a fertiliser when obtained from the anaerobic digestion of animal by-products or derived products falling within the scope of Regulation (EC) No 1069/2009 and for which no end point in the manufacturing chain has been determined.

In order to take account of the related progress in science and technology, according to preamble 44, novel technologies that offer advantageous ways of animal by-products treatment should be authorised as ‘alternative methods’ for the disposal or use of animal by-products throughout the community. Applications can be submitted to the competent authority of the Member State where developers intend to use the alternative method, while the application will also be reviewed by the European Food Safety Authority (EFSA); a list of the competent authorities for the Member States of interest is presented in Appendix 1. The procedure for the authorisation of an alternative method of use or disposal is presented in Appendix 2, while EFSA has also published a statement on the format for applications [13]. EFSA will assess whether the process reduces risks to human and animal health from ABPs and will decide whether to authorise the use of this alternative method for the processing and/or use of ABPs.

[24] '**Plants**' means living plants and the following living parts of plants: (a) seeds, in the botanical sense, other than those not intended for planting; (b) fruits, in the botanical sense; (c) vegetables; (d) tubers, corms, bulbs, rhizomes, roots, rootstocks, stolons; (e) shoots, stems, runners; (f) cut flowers; (g) branches with or without foliage; (h) cut trees retaining foliage; (i) leaves, foliage; (j) plant tissue cultures, including cell cultures, germplasm, meristems, chimaeric clones, micro-propagated material; (k) live pollen and spores; (l) buds, budwood, cuttings, scions, grafts.

[25] Details on this regulation are given in section 2.1.

CONFIDENTIAL

2.4 Regulations that apply to microalgae-based feed applications

Legislation on animal feed is harmonised at European Union (EU) level and applies to a wide range of animal feed businesses and activities, including the manufacture, sale, and supply stages of the value chain. It applies to both feed for food-producing animals, including farmed fish, and also to feed for non-food producing animals, such as pets, zoo animals, as well as creatures living freely in the wild.

EU Regulation 178/2002 provides a framework for the development of food and feed legislation at Community and national level and lays down procedures in matters of food safety [14]. This regulation, also established by the European Food Safety Authority (EFSA), applies to all stages of production, processing and distribution of food and feed. In accordance with the principles laid down in 178/2002, the EU animal feed legislation has been developed and divided according to the main set of regulations, which are reviewed in the following sections.

Before reviewing the animal feed legislation, it is important to recognise that there are regulations and rules in the legislation that do not apply to every type of animal feed. For assisting feed business operators to enforce and to apply the relevant legislation, the EU commission established guidelines for the distinction between feed materials, feed additives, biocidal products, and veterinary medical products [15].

Based on EU guidelines and the definitions of the different types of feed that are presented in Appendix 3, the products developed by ALG-AD partners can be considered as a feed material, as they are not chemically well-defined and their principal purpose is to meet the animals' nutritional needs, unlike feed additives. Furthermore, it cannot be classed as 'primary production', which is defined as "production of agricultural products, including in particular growing, harvesting, milking, rearing of animals (prior to their slaughter) or fishing resulting exclusively in products which do not undergo any other operation following their harvest, collection or capture, apart from simple physical treatment".

This chapter therefore only deals with the legislation applicable to feed materials, excluding, for example, Regulation 1831/2003, which establishes a common procedure for authorising feed additives and lays down rules for their placing on the market [16]. Further consideration of specific regulations of relevance to production of feed additives may be considered in future research; but feed materials have been identified as the priority category for consideration.

It is also important to note that the regulations on genetically modified feed (Regulation 1829/2003) [17] do not apply to the ALG-AD project outcomes, because the microalgae produced are not GMO, and therefore they are not considered in this study.

Finally, although not directly relevant as focussed on food as opposed to feed, Regulation (EU) 2015/2283[26] on novel foods could provide a useful basis upon which to build a case for expansion of feed-specific regulations. This regulation was updated in January 2018 to expand the categories and definitions of novel foods and to allow for generic authorisations of food groups, as well as now providing a "positive list" containing all authorised novel foods, simplifying procedures for the future.

[26] As of 1 January 2018, the new Regulation (EU) 2015/2283 on novel foods (the new Regulation) is applicable. It repeals and replaces Regulation (EC) No 258/97 and Regulation (EC) No 1852/2001 which were in force until 31 December 2017.

The new Regulation improves conditions so that food businesses can easily bring new and innovative foods to the EU market, while maintaining a high level of food safety for European consumers.

2.4.1 Placing on the market and use of feed – (EC) No 767/2009

Regulation (EC) No 767/2009 of the European Parliament and of the Council of 13 July 2009 on the placing on the market and use of feed, amending European Parliament and Council Regulation (EC) No 1831/2003 and repealing Council Directive 79/373/EEC, Commission Directive 80/511/EEC, Council Directives 82/471/EEC, 83/228/EEC, 93/74/EEC, 93/113/EC and 96/25/EC and Commission Decision 2004/217/EC

The EU has no system for pre-market authorisation of feed materials [18], but (EC) No 767/2009 addresses the placing on the market and use of feed within the European Community [19]. The objective of this regulation is to harmonise the conditions for the use of feed, in order to ensure a high level of feed safety and thus a high level of protection of public health.

According to this regulation, feed may only be placed on the market and used if it is safe and does not have a direct adverse effect on the environment or animal health. As such, a list of restricted or

prohibited feed materials is set out in Annex III of the same legislation, which includes, among other things, faeces and urine, which are common feedstocks in AD.

It is important to note that micro-algae-based feed materials, in general, are not included in the list of prohibited or restricted feed materials, meaning that they can be safely used in animal feed applications in the EU, given that they are not genetically modified, fulfil the requirements of Regulation (EC) No 1069/2009 on the use of animal by-products, and comply with Article 4 of Regulation (EC) No 767/2009, which lays down safety and marketing requirements.

The Joint Research Centre (JRC), the EU Commission's science and knowledge service, provides a list of micro-algae currently used in food or feed applications. This list includes species that are used for the production of feed materials as part of the ALG-AD project (Chlorella, Scenedesums, Desmodesmus). Chlorella has been given the GRAS (generally recognised as safe) status by the FDA (Food and Drug Administration) of the USA, while Scenedesums and Desmodesmus are regarded as not producing toxins (no toxins known) [20].

Regulation (EC) No 767/2009 also requires operators placing feed on the market to ensure that the feed is sound, genuine, unadulterated, fit for purpose, and of merchantable quality, as well as labelled, packaged, and presented in accordance. Labelling and packaging requirements are presented in detail in Appendix 4 of the regulation.

Regulation (EC) No 767/2009 has resulted in the establishment of two lists: ‘the Catalogue of Feed Materials’ and ‘the Register of Feed Materials’. Concerning the ‘Catalogue of Feed Materials’, its use by feed business operators is voluntary and its main purpose is to support proper labelling. The catalogue of feed materials is available in Commission Regulation (EU) No 68/2013 [21].

The Register concerns feed materials that are not listed in the Catalogue, as Regulation (EC) No 767/2009 states in article 24(6): “the person who, for the first time, places on the market a feed material that is not listed in the Catalogue shall immediately notify its use to the representatives of the European feed business sectors referred to in Article 26(1). The representatives of the European feed business sectors shall publish a Register of such notifications on the Internet and update the Register on a regular basis”.

2.4.2 Feed Hygiene – (EC) No 183/2005

Regulation (EC) No 183/2005 of the European Parliament and of the Council of 12 January 2005 laying down requirements for feed hygiene

This regulation lays down the general rules on feed hygiene, the conditions and arrangements to ensure traceability of feed, as well as the requirements for registration and approval of feed business operators [22].

This regulation introduces two general obligations that apply to all feed business operators. Firstly, feed business operators shall ensure that all stages of production, processing and distribution under their control are carried out in accordance with Community legislation, national law, and good practice. Secondly, they need to adopt procedures to keep the risk of contamination of feed as low as reasonably possible, although no specific limits are defined.

This regulation also introduces specific obligations that apply to feed-business operators at the level

of ‘primary production of feed’, as well as to those involved in transport storage and handling of primary products, or those who mix feed for the exclusive requirements of their own holdings without using additives, with the exception of silage additives.

According to Regulation 183/2005, ‘primary production of feed’ means “the production of agricultural products, including in particular growing, harvesting, milking, rearing of animals (prior to their slaughter) or fishing resulting exclusively in products which do not undergo any other operation following their harvest, collection or capture, apart from simple physical treatment”.

Although the produced microalgae could potentially be considered as primary products, the end product (feed material) has undergone an enzymatic treatment to break down cell walls and release peptides. To the best of our understanding, this treatment is considered as “biological” rather than “physical”, and therefore the end-product may not be considered as “primary”. As a result, these obligations may not be applicable to the project results, but further investigation on the validity of the statement above is recommended[27].

Feed business operators, apart from those at the level of primary production, must implement a HACCP (Hazard Analysis and Critical Control Points) system to identify where control is critical to ensure feed safety, in accordance with the requirements referred to in articles 6 and 7, while they should also comply with the provisions referred to in the Annex II of the Regulation.

As mentioned earlier, this regulation also introduces the compulsory registration and approval of all feed business establishments by the competent authority. Feed business operators shall not operate unless their establishments are registered and approved by the competent authorities.

Finally, this regulation encourages the development, dissemination, and use of both national and Community guides to good practice for hygiene or the application of HACCP principles [23]. It should be noted that these guides may be used on a voluntary basis by the feed business operators.

[27] Guidance on the implementation of the feed hygiene requirements can be found in Commission Notice (2019/C 225/01) [32].

2.4.3 Undesirable substances in animal feed – Directive 2002/32/EC

Directive 2002/32/EC of the European Parliament and of the Council of 7 May 2002 on undesirable substances in animal feed

Directive 2002/32/EC (in Annex 1) regulates maximum levels of undesirable substances in products intended for animal feed, such as heavy metals, dioxin, aflatoxin, and certain pesticides, while it also prohibits the dilution of contaminated feed materials [24]. This Directive (mainly Annex 1) has been regularly amended considering developments in scientific and technical knowledge; most notably (EU) 2019/1869 provides corrections regarding the maximum levels for certain undesirable substances in animal feed.

Indicative maximum levels of heavy metals for feed materials, including the ones destined for aquaculture, are presented in Table 1, as this is one of the main targeted markets by ALG-AD

partners. As shown in section 2.4.4, the official control of feed regarding the determination of undesirable substances is conducted on the basis of the methods of feed sampling and analysis covered by the Commission Regulation (EU) No 691/2013.

Table 1. Updated limits for heavy metals stated in Annex 1 of Directive 2002/32/EC

**Arsenic**	2* or 25**	*Specified in Commission Regulation (EU) 2019/1869*
**Lead**	10	*Specified in Commission Regulation (EU) 2019/1869*
**Mercury**	0.1* or 0.5**	*Specified in Commission Regulation (EU) 2019/1869*
**Cadmium**	1*** or 2****	*Specified in Commission Regulation (EU) 1275/2013*

*General figure, **Figure for feed materials that are fish, other aquatic animals and products derived thereof ***Feed materials of vegetable origin ****Feed materials of animal and mineral origin

The limits of Arsenic, Mercury and Cadmium are subject to interpretation, dependent on whether microalgae are considered aquatic animals, organisms or plant material under the circumstances in which they are produced.

In particular, while the general figure on maximum arsenic and mercury content for products used as feed materials is 2 and 0.1 mg/kg respectively, “fish, other aquatic animals and products derived thereof” are subject to higher arsenic and mercury maximum levels (25 and 0.5 mg/kg respectively). To the best of our understating, for the purposes of this regulation, micro-algae cannot be regarded as aquatic animals and therefore the general figure applies in both cases.

Similarly, in the case of Cadmium, different maximum levels apply depending on whether algae-based feed materials are considered of animal or plant origin. Therefore, it is recommended that specific limits should be confirmed with EFSA and the relevant national assurance body in each Member State.

Different heavy metal maximum levels apply for algae-based feed additives or for products intended to be used as complete feed.

2.4.4 Official Controls - (EC) No 882/2004

Regulation (EC) No 882/2004 of the European Parliament and of the Council of 29 April 2004 on official controls performed to ensure the verification of compliance with feed and food law, animal health and animal welfare rules (repealed)

‘Official controls’ are defined as any form of control that the competent authority or the Community performs for the verification of compliance with feed and food law, animal health and animal welfare

rules. According to 882/2004, which sets the legal framework for sampling and analysis methods of feed for control purposes, official controls shall be carried out at any stage of production, processing and distribution of feed, and without prior warning, except in cases such as audits where prior notification of the feed or food business operator is necessary [25].

In accordance with 882/2004 [25], Commission Regulation (EC) No 691/2013 lays down the methods of sampling and analysis for the official control of feed, including methods for the determination of undesirable substances and the constituents of animal origin, methods of analysis of the composition of feed materials and compound feed, as well as methods of control of the level of authorised additives [26].

3 Implications of the EU legislation on the commercialisation of ALG-AD technology

In relation to the specific outputs of the ALG-AD project, the algal biomass growing on waste nutrients (microalgae) are non-GMO and will not be used in feed additive applications. Therefore, the requirements of regulations 1829/2003 (on genetically modified food and feed) and 1831/2003 (on

additives for use in animal nutrition) do not apply, which simplifies the process of commercialisation from a regulatory perspective. However, there are still challenges to be overcome, as well as a number of distinct steps for the successful introduction of ALG-AD technology and the resultant products to the market.

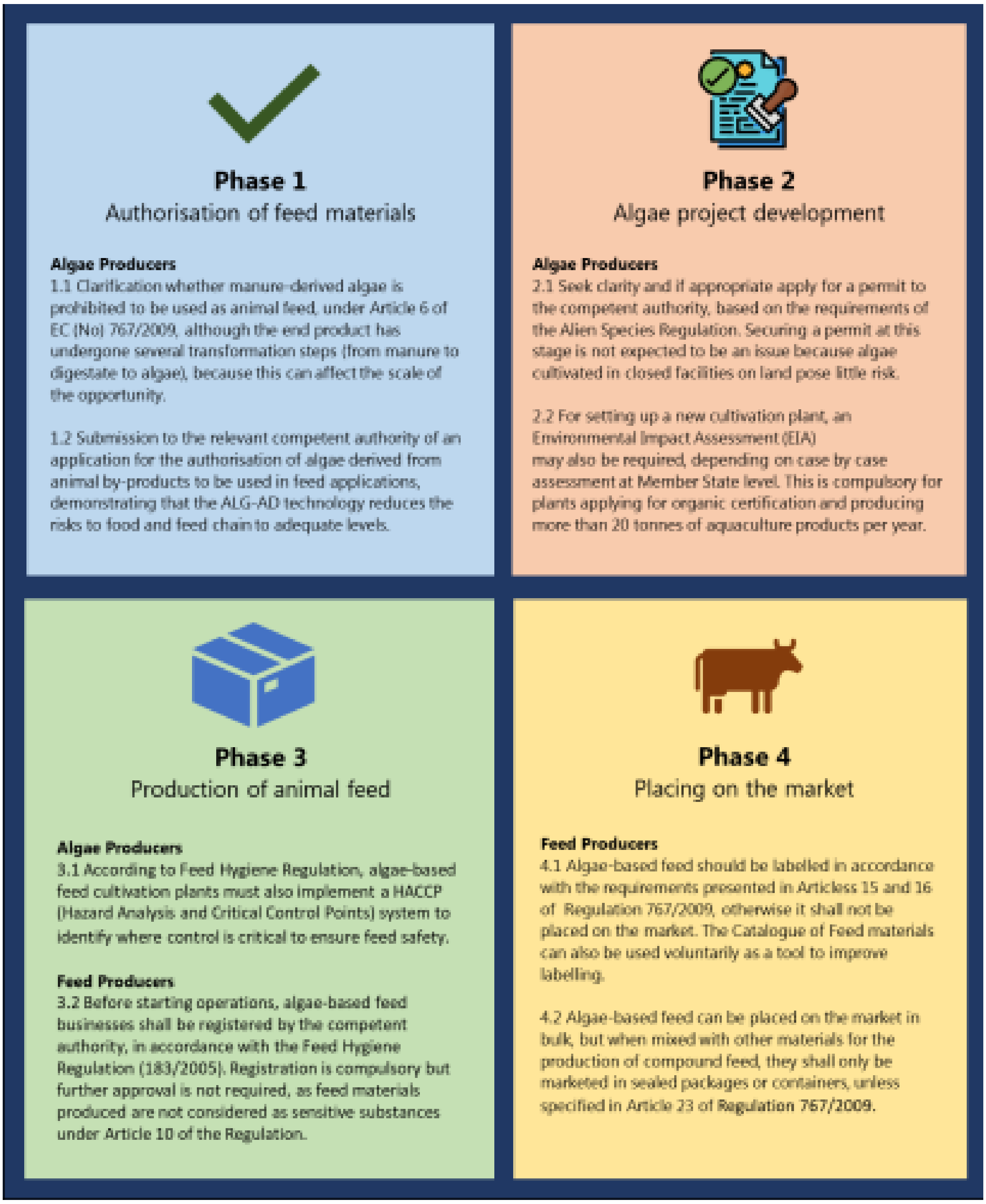

Figure 1: Steps required for ALG-AD operators to ensure compliance with the EU legislation.

CONFIDENTIAL

Figure 1 summarises the steps required for algae producers and feed business operators to ensure compliance with the regulations, while subsequent sections present and discuss issues in more detail.

3.1 Key regulatory issues affecting the introduction of microalgae to the feed market

The main regulatory issue affecting the commercialisation of the ALG-AD technology relates to the provenance of the digestate on which the algae is grown. Digestate generated in AD facilities that use either food waste that contain certain animal by-product types or manure as feedstock, or derivatives thereof, is currently prohibited to be used as feed for animals intended for human consumption and as pet food under **EU Regulation 1069/2009**[28]. This is a barrier that limits the market opportunity, considering that manure and food waste are the most common feedstocks in AD in Europe and used by many current operators. According to Article 18 of the Regulation 1069/2009, by way of derogation, competent authorities can authorise the use of microalgae for special feeding purposes to animals not intended for consumption, such as zoo animals, circus animals, dogs and cats in shelters, fur animals, and wild animals.

ALG-AD technology developers can submit an application to the relevant competent authority (see Appendix 1) requesting the authorisation of microalgae derived from animal by-products to be used in feed applications, demonstrating that the ALG-AD technology reduces risks to the food and feed chain to adequate levels. However, there is always the chance that the application, which will also be reviewed by EFSA, could be rejected and this is likely to be the case where it is not possible to demonstrate appropriate evidence of risk reduction. Guidance on the format for applications for new alternative methods for animal by-products has been published by EFSA [13], providing technical assistance on expected contents and appropriate evidence.

No authorisation is required in the case of animal feed derived from digestate of solely plant origin (e.g. agricultural crops, crop waste and vegetable processing residues), as part of the Regulation 1069/2009. However, any mixture with non-compliant feedstock will be subject to the strictest measures and will require pre-market authorisation.

Apart from any requirements related to the use of nutrients (digestate) of animal origin, no further authorisation is required for using the microalgae developed as animal feed in the EU, as it is not genetically modified, and the European Union has no system of premarket authorisation for feed materials. However, **(EC) No 767/2009** addresses the placing on the market and use of feed within the European Community, and all its requirements must be met.

According to this regulation, “feed shall not contain or consist of materials whose placing on the market or use for animal nutritional purposes is restricted or prohibited”. The list of these feed materials is set out in Annex III of the regulation and includes, among other things commonly used as feedstock in AD, faeces and urine (manure)[29] irrespective of any form of treatment or admixture. Therefore, Regulation (EC) No 767/2009 may prohibit the use of digestate despite the end-product having undergone several transformation steps (from manure to digestate to microalgae).

[28] This statement assumes that an endpoint in the manufacturing chain cannot be defined for products intended for use in animal feed market as explained in section 2.3.

[29] According to Regulation 1069/2009, manure means any excrement and/or urine of farmed animals other than farmed fish, with or without litter.

Further clarification is required by authorities of Member States designated to carry out official controls for the verification of compliance with feed law, as this can have a significant impact on the introduction of the ALG-AD technology on the market.

3.2 Additional steps required for the roll-out of the ALG-AD technology

Under **EU Regulation 708/2007**, microalgae developed as part of the project could be regarded as alien species, and therefore aquaculture operators would be required to apply for a permit; this should be verified by the competent authority as a priority.

Whilst microalgae produced in open ponds would be classed a higher risk movement, resulting in an EIA being required, provisions appear to be less stringent for microalgae cultivated in closed facilities, like the ones developed as part of the project, or whose movement poses no risk to the environment by transferring non-target species. As such, ALG-AD developments are not subject to a prior environmental risk assessment and securing a permit by the competent authority is not expected to be an issue. However, an Environmental Impact Assessment based on the requirements of **2011/92/EU** may be needed, on a case by case assessment of each Member State, while it will be compulsory in the case of farms that wish to obtain organic certification and at the same time intend to produce more than 20 tonnes of feed per year, which can be considered as a low threshold.

Before starting operations, microalgae-based feed business operators shall also be registered by the competent authority, in accordance with the **Feed Hygiene Regulation (183/2005)**, while they must also implement a HACCP (Hazard Analysis and Critical Control Points) system to identify where control is critical to ensure feed safety, if ALG-AD activities are not considered as "primary production" as explained in section 2.4.2.

In addition, microalgae for use as animal feed must be safe with no direct adverse effect on the environment, and at the same time genuine, fit for its purpose, and of merchantable quality, in order to ensure compliance with **Regulation 767/2009**. To guarantee the safety and quality required, including the presence of undesirable substances below maximum levels, safety and nutritional value assessments will be carried out as part of the ALG-AD project. This is of utmost importance, as feed produced will be subject to official controls, which will verify compliance with the EU rules, including safety and quality issues.

Finally, it should be noted that some non-specific algae (processed, live, or dried) are already listed in the Catalogue of Feed Materials. This means that operators might not need to register their product, in accordance with article 24(6) of Regulation 767/2009, when placing them on the market for the first time. As the Catalogue of Feed Materials is a "positive list", it is possible, given advances is science and technology, to apply for the addition of new materials, assuming sufficient evidence is available of the safety of their production and use. Microalgae should not be placed on the animal feed market unless they are properly labelled and packaged in accordance with the requirements of the **Regulation 767/2009**[30].

[30] Microalgae-based feed can be placed on the market in bulk (unless it is differently specified in in Article 23 of the Regulation 767/2009), while the Catalogue of Feed materials can be used voluntary as a tool to improve labelling. However, feed material names listed in the catalogue can only be used when all the provisions set by the Catalogue are fulfilled.

3.3 Key regulatory issues affecting the organic certification of microalgae

Organic certification can potentially determine the scale of the opportunity for microalgae-based products developed as part of the project, but significant amendments to the legislation might be

required for technology users to be able to utilise it.

The main barrier is that **EU Regulation (2018/848)** allows only the use of nutrients of plant or mineral origin for land-based microalgae cultivation systems, otherwise the end-product (on this occasion feed) does not comply with the rules of organic production and cannot be labelled accordingly. The use of animal by-products (e.g. manure or food waste) as a source of nutrients for microalgae cultivation is therefore restricted in organic production, meaning that digestate is not an eligible feedstock unless it is produced exclusively from non-waste plant materials, such as agricultural crops and residues (examples of such feedstocks include: maize, grass, wholecrop cereal silage, outgrade vegetables derived in-field or on-farm, or residues derived in vegetable processing facilities).

It should be noted that, even if it is of plant origin, digestate still requires authorisation so that it can be included in the restrictive list of materials (external inputs) that can be used in organic production systems. However, as the regulation will only apply from January 2021, these lists do not yet exist. Seemingly, consideration of whether a product or substance should be added to the lists shall occur at Member State level. The Member State that wishes to authorise an external input material shall ensure that a dossier giving the reasons for the inclusion is issued to the Commission and to the other Member States. The Commission may then authorise the use of this external input in organic production and adopt an implementing act.

All the above indicate that it will be highly unlikely, under the existing legislation, that feed materials produced using the ALG-AD technology shall be labelled as organic since the current rules appear to be impracticable for producers.

4 Conclusions

This study examines EU legislation relevant to the microalgae production value chain, focusing on the

rules that apply to microalgae for use as feed material, as accessing different markets would possibly require significant changes in the production process.

Generally, microalgae and macroalgae fall within the same legislation when destined for animal feed production, although specific requirements sometimes differ on a case by case basis. Animal feed legislation is typically common across both types of algae as the emphasis is put on the end-product, the production process and the operation of production facilities. Similarly, relevant legislation on animal by-products and derived products focus on the origin of feed materials, rather than their conversion to algae. Other legislation focuses on aquaculture and aquatic organisms in general, without using the term ‘algae’ and as such does not differentiate between microalgae and macroalgae.

Microalgae developed are non-GMO and will not be used in feed additive applications, which simplifies the process of commercialisation, from a regulatory perspective. However, significant challenges need to be overcome for the successful commercialisation of the ALG-AD technology. The main issue appears to be around the use of digestate derived from feedstock containing animal by products that are currently prohibited to enter the feed chain, in accordance with the EU Regulation 1069/2009, even if they have undergone several processing and transformation steps for the production of products with different composition or properties.

This is a barrier that can significantly affect the commercialisation opportunities for the ALG-AD technology, as the use of common feedstock in anaerobic digestion, such as Category 1 and 2 food supply chain waste, for the production of animal feed is currently not allowed; table 2 summarises AD feedstocks permitted for feed production for different animal categories, with some uncertainties still requiring further and specific investigation (marked in orange). As a result, it might be challenging to integrate the ALG-AD equipment to AD facilities that do not exclusively process feedstock of plant origin or certain category 3 animal by-products (excluding catering waste); any mixture with non compliant feedstock will be subject to the strictest measures and will require pre-market authorisation.

Table 2. Summary of AD feedstocks permitted for feed production for different animal categories.

	Farmed	Zoo	Circus	Dogs/cats	Fur	Wild
**Food waste: Cat 2 ABPs**	X	X*	X*	X*	X*	X*
**Food waste: Cat 3 ABPs (incl. catering waste)**	X	X*	X*	X*	X*	X*
**Food waste: Cat 3 ABPs (excl. catering waste)**	✓	✓	✓	✓	✓	✓
**Manure/slurry**	X**	X**	X**	X**	X**	X**

**Plant material (e.g. crops/veg waste)**	✓	✓	✓	✓	✓	✓

* The competent authority may, by way of derogation from Articles 13 and 14, authorise, under conditions which ensure the control of risks to public and animal health, the collection and use of Category 2 material.

** May be prohibited under Regulation 767/2009 which states '…the use of feed containing or consisting of manure is prohibited under (EC) No 767/2009, irrespective of any form of treatment or admixture'. Requires confirmation with the competent authority.

This is an obstacle that can be overcome; Regulation 1069/2009 defines an authorisation procedure for new methods of use and disposal of animal by-products that ensure the safety of the feed chain and further guidance has been released by EFSA in the form of a statement on technical assistance for new applications [13]. As such, ALG-AD developers need to apply to the relevant competent authority (listed in Appendix 1) requesting the authorisation of microalgae derived from animal by-products (incl. any non-plant material) to be used in feed applications, demonstrating that the ALG-AD technology reduces the risks to the food and feed chain to adequate levels. The competent authority, considering the opinion of EFSA, will decide whether authorisation of the ALG-AD method will be granted.

Another important aspect of the EU legislation is that the use of feed containing or consisting of manure is prohibited under (EC) No 767/2009, irrespective of any form of treatment or admixture. Therefore, ALG-AD partners need to confirm the validity of this statement with policy makers and/or competent authorities, as it seems that there is no definite procedure of approval of using manure derived microalgae on the animal feed market and this might affect the scale of the opportunity. Please note that, although this prohibition seems not applicable to other common AD feedstock, such as food waste, a way must be found, if required, to exclude any packaging, from the use of products from the agri-food industry, as their presence in the feed supply chain is prohibited under the same regulation.

Achieving additional requirements for permitting of plants and placing of microalgae-based feed on the market can be considered easier, but still the correct efforts need to be made to ensure compliance with the rules. Lastly, when it comes to organic certification, significant amendments in the legislation might be required for technology users to be able to utilise this opportunity. The main barrier is that the EU Regulation on fertilising products (2018/848) allows only the use of nutrients of plant or mineral origin for land-based microalgae cultivation systems, and therefore excluding the use of feedstock containing animal by-products. Currently, a procedure of authorisation of nutrients of animal origin is not available in organic production, and therefore there is no way to be included in the restrictive list of external inputs allowed to be used in organic production.

Table 3 summarises the relevant legislation that should be considered and may apply directly or indirectly to the ALG-AD value chain. Specific implications and actions are also noted, to inform subsequent activities by ALG-AD partners and future technology and end-product developers.

Table 3. Summary of policies and regulations considered directly or indirectly applicable to ALG-AD

**Legislation applicable to the ALG-AD value chain**		
**Regulation on fertilising products – (EC) 2019/1009**	Indirectly relevant	Lays down provision for the production and supply of fertilising products, including digestate. Defines Product Function Categories and Component Material Categories with positive lists for inputs and methods of production. ALG AD could adopt similar principles for safe production & use.
**Regulations applicable to microalgae production**		
**Common Fisheries Policy**	Potentially supportive	Policy to encourage collaboration, to help increase the sector's production and competitiveness. Strategic guidelines on aquaculture are being revised at Member State level; detail to be reviewed when released as may post opportunities for ALG-AD.
**Alien species legislation – (EC)708/2007**	Requires confirmation	As microalgae will be cultivated outside their known natural range, albeit in closed reactors, it is envisaged that the requirements of (EC) No 708/2007 may apply to the ALG-AD value chains and developers should discuss individual cases with the competent authority of the receiving Member State and apply for a permit if necessary.
**Environmental Impact Assessment – (EC) 2011/92**	Directly relevant; dependent on scale of operation.	Aquaculture projects are listed in Annex II; nation authorities have to decide whether an EIA is neede regardless of the nature or type of cultivation system. A EIA

		is compulsory for any new operators applying for organic production and producing more than 20 tonnes of aquaculture products per year.
**Production and labelling of organic products – (EC) 2018/848**	Directly relevant, in situations where organic status is desired.	Organic certification and labelling are possible, although a transition period will apply throughout which organic-status rules will apply, but organic-status will not be achieved until this period is complete. Guidelines should be assessed early to determine requirements and timeframe for certification.

**Regulation on animal by products and derived-products – (EC) 1069/2009**	Directly relevant.	Prohibits the use of microalgae derived from digestate generated in AD facilities which use food waste that contain animal by-products or manure as feedstock, for use as feed for animals intended for human consumptio and as pet food. By way of derogation, competent authorities can authorise the use of microalgae for specia feeding purposes to animals not intended for consumption, such as zoo animals, circus animals, dogs and cats in shelters, fur animals, and wild animals. Digestate generated from solely plant based feedstocks can also be used, where no animal-derived materials hav been used.
**Regulations applicable to microalgae-based feed applications**		
**Placing on the market and use of feed – (EC) 767/2009**	Directly relevant.	This regulation restricts or prohibits the placing on the market of feed containing certain materials, as listed in Annex III of the regulation. It includes, among other thing commonly used as feedstock in AD, faeces and urine (manure)[31] irrespective of any form of treatment or

[31] According to Regulation 1069/2009, manure means any excrement and/or urine of farmed animals other than farmed fish, with or without litter.

		admixture. This may, therefore, prohibit the use of digestate despite the end-product having undergone several transformation steps (from manure to digestate to microalgae). Further clarification is required.
**Feed hygiene – (EC) 183/2005**	Directly relevant to feed businesses, manufacturing or supplying feed products.	Feed business operators must implement a HACCP system to identify where control is critical to ensure feed safety; feed businesses must be registered and approve by the competent authority and Good Practice Guides should be produced at Member State level.
**Undesirable substances in animal feed – Directive 2002/32/EC**	Directly relevant.	Regulates maximum levels of undesirable substances in products intended for animal feed. Limits are subject to interpretation, dependent on whether microalgae are considered aquatic animals, organisms or plant material under the circumstances in which they are produced. Specific limits should be confirmed with EFSA and the relevant national assurance body in each Member State.

5 References

[1] European Parliament and Council, “REGULATION (EU) 2019/1009 laying down rules on the making available on the market of EU fertilising products.”

[2] European Parliament and Council, “REGULATION (EU) 2016/2031 on protective measures against pests of plants.” [Online]. Available: https://eur-lex.europa.eu/legal content/EN/TXT/?uri=CELEX%3A32016R2031.

[3] European Parliament and Council, “DIRECTIVE 2008/98/EC on waste.”

[4] European Parliament and Council, “REGULATION (EC) No 1069/2009 laying down health rules as regards animal by-products and derived products not intended for human consumption.”

[5] F. Leinemann and V. Mabilia, “European Union Legislation and Policies Relevant for Algae,” 2019. [Online]. Available: https://link.springer.com/chapter/10.1007/978-3-030-25233-5_16.

[6] European Parliament and Council, “DIRECTIVE 2009/41/EC on the contained use of genetically modified micro-organisms.” [Online]. Available: https://eur-lex.europa.eu/legal content/EN/TXT/?uri=CELEX%3A32009L0041.

[7] European Parliament and Council, “DIRECTIVE 2001/18/EC the deliberate release into the environment of genetically modified organisms.”

[8] European Parliament and Council, “DIRECTIVE 2014/89/EU establishing a framework for maritime spatial planning.”

[9] European Parliament and Council, “REGULATION (EC) No 708/2007 concerning use of alien and locally absent species in aquaculture.”

[10] European Commission, “DIRECTIVE 2011/92/EU on the assessment of the effects of certain public and private projects on the environment.” [Online]. Available: https://eur lex.europa.eu/legal-content/EN/TXT/?uri=CELEX%3A32011L0092.

[11] European Council, “Council Regulation (EC) No 834/2007 on organic production and labelling of organic products.”

[12] European Parliament and Council, “REGULATION (EU) 2018/848 on organic production and labelling of organic products.” [Online]. Available: https://eur-lex.europa.eu/legal content/EN/TXT/?uri=uriserv%3AOJ.L_.2018.150.01.0001.01.ENG.

[13] European Food Safety Authority (EFSA), “Statement on technical assistance on the format for applications for new alternative methods for animal by-products,” 2010. [Online]. Available: http://www.efsa.europa.eu/en/efsajournal/pub/1680.

[14] European Parliament and Council, “REGULATION (EC) No 178/2002 laying down the general principles and requirements of food law, establishing the European Food SafetyAuthorityand laying down procedures in matters of food safety.” [Online]. Available: https://eur lex.europa.eu/legal-content/EN/ALL/?uri=CELEX%3A32002R0178.

[15] European Commission, “COMMISSION RECOMMENDATION establishing guidelines for the distinction between feed materials, feed additives, biocidal products and veterinary medicinal products.” [Online]. Available: https://eur-lex.europa.eu/legal content/EN/TXT/PDF/?uri=CELEX:32011H0025&from=GA.

[16] European Parliament and Council, “REGULATION (EC) No 1831/2003 on additives for use in

animal nutrition.”

[17] European Parliament and Council, “REGULATION (EC) No 1829/2003 on genetically modified food and feed.”

[18] O. Smedley, “Comparison of Regulatory Management of Authorized Ingredients, Approval Processes, and Risk-Assessment Procedures for Feed Ingredients,” Int. Feed Ind. Fed., 2013.

[19] European Parliament and Council, “REGULATION (EC) No 767/2009 on the placing on the market and use of feed.” [Online]. Available: https://eur-lex.europa.eu/legal content/EN/ALL/?uri=CELEX%3A32009R0767.

[20] European Commission, “COMMISSION REGULATION (EU) No 68/2013 on the Catalogue of feed materials.” [Online]. Available: https://eur lex.europa.eu/LexUriServ/LexUriServ.do?uri=OJ:L:2013:029:0001:0064:EN:PDF.

[21] European Parliament and Council, “REGULATION (EC) No 183/2005 laying down requirements for feed hygiene.”

[22] European Commission, “Feed Hygiene: Guides to Good Practice.” [Online]. Available: https://ec.europa.eu/food/safety/animal-feed/feed-hygiene/guides-good-practice_en.

[23] European Parliament and Council, “DIRECTIVE 2002/32/EC on undesirable substances in animal feed.”

[24] European Parliament and Council, “REGULATION (EC) No 882/2004 on official controls performed to ensure the verification of compliance with feed and food law, animal health and animal welfare rules.”

[25] European Parliament and Council, “COMMISSION REGULATION (EU) No 691/2013 on methods of sampling and analysis of animal feed.”

[26] European Commission, “Legislation on feed additives.” [Online]. Available: https://ec.europa.eu/food/safety/animal-feed/feed-additives/legislation-feed-additives_en.

[27] Agricultural Industries Confederation (AIC), “Animal Feed Legislation and Guidance.” [28] UK

government, “The Alien and Locally Absent Species in Aquaculture (England and Wales).”

[29] Ministere de l’agriculture et alimentation (France), “Use of exotic and locally absent species in aquaculture.” [Online]. Available: https://agriculture.gouv.fr/utilisation-en-aquaculture despeces-exotiques-et-localement-absentes. [Accessed: 28-Apr-2020].

[30] Belgian Federal Public Service, “Use of alien and locally absent species.” [Online]. Available: https://healthpr.belgium.be/en/animals-and-plants/biodiversity/invasive-alien-species/belgian national-aquaculture-portal-site. [Accessed: 28-Apr-2020].

[31] European Commission, “Guidance document on the implementation of certain provisions of Regulation (EC) No 183/2005 laying down requirements for feed hygiene (C/2019/4919),” 2019. [Online]. Available: https://eur-lex.europa.eu/legal content/EN/TXT/?uri=uriserv:OJ.C_.2019.225.01.0001.01.ENG. [Accessed: 29-Apr-2020].

6 Appendices

6.1 Appendix 1: List of Competent Authorities

EFSA does not provide a list of competent authorities as referred to throughout regulation 1069/2009. It does, however, provide a list of competent organisations with which it works. From this list, those organisations that would be considered “authorities” under common understanding of this term are listed here, where their expertise, according to the EFSA, covers “Products/Substances in Animal Feed”. Where multiple such authorities exist, all are listed.

Countries	Competent Authorities
United Kingdom	Department for Environment, Food and Rural Affairs<br>Nobel House<br>17 Smith Square<br>London<br>SW1P 3JR<br>https://www.gov.uk/government/organisations/department-for-environment food-rural-affairs
	Food Standards Agency<br>Floors 6 & 7<br>Clive House<br>70 Petty France<br>London<br>SW1H 9EX<br>https://www.food.gov.uk/
Ireland	Department of Agriculture, Food and the Marine<br>Kildare Street<br>Dublin 2<br>D02 WK12<br>https://www.agriculture.gov.ie/
	Food Safety Authority of Ireland<br>The Exchange<br>George’s Dock<br>IFSC<br>Dublin 1<br>D012 P2V6<br>https://www.fsai.ie/

Germany	Bundesamt für Verbraucherschutz und Lebensmittelsicherheit<br>Bundesallee 35<br>Braunschweig<br>38116<br>https://www.bvl.bund.de/

	Bundesinstitut für Risikobewertung<br>Max-Dohrn-Straße 8-10<br>Berlin<br>10589<br>https://www.bfr.bund.de/en/home.html
France	Agence nationale de sécurité sanitaire de l'alimentation<br>14 rue Pierre et Marie Curie<br>Maisons-Alfort<br>94701<br>https://www.anses.fr/
Belgium	Service public fédéral - Santé, Sécurité alimentaire et Environnement Victor Horta Place 40 box 10<br>Brussels<br>1060<br>https://www.health.belgium.be/
	Agence fédérale pour la sécurité de la chaîne alimentaire<br>Boulevard du Jardin Botanique 55<br>Brussels<br>1000<br>http://www.favv-afsca.be/

6.2 Appendix 2: Procedure for the authorisation of an alternative method

Article 20 of the (EU) 1069/2009

Authorisation of alternative methods

The procedure for authorisation of an alternative method of use or disposal of animal by-products or derived products may be initiated either by the Commission or, following an application, by a Member State or by an interested party, which may represent several interested parties.

1) Interested parties shall send their applications to the competent authority of the Member State where they intend to use the alternative method. The competent authority shall evaluate, within a period of two months following receipt of a complete application, whether the application

complies with the standard format for applications referred to in point 9.

2) The competent authority shall communicate the applications of the Member States and interested parties, together with a report on its evaluation to the European Food Safety Authority (EFSA) and inform the Commission thereof.

3) When the Commission initiates the procedure for authorisation, it shall send a report on its evaluation to EFSA.

4) EFSA shall assess, within six months following receipt of a complete application, whether the method submitted ensures that risks to public or animal health are: (a) controlled in a manner which prevents their proliferation before disposal in accordance with this Regulation or the implementing measures thereof; or (b) reduced to a degree which is at least equivalent, for the relevant category of animal by-products, to the processing methods laid down pursuant to point (b) of the first subparagraph of Article 15(1) of the Regulation. EFSA shall issue an opinion on the application submitted.

5) In duly justified cases where EFSA requests additional information from applicants, the period provided for in point 4 may be extended. After consulting the Commission or the applicant, EFSA shall decide on a period within which that information shall be provided to it and inform the Commission and the applicant as appropriate of the additional period needed.

6) Where applicants wish to submit additional information on their own initiative, they shall send it directly to EFSA. In that case the period provided for in point 4 shall not be extended by an additional period.

7) EFSA shall forward its opinion to the Commission, the applicant and the competent authority of the Member State concerned.

8) Within three months following receipt of the opinion of EFSA and taking account of that opinion, the Commission shall inform the applicant of the proposed measure to be adopted in accordance with point 10.

9) A standard format for applications for alternative methods shall be adopted in accordance with the advisory procedure referred to in Article 52(2) of the Regulation.

10) Following receipt of the opinion of EFSA, the following shall be adopted: (a) either a measure authorising an alternative method of use or disposal of animal by-products or derived products; or (b) a measure rejecting the authorisation of such an alternative method.

6.3 Appendix 3: Guidelines for the distinction of different types of feed

'Feed materials': products of vegetable or animal origin, or derivatives thereof, whose principal purpose is to meet animals' nutritional needs, in their natural state, fresh or preserved, and products derived from the industrial processing thereof, and organic or inorganic substances, whether or not containing feed additives, which are intended for use in oral animal-feeding either directly as such, or after processing, or in the preparation of compound feed, or as carrier of premixtures.

Feed materials are primarily used to meet animals' needs, for example for energy, nutrients, minerals or dietary fibres, and they are usually not chemically well-defined except for basic nutritional constituents.

'Feed additives': substances, micro-organisms or preparations, other than feed material and premixtures, which are intentionally added to feed or water in order to perform, in particular, one or

more specific functions that are enumerated in Article 5(3) of the Regulation (EC) No 1831/2003:

(a) favourably affect the characteristics of feed,
(b) favourably affect the characteristics of animal products; Compound feed including pet food
(c) favourably affect the colour of ornamental fish and birds,
(d) satisfy the nutritional needs of animals,
(e) favourably affect the environmental consequences of animal production,
(f) favourably affect animal production, performance or welfare, particularly by affecting the gastro-intestinal flora or digestibility of feedingstuffs, or
(g) have a coccidiostatic or histomonostatic effect. It should be noted that these functions are not exclusive to feed additives, as a feed material can also exert an additive function, but this should not be the only intended use. However, a product cannot be at the same a feed material and a feed additive.

Additives may be classified into the following categories [27]: technological additives (e.g. preservatives, antioxidants, emulsifiers, stabilising agents, acidity regulators, silage additives), sensory additives (e.g. flavourings, colorants), nutritional additives (e.g. vitamins, minerals, aminoacids, trace elements), zootechnical additives (e.g. digestibility enhancers, gut flora stabilizers), and Coccidiostats and histomonostats.

‘**Biocidal products’**: active substances and preparations containing one or more active substances, put up in the form in which they are supplied to the user, intended to destroy, deter, render harmless, prevent the action of, or otherwise exert a controlling effect on any harmful organism by chemical or biological means.

‘**Veterinary medicinal product’**: any substance or combination of substances presented as having properties for treating or preventing disease in animals, or any substance or combination of substances which may be used in or administered to animals with a view either to restoring, correcting or modifying physiological functions by exerting a pharmacological, immunological or metabolic action, or to making a medical diagnosis.
CONFIDENTIAL

6.4 Appendix 4: Mandatory labelling requirements for feed materials

Article 15 of the (EU) 767/2009

General Mandatory Labelling requirements

A feed material or compound feed shall not be placed on the market unless the following particulars are indicated by labelling:

1. the type of feed: ‘feed material’, ‘complete feed’ or ‘complementary feed’, as appropriate. 2. the name or business name and the address of the feed business operator responsible for the labelling.
3. If available, the establishment approval number of the person responsible for the labelling granted in accordance with Article 13 of Regulation (EC) No 1774/2002 for establishments authorised in accordance with Article 23(2)(a), (b), and (c) of Regulation (EC) No 1774/2002 or Article 17 of Regulation (EC) No 1774/2002 or with Article 10 of Regulation (EC) No 183/2005. If a person responsible for the labelling has several approval numbers, they shall use the one granted in accordance with Regulation (EC) No 183/2005,
4. The batch or lot reference number.

5. The net quantity expressed in units of mass in the case of solid products, and in units of mass or volume in the case of liquid products.
6. The list of feed additives preceded by the heading ‘additives’ in accordance with Chapter I of Annex VI or VII, as applicable, and without prejudice to labelling provisions laid down in the legal act authorising the respective feed additive.
7. The moisture content in accordance with point 6 of Annex I of the Regulation.

Article 16 of the (EU) 767/2009

Specific mandatory labelling requirements for feed materials

In addition to the General Mandatory Labelling requirements, presented above, the labelling of feed materials shall also include:

1. The name of the feed material; the name shall be used in compliance with Article 24(5). 2. The compulsory declaration corresponding to the respective category as set out in the list in Annex V of the Regulation 767/2009; the compulsory declaration may be replaced by the particulars laid down in the Community Catalogue referred to in Article 24 for each feed material in the respective category. 2.

The labelling of feed materials should also include the following particulars, when additives are incorporated:

1. the species or categories of animals for which the feed material is intended where the additives in question have not been authorised for all animal species or have been authorised with maximum limits for some species.
2. Instructions for proper use in accordance with point 4 of Annex II of the Regulation 767/2009, where a maximum content of the additives in question is set.
3. The minimum storage life for additives other than technological additives.

NNFCC is a leading international consultancy with expertise on the conversion of biomass to [illegible] d products.

NNFCC, Biocentre,
York Science Park,
Innovation Way,
Heslington, York,
YO10 5NY.

Phone: +44 (0)1904 435182
Fax: +44 (0)1904 435345
E: enquiries@nnfcc.co.uk
Web: www.nnfcc.co.uk
