# Supplementary material for: Scale-Up to Pilot of a Non-Axenic Culture of Thraustochytrids Using Digestate from Methanization as Nitrogen Source
Source: Mar Drugs. 2022 Aug 2;20(8):499. doi: 10.3390/md20080499 (PMC9410245; doi:10.3390/md20080499)
Supplement: Supplementary file 1 [file marinedrugs-20-00499-s001.zip › marinedrugs-1809880-reports/Interreg NWE ALG-AD project Safety analysis digestate and algal biomass produced by the three ALG-AD pilots.pdf]

Interreg
EUROPEAN UNION
North-West Europe
ALG-AD
European Regional Development Fund

THEMATIC PRIORITY

RESOURCE AND MATERIAL EFFICIENCY

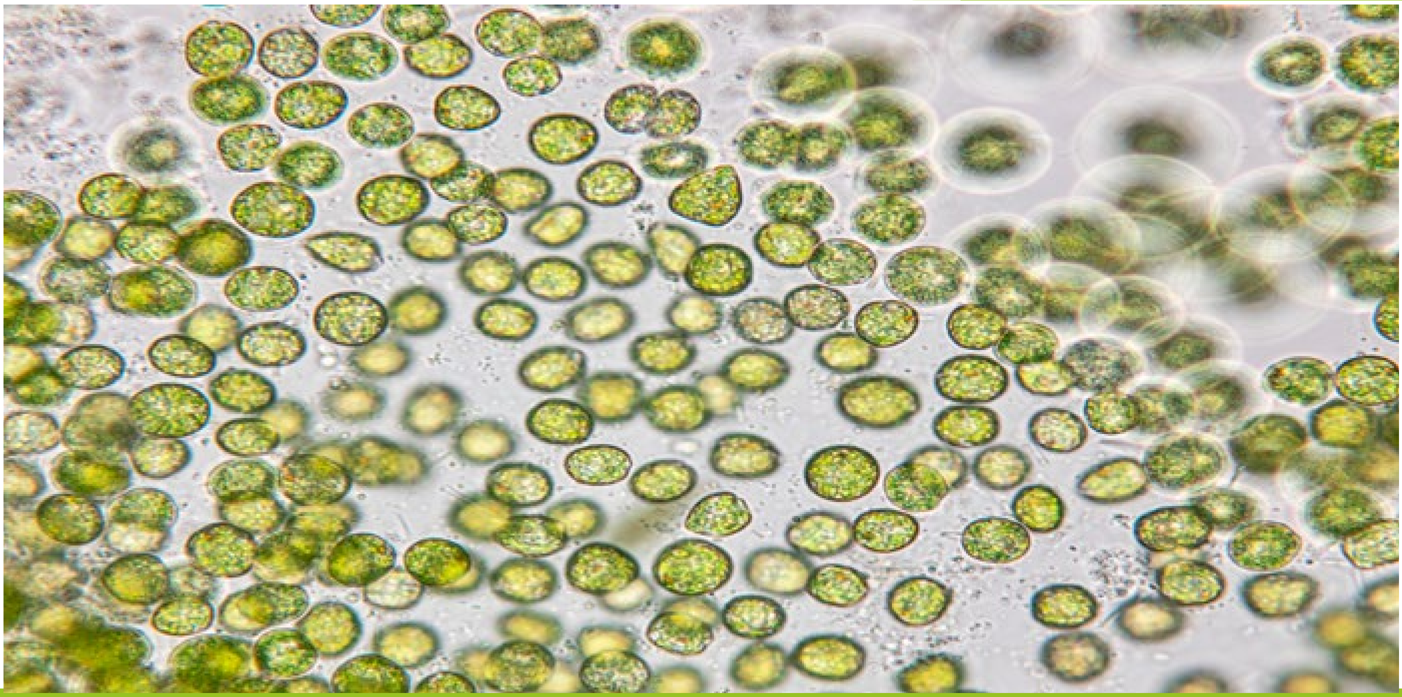

SAFETY ANALYSIS: DIGESTATE AND ALGAL BIOMASS PRODUCED BY THE THREE ALG-AD PILOTS

CONDUCTED BY ANSES, PARTNERS ON THE ALG-AD PROJECT.

anses

EDITION : JANUARY 2022

Corresponding Authors

The HQPAP unit (Martine Denis and Caroline Le Marechal) produced the database and the preliminary report on zoonotic pathogens.

The PBER unit (Virginie Thibault, Lucie Pinault, Marc Tabouret) produced the database for *Mycobacterium paratuberculosis.*

Alassane Keïta is coordinating the ALG-AD project for Anses and produced the final report in coordination with Caroline Le Marechal, Virginie Poisson and Yannick Blanchard..

Contact: **Alassane.Keita@anses.fr**

Please cite this document as follow:

Keita, A, Le Marechal, C, Poisson, V, Blanchard, Y. (2021). O Safety analysis carried out by Anses in the ALG-AD Project. Public Output report of the ALG-AD project, Swansea, November 2021, 19 pages.

Available online at www.nweurope.eu/projects/ALG-AD.

This document is an output from the ALG-AD project, which has received European Regional Development Funding through the INTERREG IVB NWE programme.

@ALG-AD project partnership, 2021, all rights reserved.

Cronfa Datblygu Rhanbarthol Ewrop
European Regional Development Fund

Executive summary

This document is a technical report detailing the methods applied to conduct safety analysis on the digestates and microalgal biomass produced as part of the ALG-AD project. This document is an ouput of the INTERREG North West Europe funded ALG-AD project aiming to combine anaerobic digestion (AD) and microalgal cultivation technologies to remediate nutrient-rich digestate currently produced in excess in the area.

The main objective of this report is to document the methodology and results of the safety analysis conducted on the digestate and biomass produced at the three pilot facilities based at industrial locations in the UK, France and Belgium. The document draws conclusions and recommendations.

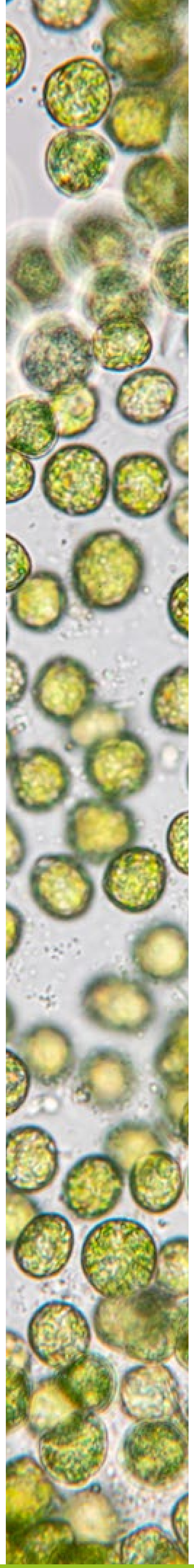

TABLE OF CONTENTS

PROTOCOL FOR OBTAINING DIGESTATES AND CULTURES 4
- Cooperl Digestate origin and processing 4
  - Algae cultivation and harvesting 4
- Langage - Digestate origin and processing 4
  - Algae cultivation and harvesting 4
- Innolab - Digestate origin and processing 4
  - Algae cultivation and harvesting 4

SAFETY ANALYSIS 5
- Background to justify the selection of pathogens 5
- Context 5

METHODS 7
RESULTS 8
- Zoonotic pathogens 8
  - Clostridium botulinum 8
  - Clostridioides difficile 9
- Mycobacterium avium subsp. paratuberculosis 9
- Metagenomic analysis 10
  - Brief introduction to 16S metagenomics 10
  - Material and Methods 10
  - Results 10
  - DNA extraction: 10
  - 16S Metagenomic Analysis 11
  - OTU clustering. 11
  - Rarefaction curves. 11
  - Phylogenetic affiliation of OTU 12

CONCLUSIONS 14
REFERENCES 15
APPENDICES 17
- Appendix 1. Bacteriological analyses at Anses for the ALG-AD project Dec20 17
  1) Detection and enumeration of Listeria monocytogenes: 17
  2) Detection and enumeration of thermotolerant Campylobacter: 17
  3) Detection and enumeration of Salmonella: 17
  4) Detection and enumeration of C. botulinum: 17
  5) Detection and enumeration of Clostridium difficile: 18
  6) Detection of Mycobacterium avium subsp. paratuberculosis 18
- Annex 2.1: BD_result pathogenes Alg-AD_Anses July 2020 19
- Annex 2.2 BD_result pathogenes Alg-AD_Anses Sept 2020 19

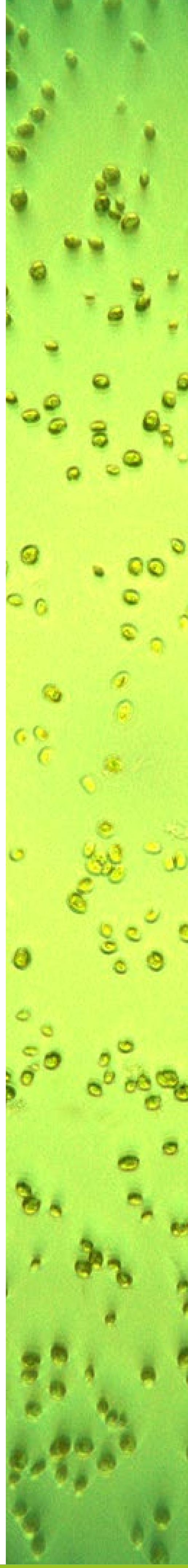

0 PROTOCOL FOR OBTAINING DIGESTATES AND CULTURES

Cooperl Digestate origin and processing

The digester works under mesophilic conditions (38°C) and is fed with solid pig manure, slaughterhouse wastewater treatment sludge and plant sludge. Pig manure is diluted with reverse osmosis treated water from a wastewater treatment plant. The digestate is centrifuged on a centrifugal decanter after polymer addition. After decanting, two phases are obtained: a solid fraction and a liquid fraction. The liquid fraction is provided for the ALG-AD project. The liquid part is then filtered by crossflow filtration (pore size: 300 kDa). Digestate for pathogens analysis is collected at this step.

Algae cultivation and harvesting

The growing medium is composed of the filtered digestate (2.5% V/V) with the addition of sea salt at 15 g/L, glucose syrup at 20 g/L, yeast extract peptone (2g/L) both sterilised by autoclaving and tap water. An inoculum, produced in sterile conditions, is then inoculated at 1/30th. The culture lasts about 2 days with the addition of air bubbles, and the raw culture is then concentrated by crossflow filtration (pore size: 300 kDa) for recovering the algal biomass. Biomass for pathogens analysis is collected at this step.

Langage - Digestate origin and processing

The raw digestate (800 L) comes from the AD tank and is transported in a plastic tank (1000 L). The tank is put on top of a pallet at 1 m above the floor level in order to use gravity to move the raw digestate (thick sludge) from the storage tank to the membrane tank. When the raw digestate is in the membrane tank (200 L), the system starts to operate and the raw digestate passes through the tubular membrane with the help of a pump. This tubular membrane (pore size: 100 KDa) has two outlets: the retentate, in which the concentrated raw digestate is recirculated, and the permeate. The pure filtered digestate (permeate) is then collected to be used in the cultivation phase in the phototrophic PBR.

Algae cultivation and harvesting

Cultivation starts in the phototrophic reactor using 20% (v/v) inoculum (Scenedesmus sp. culture subcultured in bags or carboys) and 80% (v/v) tap water previously filtered, stored with bleach (0.5 mL/L) and neutralized using 0.2 g/L of sodium thiosulphate before inoculation (disinfection process). In steady state mode, 2% (v/v) of digestate is currently used in the PBR as the standard concentration. When the desirable biomass density (0.4 g/L) is reached in the phototrophic PBR (at the beginning after 20 to 25 days from day 0, and then every week), 30% of the phototrophic volume are harvested.

Innolab - Digestate origin and processing

The liquid fraction of digestate (LF) was collected from the AM-POWER BVBA AD plant in Pittem, Belgium, which processes food and farm waste in thermophilic digesters. The AD feedstock primarily includes up to 90% of organic biological waste (industrial food waste and source segregated food waste) and the remainder of energy maize, glycerine and fat-rich substrates. These substrates are homogenized (dry matter content of up to 20%) and hydrolyzed before being fed into the digesters with a retention time of 50-60 days. The digested slurry is then post-digested for 10 days and sanitized at 70° C for 1 h to produce a hygienic digestate. The LF was produced after processing the hygienized digestate using a decanter centrifuge. Later on, the collected LF was pre-treated at ALG-AD Innolab investment site using a 10-µm paper filter bag (Holland Filter, The Netherlands) to produce a clearer liquid free of larger particles.

Algae cultivation and harvesting

The tap water used for the microalgal cultivation was passed through a UV disinfection system (Holland Filter, The Netherlands) and then circulated into the reactor. The paper-filtered liquid fraction of digestate is fed at 2-5% (v/v) and, after recirculation for homogenization, a mixed culture of Chlorella sp. and Desmodesmus sp. is inoculated at 5% (v/v) and grown until reaching the maximum biomass concentration of 1.5 g/L DW (between one and three weeks, depending on weather conditions). For pathogen analysis, a sample was collected from the sampling point of the photobioreactor and harvested by centrifugation.

1 SAFETY ANALYSIS

Background to justify the selection of pathogens

Digestate generated in AD facilities using Category 2 ABPs is currently prohibited to be used for farmed animals feed. The EU regulation 142/2011, Annex V, section 3 (standards for digestion residues and composts), 1b states that "representative samples must comply with the absence of Salmonella in 25g". From these observations, there are currently no regulations on the health criteria applicable to digestates used to grow algae. Therefore, the choice of pathogens that we analyzed was based on the papers currently published to assess the effect of AD on the reduction of pathogens known to be present in manure and relevant from a One Health perspective.

Thus, as part of the ALG-AD project, the HQPAP unit carried out the detection and numeration of 2 sporulating (*Clostridioides difficile* and *Clostridium botulinum*) and 4 non-sporulating pathogens (thermotolerant *Campylobacter*, *Salmonella*, *Yersinia enterocolitica*, *Listeria monocytogenes*) commonly detected in slurry from livestock and that should be considered from a One health perspective. In addition, the PBER unit (pathology and welfare for ruminants) carried out the detection of *Mycobacterium avium subsp. paratuberculosis*. The analysis methods to detect these pathogens have been developed in by our laboratory.

Taking into account the variety of pathogens that we analysed, it can be considered that the number of pathogens analysed is greater than those analysed in the papers currently published. Despite this, it cannot be considered that we have analysed all possible pathogens, which is otherwise unrealistic. This choice of pathogens was also based on the analysis methods available at ANSES.

Context

The presence of pathogenic microorganisms in livestock manure introduced into anaerobic digesters can present a health risk for humans and animals. In fact, under the EU 142/2011 regulation, the spread of the digestate on crops or pastures can lead to the spread of pathogens in the agricultural environment and contaminate farm animals and humans (Froeschle et al., 2015).

Pathogenic bacteria such as *Campylobacter jejuni, Salmonella spp., Yersinia enterocolitica* and *Listeria monocytogenes* are known to be responsible for major food-borne zoonotic diseases (EFSA, 2016). These pathogenic bacteria are also excreted by farm animals (such as pigs and ruminants) that constitute a reservoir of pathogens (Avrain et al., 2004; Boscher et al., 2012; Kempf et al., 2017; Milnes et al., 2008; Patterson et al., 2016; Tadesse et al., 2011; Thépault et al., 2018). Moreover, these pathogens can persist up to few months in manure, soil and water (Cevallos‐Cevallos et al., 2014; Erickson et al., 2014; Jäderlund et al., 2011). Few studies have been conducted to evaluate the fate of these pathogens through anaerobic digestion: a reduction by only 1–2 log units has been reported for pathogens through mesophilic AD under 35°C to 40 °C (Avery et al, 2012). A recent study conducted at ANSES has shown that most of them are still detected in digestates (Le Maréchal et al. 2019). In this study, Salmonella for example was detected in digestates up to 7x102 most probable numbers per gram, when the EU 142/2011 regulation states that digestion residues and composts must comply with the absence of this pathogen in 25 g. Considering the public health issues as far as these pathogens are concerned and their ability to survive through mesophilic AD and storage after AD, monitoring of these major zoonotic pathogens is crucial to evaluate the sanitation efficiency of the AD process.

Besides these pathogens, currently constituting the main zoonotic foodborne agents in Europe, attention should also be paid towards sporulating anaerobic pathogens. They are known to be very resistant to conditions retrieved in AD plants (Bagge et al. 2005, Olsen et al. 1987, Lebuhn et al. 2005, Massé et al. 2011). Among clostridial pathogens, *C. botulinum* that produces the botulinum neurotoxin, which is the most potent toxin, has regularly attracted media attention in connection with AD, particularly in Germany (Fröschle et al. 2015). It was indeed claimed that *C. botulinum* may proliferate in AD and then disseminate through the spreading of digestate on soils, resulting in environment contamination and potential public and animal health

risks (Fröschle et al. 2015, Böhnel and Gessler 2010; Boll 2011). Besides the potential “One Health” issues, monitoring of *C. botulinum* during the AD operation is also crucial to reassure public opinion regarding AD. A second clostridial pathogen has recently been highlighted in AD. *C. difficile* is an emerging pathogen associated with diarrhoea in humans that can result in hospitalisations. The rise in the number of *C. difficile* infections among the general population is worrying and not well understood until now. The zoonotic status of *C. difficile* is highly suspected and environmental contamination is one of the main suspected contamination pathways. To the best of our knowledge, only two studies have been published about the effect of AD on *C. difficile* (Fröschle et al. 2015, Le Maréchal et al. 2019) but they both show that this pathogen survives AD and should be monitored to prevent its emergence through AD or digestate spreading.

Mycobacterium avium subspecies *paratuberculosis* (MAP) is the causative agent of paratuberculosis, a chronic granulomatous enteritis in domestic and non-domestic ruminants. Clinically affected animals present symptoms of wasting and emaciation. However, MAP can also infect non-ruminant animal species with less specific signs. Transmission principally occurs by the faecal-oral route, either through sucking the manure-contaminated teats or, by the uptake of feed contaminated with faeces. *Vertical* transmission also occurs. Whether MAP can be regarded as a potential public health issue and pathogenic in humans (Crohn’s disease) is discussed but still not confirmed (Sartor, 2005).

Infected animals shed MAP in their faeces, leading to the contamination of environment (pastures, soil, water) is resistant to various physical conditions and is known for its ability to survive in the environment for a long time (Whittington, 2004 and 2005).Because of those characteristics, it is essential to assess the presence and survival of MAP in that process.

1 METHODS

The samples were collected from the three Investment sites of the ALG-AD project (Cooperl, Innolab and Langage), which utilized digestate of different origins for microalgal cultivation. At each site, digestate before (D_BF) and after filtration (D_AF), as well as concentrated algal culture after harvesting and partial dewatering (Alg), were collected. Three replicates of each matrix were sampled by the responsible personnel of the site. All the samples were sent as quickly as possible to the laboratory for analysis under refrigerated conditions. It should be noted that the matrices analysed in September 2020 (i.e non-filtered digestate, filtered digestate and algal culture after filtration) and those analysed in February 2020 (filtered digestate, algal culture before and after filtration) were different. Consequently, from the seasonal variation point of view, we will be able to compare the results only for filtered digestate and algae after filtration.

The delivery of Langage samples sent in September 2020 was delayed, which led to a longer time between sampling and analysis than for other sites or when compared to the first analysis conducted in February. This led to the use of media for pathogens analysis for which the validated storage time had been exceeded (non-conformity form: PPN-NC-2020-200, Derogation form for the use of expired media: PPN-DRG-2020-063). This is an internal quality procedure explaining that the analytical media were used 1 day after the date of theoretical use, but it probably did not have any consequences on the results as long as the controls were validated.

The detection of the pathogens was done as described previously (Fondrevez et al., 2010; Le Maréchal et al., 2019). The numeration was done by MPN (Most Probable Number) method using a 12-well microplate for the enrichment in broths. After incubation of the microplate, all the wells were streaked on agar plates in order to detect the growth of the pathogen in each well, except for *C. botulinum*, for which the growth of the pathogen in each well was determined after DNA extraction and PCR. Broths, agar plates, temperatures of incubation, incubation times, and PCR were the same as used for the detection. The estimation of the number of Colony Forming Units/g in each replicate was determined using an MPN calculator.

Mycobacterium avium subsp. paratuberculosis was detected using a qPCR (quantitative PCR). When a positive result was found by the PCR method, it is interesting to put the samples in culture (numeration method) to check whether the bacteria are alive or not.

The detailed bacteriological methods are shown in Annex 1 (Bacteriological analyses at Anses for the ALG-AD project Dec20).

NGS analysis was carried out on digestate before filtration (D_BF), digestate after filtration (D_AF), and algae biomass samples from the three sampling sites.

2 RESULTS

Zoonotic pathogens

All the results are presented in the database BD_result pathogenes Alg-AD_Anses july 2020.xls for the samples sent in February 2020 and database BD_result pathogenes Alg-AD_Anses Sept 2020.xls for those sent in September 2020.

At both the time steps, all the samples, irrespective of the matrix and the sampling site, were negative for *Campylobacter, Listeria monocytogenes, Yersinia enterocolitica and Salmonella.*

Positive results were observed for *Clostridium botulinum* and *Clostridium difficile*. Their detection and numeration are presented in Table I:

Clostridium botulinum

There are currently nine botulinum neurotoxins described in the literature and more than 40 subtypes. Botulinum neurotoxin type A and B are the most common ones involved in human botulism outbreaks while botulinum neurotoxins produced by *C. botulinum GIII* (which are toxins C, D and their mosaic C/D and D/C) are responsible for animal botulism outbreaks.

Here we detect and enumerate *C. botulinum* (either spores or vegetative cells) using a PCR targeting the genes encoding for botulinum toxins after an enrichment step under anaerobic conditions. Only viable forms were detected. No assay was conducted to detect the botulinum neurotoxin.

		Sampling in February 2020				Sampling in September 2020			
		Clostridium botulinum results	Comments	Clostridioides difficile results	Comments	Clostridium botulinum results	Comments	Clostridioides difficile results	Comments
Cooperl	DBF	No sampling				3+/3	enumeration < 1.3 MPN/g; Toxin GIII gene	not detected	
	DAF	not detected		not detected		not detected		not detected	
	Algae	not detected		not detected		not detected		not detected	
Innolab	DBF	No sampling				3+/3	between 1.3 and 1.6 MPN/g; Toxin A, B, GIII genes	3+/3	< 1.3 MPN/g
	DAF	3+/3	3.8 MPN/g, toxin B, GIII<br>6.1 MPN/g, toxin B, GIII<br>13.0 MPN/g, toxin A,B, GIII	1+/3	<1.3 MPN/g	3+/3	< 1.3 MPN/g, Toxin A, B, GIII genes	3+/3	< 1.3 MPN/g
	Algae	only one sample analysed, insufficient quantity of algae<br>1+/1	1.3 MPN/g	not detected		1+/3	1.3 MPN/g; Toxin GIII gene	2+/3	1.4 MPN/g
Langage	DBF	No sampling				3+/3	respectively 15, 64 and 110 MPN/g for GIII gene (mean: 63.0) ; 6.1 - 1.6 - 6.1 MPN/g for Toxin B gene (mean: 4.6)	1+/3	< 1.3 MPN/g
	DAF	not detected		not detected		not detected		not detected	
	Algae	not detected		not detected		not detected		2+/3	between 1.3 and 1.6 MPN/g

DBF = digestate before filtration; DAF = digestate after filtration

MPN: most probable number

Table I: detection and numeration of *Clostridium botulinum* and *Clostridioides difficile* according to sites and types of samples

Samples sent in February 2020

Cooperl and Langage: all samples were negative.

Innolab: all the DAF (Toxin A, B, GIII genes with 7.6 mean MPN/g) and one algae sample (out of one) was positive (1.3 MPN/g).

Samples sent in September 2020

Cooperl: only the DBF samples were positive (3 positive/3; Toxin GIII gene) whereas DAF and algae samples were negative.

Innolab: all the DBF (Toxin A, B, GIII genes) and DAF (Toxin A, B, GIII genes) samples were positive and one algae sample (out of 3, with Toxin GIII gene) was positive . However, the level of contamination was very low (< 1.3 MPN/g for the positive algae sample).

Langage: All the DBF samples were positive at a high level for Toxin GIII gene (mean 63.0 MPN. g^{-1}) and for Toxin B gene (mean 4.6 MPN. g^{-1}). In contrast, all DAF and algae samples were negative.

Clostridioides difficile

The method used in this study to detect *C. difficile* targeted both spores and vegetative cells. No assay was conducted to detect toxins in the samples. The infectious dose and mechanisms behind the initiation of the infection in humans or animals are unkown.

Samples sent in February 2020

Cooperl and Langage: all samples were negative.

Innolab: One DAF sample (out of 3) was found positive and all algae samples were negative.

Samples sent in September 2020

Cooperl: all the DBF and DAF samples were negative. Surprisingly, one algae sample was positive (3.8 MPN/g).

Innolab: all the DBF and DAF samples were positive and two algae samples (out of 3) were positive. However, the level of contamination was very low (maximum of 1.4 MPN/g).

Langage: One DBF sample was positive, all the DAF samples were negative and two algae samples were positive. However, the level of contamination was very low (maximum of 1.6 MPN/g).

Mycobacterium avium subsp. paratuberculosis

Results are presented in Tc (Threshold cycles) in the Table II. A PCR has about 40 cycles. The higher the value of Tc (30-35), the fewer genes expressed. On contrary, the lower the value of Tc (10-15), the more genes expressed.

Samples sent in February 2020

All the results are negative for Langage and Cooperl. For Innolab, all the results were positive. However, the results show Tc values greater than 33, which means

	Sampling in February 2020						Sampling in September 2020					
	Innolab		Cooperl		Langage		Innolab		Cooperl		Langage	
	Tc (Threshold cycles)	Status	Tc (Threshold cycles)	Status	Tc (Threshold cycles)	Status	Tc (Threshold cycles)	Status	Tc (Threshold cycles)	Status	Tc (Threshold cycles)	Status
DBF-R1	No sampling						32,07	Positive	N/A	Negative	N/A	Negative
DBF-R2							32,40	Positive	N/A	Negative	N/A	Negative
DBF-R3							32,35	Positive	39,12	Positive	N/A	Negative
DAF-R1	33,11	Positive	N/A	Negative	N/A	Negative	32,50	Positive	N/A	Negative	N/A	Negative
DAF-R2	33,66	Positive	N/A	Negative	N/A	Negative	32,22	Positive	N/A	Negative	N/A	Negative
DAF-R3	33,56	Positive	N/A	Negative	N/A	Negative	32,64	Positive	N/A	Negative	N/A	Negative
Algae-R1	34,24	Positive	N/A	Negative	N/A	Negative	38,03	Positive	N/A	Negative	N/A	Negative
Algae-R2	34,27	Positive	N/A	Negative	N/A	Negative	37,96	Positive	N/A	Negative	N/A	Negative
Algae-R3	34,75	Positive	N/A	Negative	N/A	Negative	38,32	Positive	N/A	Negative	N/A	Negative

DBF = digestate before filtration; DAF = digestate after filtration; R1 = replicat 1

Table II: results for qPCR of Mycobacterium avium spp. paratuberculosis

that the results are very weakly positive. As expected, no bacteria was found by the numeration method, that means the bacteria detected by the PCR method were at a very low level and were not alive.

Samples sent in September 2020

All the results are negative for Langage and Cooperl (except a very weakly positive result for one sample (DBF-R3)). For Innolab, all the results (digestate before and after filtration and algae) were positive. However, the results show Tc values greater than 32, which means that the results are very weakly positive . As expected, no bacteria was found by the numeration method, that means the bacteria detected by the PCR method were at a very low level and were not alive.

Metagenomic analysis

In order to get a complete picture of the process (microbiological ecology from digestate before filtration until its consumption by microalgae) the samples (DBF, DAF, Algae) sent in September 2020 have been characterized with metagenomics approaches combining high throughput 16S sequencing and bioinformatics analysis.

Brief introduction to 16S metagenomics

The 16S ribosomal RNA is a highly conserved component of bacteria machinery and universal primers allow for the amplification of the DNA sequences containing both conserved and variables sequences (cf fig. 1). Primers designed in the conserved regions flanking V3 and V4 allow then for universal amplification of these V3 and V4 variables regions which are most commonly used for 16S metagenomics studies. The coupling of the 16S DNA amplification with the high throughput sequencing of the amplicons enable then the characterization of the bacterial population contained within a sample, whatever the bacterial species being cultivable or not, when comparing the sequences to 16S databases (Ribosomal Database Project, GreenGenes, SILVA, etc.). Similar sequences make clusters that are referred to as operational taxonomic units (OTU). OTU count and diversity gives a fair representation of the bacterial composition of the samples at either level of the bacterial classification (Domain- Phylum-Class-Order-Family-Genus-Species).

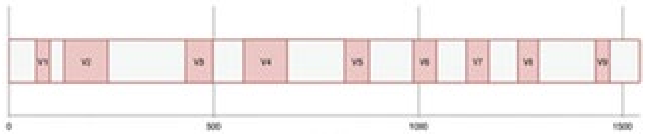

Fig.1 Representation of 1,5 Kb of 16S ribosomal. DNA Grey zone are conserved sequences , pink zone a variable sequences

Material and Methods

For all the samples, the DNA was extracted with the Dneasy power lyzer PowerSoil (Qiagen). DNA assay was performed with Qubit, a fluorometric based method.

The libraries were prepared according to the 16S metagenomic Sequencing library preparation for Illumina sequencing with the two following oligos:

16S Amplicon PCR Forward Primer = 5' CGTCGGCAGCGTCAGATGTGTATAAGAGACAGCCTACGGGNGGCWGCAG

16S Amplicon PCR Reverse Primer = 5'

GTCTCGTGGGCTCGGAGATGTGTATAAGAGACAGGACTACHVGGGTATCTAATCC

The library preparation were performed according to the instructions provided with the kit.

Sequencing was performed on a Miseq sequencing machine, set at 2x250 cycles, to generate a sequence (up to maximum of 500 nucleotides).

The 16S metagenome analysis were performed with the workflow FROGS , illustrated in Annexe III.

Results

DNA extraction:

16S metagenomics is based on the amplification of DNA extracted from samples to be analyzed. A summary of the mean result of DNA extraction is presented in Table III

The processes are different and cannot be compared between pilot sites, but two results deserve attention:

- The DAF from Innolab maintain a high level of extracted DNA compared to DBF which is not the case for the other two DAF (from Langage and Cooperl), which display a drastic reduction of the extracted DNA. Such result is in adequation with a bacteria filtered content of the DAF samples.

- The DAF-DNA extracted from Cooperl was not quantifiable (noted as not detected (nd)). Despite our attempts, no sequencing libraries could be produced from the DAF-Cooperl samples.

The two above observations are probably in relation to the filtration system used at the different sampling sites (300 KDa for Cooperl, 100 Kda for Langage and 10 microns for Innolab)

	Mean DNA concentration (ng/µl)		
	DBF	DAF	Algae
Innolab	34.2	22.6	1.4
LANGAGE (UK)	44.9	0.5	3.2
COOPERL (FR)	32.6	nd	116

Table III : Concentration of DNA from the different samples

16S Metagenomic Analysis

Raw sequences output varied according to samples from almost 59.000 for Innolab-DBF for the lowest, to more than 200.000 for Cooperl-Algae. These variations are inherent to the technique and particularly to the loading of the sequencing chip. The amount of data produced was, for some samples lower than expected, enough though have been obtained to go further in the analyses

Sample	Sequences output		
	Raw sequences	Quality Filtered	% kept
Innolab -DBF	155,170	129.748	83.6
INNOLAB -DAF	58.691	46.533	79.3
INNOLAB ALGAE	114.705	96.045	83.7
LANGAGE -DBF	90.101	66.194	74.3
LANGAGE -DAF	120.171	96.287	80.1
LANGAGE -ALGAE	89.037	67.759	76.3
COOPERL -DBF	90.101	70.974	78.8
COOPERL -ALGAE	212.597	174.060	81.8

Table IV: Output of sequences data

OTU clustering.

As very briefly described in the material and method, the operational taxonomic units are obtained by the clustering of the short 16S sequences (reads) obtained. Due to the high number of reads produced, clustering produce a high number of potential OTU. For ease of analysis, a cut-off is applied during the FROGS workflow to eliminate OTU which are very poorly represented, with less than 49 reads representing <0.0005% of read abundance.

After filtering, the mean OTU number dropped from 18621 to 323 kept for further analyses with variation from 135 OUT for Cooperl-Algae to 523 OUT for Innolab-DBF.

The number of OTU is reduced after filtration to 470 for Innolab-DAF but this number is increased from 249 to 371 for Langage DAF.

Rarefaction curves.

Metagenomic bacterial communities are, by definition, complex communities but their level of complexity can vary a lot. The question is then to characterize to which extent the data represents the diversity of our communities or only a fraction of this diversity.

This information can be inferred from the rarefaction curve which displays the relation between the increase in the number of OTU and the accumulation of data.

Sample	Sequences output		
	OTU Initial	with reads abundance <0.0005%	OTU kept
Innolab -DBF	22.603	22.080	523
INNOLAB -DAF	11.346	10.876	470
INNOLAB ALGAE	19.955	13.587	370
LANGAGE -DBF	12.617	12.368	249
LANGAGE -DAF	17.417	17.046	371
LANGAGE -ALGAE	18.372	18.196	176
COOPERL -DBF	12.760	12.466	294
COOPERL -ALGAE	33.898	33.763	135

Table V: Operational Taxonomic Units (OTU) identified and selected

When the identification of OTU is complete or almost complete, the increase of data in the system will lead to no more, or very little, increase in OTU number.

The rarefaction curves display the variation in OTU number observed between the samples. Despite significant differences in the size of the sequences produced for each sample, the completeness of the rarefaction curve seems roughly similar according to the general aspect of the curves, for most samples the last 10kb of data provide little additional OTU compared to the first 10 Kb (cf table VI). The rarefaction curves however do not reach a plateau meaning that additional data would marginally provide additional OTU, this is particularly true for the samples with 6 to 8 of additional OTU in the last 10 kb of the rarefaction curve. Most of the samples are close to saturation but the final number of OTU reached are differences between samples with a ratio of 2.8 (i.e 276/99) between the largest number and the lowest number of OTU identified (Innolab-Algae vs Cooperl Algae).

Phylogenetic affiliation of OTU

The species (eg: *Escherichia coli)* is the fundamental level of the classification of bacteria which is divided into 7 levels starting from Domain to Species (cf Annex 4).

At the phylum level, three phyla (Proteobacteria, Firmicutes and Bacteriodata) represent 88% of the OTU identified. These Phyla are present in most of the samples but their proportions vary greatly according to the investment sites and the step of the process considered (cf table VII a and b) with a significant reduction of the Firmicutes in all the Algae samples compared to the pre-filtered samples and correlatively an increase of the proteobacteria in the Algae sample. Furthermore,

Samples	OTU in first 10 Kb	OTU in Last 10 Kb	OTU
Innolab -DBF	219	2	260
INNOLAB -DAF	217	6	232
INNOLAB ALGAE	222	7	276
LANGAGE -DBF	101	2	137
LANGAGE -DAF	115	8	196
LANGAGE -ALGAE	101	7	145
COOPERL -DBF	125	6	157
COOPERL -ALGAE	52	3	99

Table VI: number of OTU (species level) identified in the first and last 10 kb of data and total number OTU identified for the different samples.

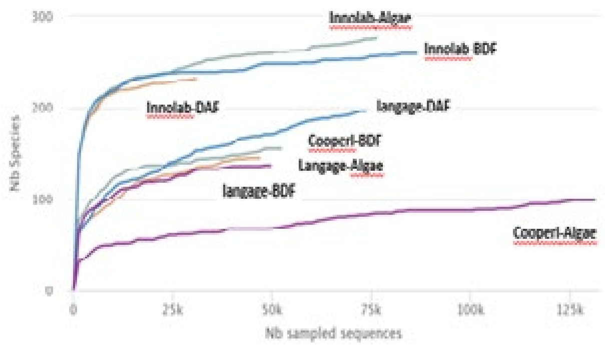

in Algae samples, some phyla poorly represented are retrograded below 1% of abundancy in the list and replaced by new phyla, that were extremely poorly represented in the pre or post filtered digestate (eg: Patescibacteria Actinobacteriota) or seemingly completely absent (eg: Planctomycocetota), suggesting an introduction during the process of algae production.

The Firmicutes dominate the pre-filtered samples (DBF) compared to the other phylum with level ranging from 46 to 77% of the OTU. This level drops from 77% to 42 % after Langage filtration and is compensated by a dramatic increase of Proteobacteria rising from 0.7 to 52% of OTU. No such dramatic variations were observed in Innolab filtered samples which display similar OTU composition for both DBF and DAF samples. The absence of library results for Cooperl DAF precludes any comparison.

The Algae samples are characterized by a large majority of OTU from the Proteobacteria phylum which outnumbered all the other phyla. A striking difference exists between the Cooperl Algae sample which still displays a large amount of OTU from the Firmicutes phylum whereas this phylum is barely present in Innolab and Langage algae samples. It can be speculated that heterotrophic culture conditions (for instance, organic carbon and low light) at Cooperl resulted in different microbiological ecology than in the autotrophic culture condition at Langage and Innolab (inorganic carbon and intense light)

Some phyla not identified in DBF or DAF popped up in the algae sample for Langage and Innolab but not for Cooperl.

Armatimonadetes is a terrabacteria phylum originally described solely on the basis of environmental 16S rRNA gene clone sequences. In 2011, a first bacterial strain belonging to the phylum was isolated from an aquatic plant in Japan.

Planctomycetota is a phylum of widely distributed bacteria, occurring in both aquatic and terrestrial habitats. Planctomycetes are members of the PVC superphylum, which encompass *Planctomycetes, Verrucomicrobiae, Chlamydiae, Lentisphaerae, Poribacteria*, OP3, WWE2. They play a considerable role in global carbon and nitrogen cycles, with many species of this phylum capable of anaerobic ammonium oxidation. Bacteria of this group are described in the microbiota of diverse environments and associated with macroalgae. The bacteria of the Planctomycetes group are considered relevant in different areas of research. Even though they have been described in association with the human oral and gut microbiota, some have also been presented as opportunistic pathogens too.

Patescibacteria is a new phylum very abundant in various environmental microbiota and frequently associated with surface waters but extremely poorly characterized and could constitute, itself a super phylum. This phylum has also been described in some animal microbiota studies.

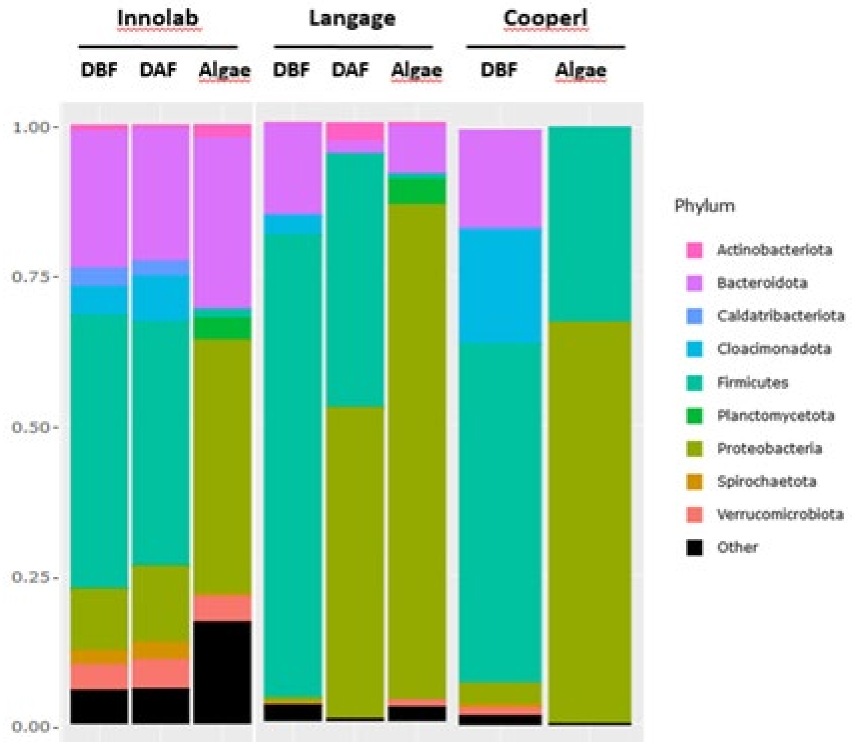

Fig 2: Representation of the OTU distribution at the Phylum level. For clarity only the nine most abundant phyla, containing 99% of the species, are represented.

	Phylogenic distribution at the Phylum level								
	Firm	Bact	Prot	Cloa	Verr	Cald	Spir	Syne	Ther
INNOLAB -DBF	46	23	10	4.7	4.0	3.0	2.4	1.6	1.5
INNOLAB DAF	41	22	12	7.0	5.0	3.0	2.5	1.6	1.6
LANGAGE -DBF	77	15	0.7	3.0	-	0.4	0.4	1.0	-
LANGAGE -DAF	42	2	52	-	-	-	-	-	-
COOPERL -DBF	56	16	3.5	18	1.2	0.5	-	0.8	-

Table VIIa

	Phylogenic distribution at the Phylum level								
	Firm	Bact	Prot	Plan	Verr	Arma	Myxo	Pate	Acti
Innolab -Algae	1.3	29	42	3.7	4.1	4.9	2.7	2.2	2.0
Langage -Algae	0.9	8.1	83	4.2	1.1	-	-	0.7	0.5
Cooperl -Algae	32	-	67	-	-	-	-	-	-

Table VIIb

Phylum acronym details: Firm : Firmicute **; Bact :** Bacteroides **; Prot :** Proteobacteria **; Cloa :** Cloacimonadota; **Verr:** Verrucomicrobiota; **Cald:** Caldatribacteriota; **Spir:** Spirochetota; Synergistota;**Ther:** Thermotogota; **Plan:** Planctomycetota **; Arma :** Armatimonadetes; **Myxo :** Myxococcota **;Pate :** Patescibacteria **; Acti :** Actinobacteriota

3 CONCLUSIONS

All the samples, irrespective of the matrix, the sampling period and the sampling site, were negative for *Campylobacter, Listeria monocytogenes, Yersinia enterocolitica* and *Salmonella*. These bacteria are considered as major zoonotic pathogens commonly detected in slurry from livestock.

When taking into account all the samples tested, regarding *Clostridium botulinum* and *Clostridiodes difficile*, there were more positive samples in September than in February. In February, no algae sample was positive for *Clostridoides difficile* while in September, regardless of the site, , at least one algae sample was positive at Langage and Innolab.

If we consider algae, which is the final step of the process that was analysed:

- Results for *C. botulinum* are either negative (Cooperl and Langage) or weakly positive (< 1.3 MPN/g for Innolab)

- Results for *C. difficile* are weakly positive for Langage and Innolab with a maximum of 1.6 MPN/g.

- Most of the *Mycobacterium paratuberculosis* results are negative for Langage and Cooperl, except for a very weakly positive result for one sample (DBF-R3). For Innolab, all the results (digestate before and after filtration and algae) were positive. However, the results show Tc values greater than 32, which means that the results are very weakly positive but the bacteria detected by PCR were not alive (the numeration by culture was negative).

Regarding *Clostridium botulinum* and *Clostridiodes difficile,* the detection rates in our study are rather lower than those found in other studies. For example, Le Marechal et al. (2019) analyzed manure and digestate from five farm biogas plants receiving animal manure in order to assess the occurrence and concentrations of *Clostridium botulinum* and *Clostridioides difficile.* In their study, the minimum and maximum detection rates in the digestates were 1.3 and 14.0 MPN/g for *C. botulinum* and 1.3 and 350 MPN/g for *C. difficile* and the rates were considered low according to the authors. Despite this, for these two spore-forming bacteria, a detection means that a risk to human health or animal health could not be ruled out, even at a low rate and in a context where they are not currently required by regulations in products used in animal feed.

The main findings of the NGS analysis were:

- At the phylum level, three phyla (Proteobacteria, Firmicutes and Bacteriodata) represent 88% of the OTU identified. These Phyla are present in most of the samples with a significant reduction of the Firmicutes in all the Algae samples compared to the pre-filtered samples and correlatively an increase of the proteobacteria in the algae sample. Furthermore, in algae samples some phyla poorly represented are retrograded below 1% of abundance in the list and replaced by new phyla, that was extremely poorly represented in the pre- or post-filtered digestates (eg: Patescibacteria or Actinobacteriota) or seemingly completely absent (ex: Planctomycocetota), suggesting an introduction during the process of algae production.

- Striking differences exist between the Cooperl algal sample which still has a large amount of OTUs from the phylum Firmicutes and the genus Bacillus. The 16S metagenomic analysis does not allow a precise identification of the bacteria present in this genus, (labeled as Bacillus cereus sensus lato) which includes pathogenic and non-pathogenic bacteria.

- The 16S metagenomicc analysis has revealed the presence of original microbiotes in the alguae samples. These new microbiotes display bacterial composition resulting most probably from the different inputs necessary for the algae production, i.e. the digestates but also from the algae inoculum and any additional components. These microbiotes would probably deserve a most extensive characterization.

- A formal assessment of the algae product's innocuity might be hampered by the fact that lot of bacteria identified in environmental samples are poorly known and barely cultivable for most of them

- Metagenomic evaluation of additionnal intrants, and not only digestates, might prove useful to work also on their sanitary quality and reduce the metagenome complexity and potential contaminations of the final product.

REFERENCES

Aghnatios, R., and Drancourt, M. (2015). Colonization of Hospital Water Networks by Gemmata massiliana, a New Planctomycetes Bacterium. Curr. Microbiol. 71, 317–320

Avery, L. M., Booth, P., Campbell, C., Tompkins, D., & Hough, R. L. (2012). Prevalence and survival of potential pathogens in source‐segregated green waste compost. Science of the Total Environment, 431, 128–138.

Avrain, L., Humbert, F., Sanders, P., Vernozy‐Rozand, C., & Kempf, I. (2004). Antimicrobial resistance in Campylobacter from pigs in French slaughterhouses. Revue De Médecine Vétérinaire, 155, 156–158.

Bagge E, Persson M, Johansson KE. (2010). Diversity of spore-forming bacteria in cattle manure, slaughterhouse waste and samples from biogas plants. Journal of Applied Microbiology, 109, 1549-65.

Böhnel, H. and Gessler, F. (2010) Botulinumtoxikosen – Infektionsrisiken für Mensch und Tier (unter besonderer Berücksichtigung von Schwein bzw. Rind). Nutztierpraxis aktuell 33, 14–18.

http://www.botulismus.org/index_htm_files/botulinumtoxikosen%2003032010.pdf (accessed 3 Mar 2015).

Boll C. Chronischer Botulismus. Tod aus der Biogasanlage. Wild und Hund 2011;10:14-9.

Boscher, E., Houard, E., & Denis, M. (2012). Prevalence and distribution of Listeria monocytogenes serotypes and pulsotypes in sows and fattening pigs in farrow‐to‐finish farms (France, 2008). Journal of Food Protection, 75, 889–895.

Cevallos‐Cevallos, J. M., Gu, G., Richardson, S. M., Hu, J., & van Bruggen, A. H. (2014). Survival of Salmonella enterica Typhimurium in water amended with manure. Journal of Food Protection, 77, 2035–2042.

Dharmasena, M., & Jiang, X. (2018). Isolation of toxigenic Clostridium difficile from animal manure and composts being used as biological soil amendments. Applied and environmental microbiology, 84(16), e00738-18.

Drancourt, M., Prebet, T., Aghnatios, R., Edouard, S., Cayrou, C., Henry, M., et al. (2014). Planctomycetes DNA in Febrile Aplastic Patients with Leukemia, Rash, Diarrhea, and Micronodular Pneumonia: FIG 1. J. Clin. Microbiol. 52, 3453–3455.

EFSA. (2016). EU summary report on zoonoses, zoonotic agents and food‐borne outbreaks 2015. EFSA Journal, 14, 1–231.

Erickson, M. C., Smith, C., Jiang, X., Flitcroft, I. D., & Doyle, M. P. (2014). Survival of Salmonella enterica and Listeria monocytogenes in manurebased compost mixtures at sublethal temperatures. Agriculture, Food and Analytical Bacteriology, 4, 224–238.

Escudié F., Auer L., Bernard M., Mariadassou M. , Cauquil L, Vidal K, Maman S., Hernandez-Raquet G. , Combes S., Pascal G. (2018) FROGS: Find, Rapidly, OTUs with Galaxy Solution. Bioinformatics, 34 , 1287–1294.

COMMISSION REGULATION (EU) No 142/2011 of 25 February 2011 implementing Regulation (EC) No 1069/2009 of the European Parliament and of the Council laying down health rules as regards animal by-products and derived products not intended for human consumption

Available on line:

https://eur- ex.europa.eu/LexUriServ/LexUriServ.do?uri=OJ:L:2011:054:0001:0254:EN:PDF

Fondrevez M., Labbé A., Houard E., Fravalo, P., Madec, F., Denis M. (2010) A simplified method for detecting pathogenic Yersinia enterocolitica in slaughtered pig tonsils. Journal of Microbiological Methods 83:244-249.

Froschle, B., Messelhausser, U., Holler, C., & Lebuhn, M. (2015). Fate of Clostridium botulinum and incidence of pathogenic clostridia in biogas processes. Journal of Applied Microbiology, 119, 936–947.

Gupta, R. S., Bhandari, V., and Naushad, H. S. (2012). Molecular Signatures for the PVC Clade (Planctomycetes, Verrucomicrobia, Chlamydiae and Lentisphaerae) of Bacteria Provide Insights into their Evolutionary Relationships. Front. Microbiol. 3, 327

Jäderlund, L., Sessitsch, A., & Arthurson, V. (2011). Persistence of two Campylobacter jejuni strains in soil and on spinach plants. Applied and Environmental Soil Science, 2011, 1–7.

Kempf, I., Kerouanton, A., Bougeard, S., Nagard, B., Rose, V., Mourand, G., Bengtsson, B. O. (2017). Campylobacter coli in organic and conventional pig production in France and Sweden: Prevalence and antimicrobial resistance. Frontiers in Microbiology, 8, 955.

Lebuhn, M., Effenberger, M., Garcés, G., Gronauer, A. and Wilderer, P.A. (2005) Hygienization by anaerobic digestion: comparison between evaluation by cultivation and quantitative real-time PCR. Water Sci Technol 52, 93–99.

Le Marechal C, Druilhe C, Reperant E, Boscher E, Rouxel S; Le Roux S; Poezevara T, Ziebal C, Houdayer C; Nagard B; Barbut F, Pourcher A-M, Denis M. (2019) Evaluation of the occurrence of sporulating and non-sporulating pathogenic bacteria in manure and in digestate of five agricultural biogas plants. MicrobiologyOpen.

Masse, D., Gilbert, Y., & Topp, E. (2011). Pathogen removal in farmscale psychrophilic anaerobic digesters processing swine manure. Bioresource Technology, 102, 641–646.

Milnes, A. S., Stewart, I., Clifton‐Hadley, F. A., Davies, R. H., Newell, D. G., Sayers, A. R., ... Paiba, G. A. (2008). Intestinal carriage of verocytotoxigenic Escherichia coli O157, Salmonella, thermophilic Campylobacter and Yersinia enterocolitica, in cattle, sheep and pigs at slaughter in Great Britain during 2003. Epidemiology and Infection, 136, 739–751.

Olsen, J. E. & Larsen, H. E. (1987). Bacterial decimation times in anaerobic digestions of animal slurries. BiologicalWastes 21, 153–168.

Patterson, S. K., Kim, H. B., Borewicz, K., & Isaacson, R. E. (2016). Towards an understanding of Salmonella enterica serovar Typhimurium persistence in swine. Animal Health Research Reviews, 17, 159–168.

Pinos, S., Pontarotti, P., Raoult, D., Baudoin, J. P., and Pagnier, I. (2016). Compartmentalization in PVC super-phylum: evolution and impact. Biol. Direct. 11, 38.

Tadesse, D. A., Bahnson, P. B., Funk, J. A., Thakur, S., Morrow, W. E., Wittum, T., ... Gebreyes, W. A. (2011). Prevalence and antimicrobial resistance profile of Campylobacter spp. isolated from conventional and antimicrobial‐free swine production systems from different U.S. regions. Foodborne Pathogens and Disease, 8, 367–374.

Hideyuki, T., Yasuhiro, T., Hiroaki, M., Mizuho, M., ; Xian-Ying, M., ; Satoshi, H., ; Kazuhiro, M., Yoichi, K. (2010). «Armatimonas rosea gen. nov., sp. nov., a Gram-negative, aerobic, chemoheterotrophic bacterium of a novel bacterial phylum, Armatimonadetes phyl. nov., formally called the candidate phylum OP10». International Journal of Systematic and Evolutionary Microbiology. 61 (6), 1442–7.

Thépault, A., Poezevara, T., Quesne, S., Rose, V., Chemaly, M., & Rivoal, K. (2018). Prevalence of thermophilic Campylobacter in cattle production at slaughterhouse level in France and link between C. jejuni bovine strains and campylobacteriosis. Frontiers in Microbiology, 9:471.

Whittington RJ, Marshall DJ, Nicholls PJ, Marsh IB, Reddacliff LA. (2004). Survival and dormancy of Mycobacterium avium subsp. paratuberculosis in the environment. Appl Environ Microbiol. ;70(5):2989-3004.

Whittington RJ, Marsh IB, Reddacliff LA. (2005).Survival of Mycobacterium avium subsp. paratuberculosis in dam water and sediment. Appl Environ Microbiol. ;71(9):5304-8.

APPENDICES

Appendix 1. Bacteriological analyses at Anses for the ALG-AD project Dec20

1) Detection and enumeration of Listeria monocytogenes:

For enumeration, 1 ml of a 10-fold dilution performed in half-strength Fraser broth (Biokar Diagnostics) is plated on Agar ListeriaOttavani and Agosti plates (ALOA) (BioMérieux, Craponne, France), as described in the NF EN ISO 7218 method (AFNOR, 2007a). Pre-enrichment in half-strength Fraser broth (Biokar Diagnostics) is undertaken in parallel at 30°C for 24 hr, followed by enrichment.

2) Detection and enumeration of thermotolerant Campylobacter:

For enumeration, 1 ml of a 10-fold dilution performed in Preston broth (Thermo Fisher Diagnostics SAS) is plated on a selective medium—CASA (BioMérieux, Craponne)—as described in ISO/TS 10272-2:2006 (ISO, 2006). Enrichment in Preston broth is undertaken in parallel, at 41.5°C in a microaerobic atmosphere (5% O2, 10% CO2, 85% N2) for 24 hr, followed by streaking on CASA. All of the plates are incubated at 41.5°C in a microaerobic atmosphere for 48 hr. The presence of typical colonies on the plates (small curved bacilli with spiralling "corkscrew" motility) is checked under a microscope.

3) Detection and enumeration of Salmonella:

Salmonella enumeration is performed using the most probable number (MPN) method as described in ISO/TS 6579-2:2012 (ISO, 2012). Enrichment in Peptone Water broth is undertaken in parallel at 37°C for 24 hr for detection using the NF U 47-100:2001 method (AFNOR, 2007b). All the enrichments (from detection and enumeration) are streaked on Rapid'Salmonella plates (BioRad Laboratories, Inc., Marnes-la-Coquette, France). The plates are incubated at 37°C for 24 hr. The presence of Salmonella is deduced from the following characteristics of the Rapid'Salmonella agar colonies, that is, fuchsia colonies.

4) Detection and enumeration of C. botulinum:

Due to the absence of selective media for the detection or enumeration of C. botulinum, a strategy different from the one used for other bacterial species is carried out for this pathogen by combining cultural and molecular methods. For detection of C. botulinum, regardless of the form (vegetative or spore cells), 25 g of each sample is 10-fold diluted in pre reduced trypticase peptone glucose yeast broth (TPGY) and homogenized using a Pulsifier (Microgen) for 15 s. The samples are then incubated at 37°C in an anaerobic chamber (A35; Don Whitley distributed by BioMérieux, Bruz, France) filled with anaerobic gas (10% H2, 10% CO2, 80% N2). After 24 hr of incubation, 1 ml is collected for DNA extraction. Enumeration is undertaken for positive samples using the MPN method. Twenty-five-gram frozen samples are 10-fold diluted in pre reduced TPGY, homogenized for 15 s using a Pulsifier (Microgen) and then 1:5 diluted in a serial dilution in 2 ml TPGY in triplicate using a 12-well microplate. The serial dilutions are incubated at 37°C in the anaerobic chamber for 24 hr. One ml of each well is then collected after 24 hr of incubation for DNA extraction. DNA extraction is performed using the NucleoSpin Soil DNA extraction kit (Macherey-Nagel, Hoerdt, France) according to the manufacturer's instructions. Detection of the encoding genes for botulinum neurotoxin (BoNT) types A, B, E, and F and a group III target is performed using real-time PCR with a Bio-Rad CFX96 thermal cycler using published primers and probes (Fach, Micheau, Mazuet, Perelle, & Popoff,

2009; Woudstra et al., 2015). Each PCR reaction includes a total volume of 25 µl, containing 5 µl of DNA template, 10 µl of Perfecta Tough mix (Quanta; VWR, Fontenay, France) and a final concentration of 600 nmol/L for primers and 400 nmol/L for probes. The thermal profile is as follows: 5 mins at 95°C, followed by 45 cycles of denaturation at 95°C for 15 s and an annealing extension at 55°C for 30 s. Each run will include positive and negative controls for each target as well as a commercial internal control (QuantiFast Pathogen + IC Kits; Qiagen, Courtaboeuf, France) used according to the manufacturer's instructions.

A sample is considered positive when a characteristic amplification is detected. For enumeration, the MPN/g value is estimated by an MPN calculator with a 95% confidence interval.

5) Detection and enumeration of Clostridium difficile:

For detection of C. difficile, regardless of the form (vegetative or spore cells), 1 g of each sample is 10-fold diluted in brain heart infusion (BHI; BioMérieux, Craponne) supplemented with 0.1% taurocholate (Sigma Aldrich, Lyon, France), cefoxitin (8 mg/L) and cycloserine (250 mg/L) (Oxoid). Tubes are incubated at 37°C in the anaerobic chamber. After 7 days of incubation, streaking from the enrichment is performed on ChromID C. difficile plates using a 10 µl loop. The plates are incubated for 48 hr at 37°C in the anaerobic chamber. Positive colonies are recognizable by their specific black color and/or form. For enumeration, 1 g of each sample is 10-fold diluted in BHI supplemented with 0.1% taurocholate, cefoxitin (8 mg/L), and cycloserine (250 mg/L). It is homogenized using a vortex and then 1:5 diluted in a serial dilution in 2 ml of BHI in triplicate using a 12-well microplate. After 7 days of incubation at 37°C in the anaerobic chamber, each well is streaked on a ChromID C. difficile plate. Positive colonies are recognizable by their specific black color and form. The MPN/g value is estimated by an MPN calculator with a 95% confidence interval.

6) Detection of Mycobacterium avium subsp. paratuberculosis

A) Detection of Mycobacterium avium subsp. paratuberculosis in methanisation matrix by PCR will be realized according to manufacturer's instructions.

Two steps are needed:

1) DNA extraction

2) PCR reaction

When a positive result is found by the PCR method, it is interesting to put the samples in culture (numeration method) to check whether the bacteria are alive or not.

B) Detection of Mycobacterium avium subsp. paratuberculosis in methanisation matrix by culture will be realized according to the method described in OIE Terrestrial Manual 2018 – Chapter 3.1.15. – Paratuberculosis (Johne's disease).

No chemical preservative is used. The faecal specimens can be frozen at –70°C. 1 g of faeces is transferred to a 50 ml tube containing 20 ml of sterile distilled water. The mixture is shaken for 30 minutes at room temperature. The larger particles are allowed to settle for 30 minutes. The uppermost 5ml of faeces suspension is transferred to a 50ml tube containing 20ml of 0.95% HPC. The tube is inverted several times to assure uniform distribution and allowed to stand undisturbed for 18 hours at room temperature. 0.1 ml of the undisturbed sediment is transferred to each of four slants of Herrold's medium, three with mycobactin and one without mycobactin. A smear may be made from the sediment and stained by the Ziehl-Neelsen method.

The slants are incubated for at least 4 months and observed weekly from the sixth week onwards." This method could be adapted after preliminary tests on methanisation matrix.

Annex 2.1: BD_result pathogenes Alg-AD_Anses July 2020

Annex 2.2 BD_result pathogenes Alg-AD_Anses Sept 2020
