# Supplementary material for: Scale-Up to Pilot of a Non-Axenic Culture of Thraustochytrids Using Digestate from Methanization as Nitrogen Source
Source: Mar Drugs. 2022 Aug 2;20(8):499. doi: 10.3390/md20080499 (PMC9410245; doi:10.3390/md20080499)
Supplement: Supplementary file 1 [file marinedrugs-20-00499-s001.zip › marinedrugs-1809880-reports/Interreg NWE ALG-AD project Seasonal variation of algal biomass cultivated using nutrient rich digestate.pdf]

Interreg
EUROPEAN UNION
North-West Europe
ALG-AD
European Regional Development Fund

THEMATIC PRIORITY

RESOURCE AND MATERIAL EFFICIENCY

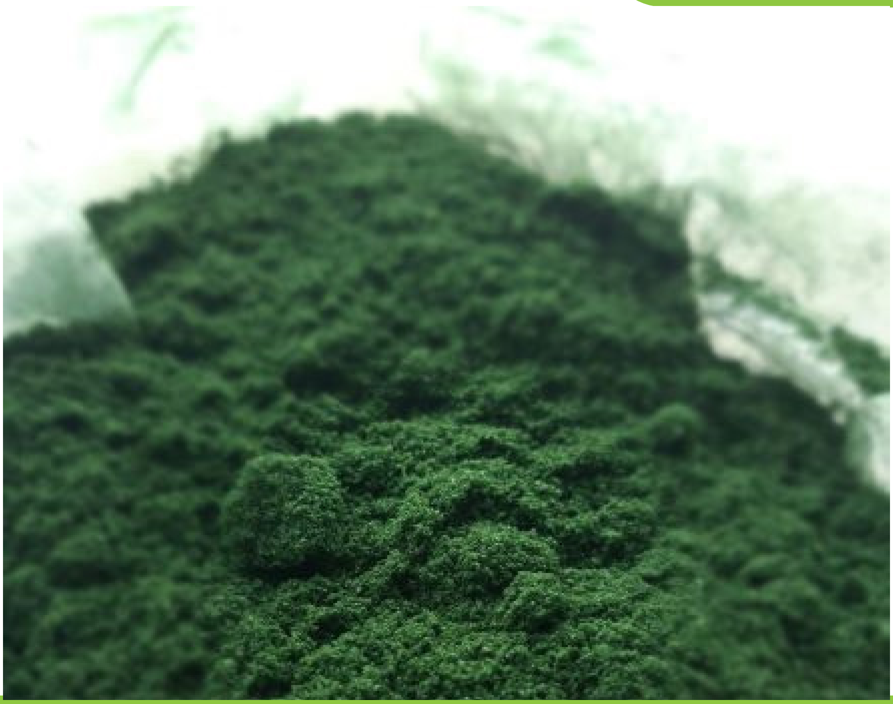

SEASONAL VARIATION OF ALGAL BIOMASS CULTIVATED USING NUTRIENT RICH DIGESTATE

EDITION : MAY 2022

Corresponding Authors

Rahul Vijay Kapoore (Lead author, r.kapoore@swansea.ac.uk), Swansea University, Singleton Park SA2 8PP, Swansea, Wales (UK)

Claudio Fuentes-Grünewald, Swansea University.

Philippe Soudant, CNRS, Technopôle Brest-Iroise, Rue Dumont d'Ur-ville, 29280 Plouzané (France)

Jai Sankar Seelam, Ghent University, Department of Green Chemistry and Technology, Campus Coupure, B6, Coupure Links 653, 9000 Ghent (Belgium)

Marcella Fernandes de Souza, Ghent University

Carole A. Llewellyn, Swansea University

Acknowledgements

The authors would like to thank Louise Hall for her excellent project management of the ALG-AD project

Please cite this document as follow:

Kapoore, R.V.*, Fuentes-Grünewald C., Soudant P., Seelam J.S., Souza M.F., Llewellyn C.A. 2021. Seasonal Variation variation of algal biomass cultivated using nutrient-rich digestate. Public Output report of the ALG-AD project, Swansea, June 2021.

Available online at www.nweurope.eu/projects/ALG-AD.

This document is an output from the ALG-AD project, which has received European Regional Development Funding through the INTERREG IVB NWE programme.

@ALG-AD project partnership, 2021, all rights reserved.

Executive summary

This document is a summary report on the seasonal variation in biochemical composition of algal biomass cultivated using nutrient-rich digestate (NRD). This document is part of the INTERREG North-West Europe funded ALG-AD project "Creating value from waste nutrients by integrating algal and anaerobic digestion technology".

In this report, information is presented regarding the characterisation of pre-treated digestate, as well as characterisation of the algal biomass produced over different seasons, specifically in winter 2019 and summer 2020. The document draws on analysis conducted on the pre-treated digestate and algal biomass cultivated using this digestate at three pilot sites in Devon (UK), Brittany (FR) and Flanders (BE). At Devon (UK), the pilot was located at the Langage dairy farm and algal biomass was cultivated using digestate provided by the farm's anaerobic digestion plant. At Brittany (FR), the pilot was located at Cooperl Arc Atlantique, and the digestate used resulted from the anaerobic digestion of pig manure. At Flanders (BE), the pilot was located at Innolab (Oostkamp) and used the digestate provided by the anaerobic digestion plant of AM-Power.

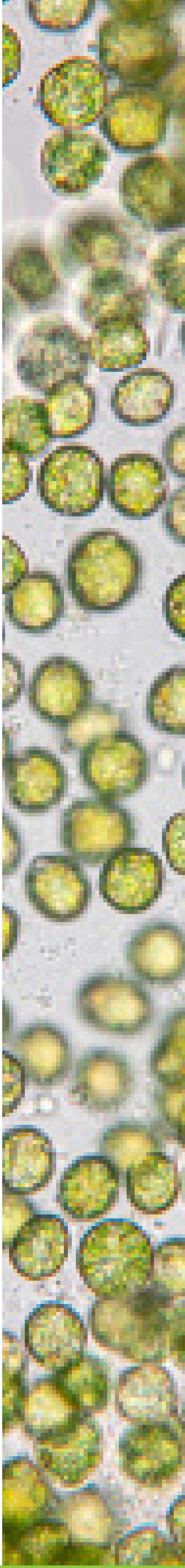

TABLE OF CONTENTS

INTRODUCTION 4
MATERIALS AND METHODS 5

- Biomass production 5
- Culture conditions 5
- Monitoring of enzymatic hydrolysis with the pH-Stat method 6
- Hydrolysis conditions 6
- Biochemical characterization of hydrolysates 7

RESULTS 9
ALCALASE-FREE TREATMENT 9

- Treatment of P20D biomass with Alcalase 2.4 L 10
- Treatment of P20F biomass 12
- Treatment of P20I biomass 13
- Fatty acid analysis 14

CONCLUSIONS 15
REFERENCES 17

INTRODUCTION

Anaerobic digestion of waste

Anaerobic digestion (AD) is the preferred solution to reduce the amount of animal and vegetable food waste produced across the EU. In the EU, around 88 million tonne of food waste is produced each year and this figure is predicted to increase to 120 million tonnes by 2022 (European Commission, 2021). Across North West Europe (NEW) there are currently around 2000 AD facilities, ranging in size from those processing 4,000 tons of waste each year up to 70,000 tons. They also produce a similar tonnage (volume) of nutrient rich digestate (NRD). While most NRD is returned to the land as a biofertiliser, there are areas within NWE which are subject to restrictions in the amount released in order to prevent run-off, eutrophication and pollution. The issue facing the region is what to do with the excess NRD. These restrictions are implemented through the European Nitrate Directive 91/676/EEC and the creation of Nitrate Vulnerable Zones (NVZs), many of which lie within the NWE area. NVZ legislation, however, is a barrier to the developing AD industry, and storage of NRD is becoming an acute issue with associated environmental and pollution risk and costs associated with disposal. As AD expands, therefore, there is an urgent need to find solutions to deal with excess production of NRD, and this challenge is most acute in the highly populated, intensively farmed NWE. EU environmental policy prioritises the transition to a 'circular economy' to boost competitiveness, growth and jobs through turning waste into resources, using materials in an efficient way and stimulating innovation in recycling.

The ALG-AD project (INTERREG-NWE funded - www.nweurope.eu/projects/ALG-AD) provides an eco-innovative solution combining algal technology with AD technology. Through the integration of the two technologies, the project seeks to overcome barriers to the development of AD practices and continued growth in the sector by helping to reduce nutrient pollution and at the same time create new sustainable products. ALG-AD project developed a sustainable circular economy platform to use of these waste nutrients to grow algal biomass which will in turn be used for the generation of protein, oil and peptides which can then be used in feed products. Further details on the ALG-AD project and the pre-processing of digestate for microalgal cultivation can be reviewed in the NRD pre-processing best practice guidelines (Fernandes et al., 2019).

Scope of the seasonal variation report

Microalgae present a significant potential for the remediation of liquid digestate (Chong et al., 2022; Fuentes-Grünewald et al., 2021; Seelam et al., 2022) due to their phosphorus and nitrogen high uptake rate during growth. Consequently, a nutrient rich digestate appears as an ideal and cost efficient substrate for the cultivation of microalgae. However, it is necessary to know the exact nutrient composition of digestate, which in too high amount can be toxic to the algae (Ayre et al., 2017; Stiles et al., 2018; Uggetti et al., 2014). In addition, it is also crucial to use an optimised digestate (N:P ratio in particular) at an adequate concentration to insure an efficient growth of algae in culture. The ALG-AD project focuses on optimising liquid digestate using a range of treatments prior its utilisation as a feedstock for microalgae cultivation. Further details on the digestate pre-processing step and characterisation can be reviewed in the NRD pre-processing best practice guidelines (Fernandes et al., 2019).

In order to be able to use the microalgae biomass grown on digestate for different bio-based applications such as feed for animals, key considerations are the reliability of the culture(s) and the quality and consistency of the biomass produced throughout the seasons. Specifically for the application as an animal feed, it is recommended to have a similar biochemical composition of the biomass throughout the year. This allows a consistent production of feed ingredients or pellets for animals such as pig, poultry or fish.

This document aims to describe the characteristics of the pre-treated nutrient-rich digestate used at the three pilot algal cultivation facilities in Devon (UK), Brittany (FR) and Flanders (BE), as well as the detailed biochemical analysis of biomass grown at different time points of the ALG-AD project. Recommendations are then presented, summarising the limitations and benefits of year-long cultivation, and potential and/or alternative bio-based applications on the basis of the seasonal variability of the biomass.

2. DIGESTATE CHARACTERISATION AND MICROALGAL CULTIVATION USING DIGESTATE

Langage AD, Devon (UK)

The digestate was sourced, pre-treated, characterised **(Table 1)** and stored under refrigerated conditions for use as required to cultivate the algal cultures. Further details of the digestate composition from Langage AD can be found in Fernandes et al. 2020 (Fernandes et al., 2020) (attached as annexe). The digestate was not characterised from a seasonal variation perspective, instead, the digestate from the Langage AD plant was sourced in large quantities at the beginning of the project, processed (filtered) and stored in a 4°C fridge. The digestate was then used as required for algal cultivation in photobioreactors (PBR's). The algal biomass was cultured and harvested in two different seasons; the biochemical composition of the algal biomass is discussed below from a seasonal variation perspective. Several suggestions regarding the digestate pre-treatment, transportation, storage and use of digestate for microalgal cultivation can be found in the best practice documents attached as annexes and in Fernandes et al. 2020 and Fuentes-Grünewald et al 2021 (Fernandes et al., 2020; Fuentes-Grünewald et al., 2021).

Table 1: Characterisation of digestate (sampled on Dec 2020) from Langage AD.

Compund (Unit)	Langage AD Data
Digestate Origin	Food Waste and Dairy factory waste
Dry Matter (%)	0.97
DRY ORGANIC MATTER (%)	15.75
ACETIC ACID (MG/KG)	< 34
PROPIONIC ACID (MG/KG)	< 16
ISOBUTYRIC ACID (MG/KG)	< 9
BUTYRIC ACID (MG/KG)	< 7
ISOVALERIC ACID (MG/KG)	< 11
VALERIC ACID (MG/KG)	< 12
CAPROIC ACID (MG/KG)	< 24
PH	9.12
CONDUCTIVITY (MS/CM)	28.89
ELEMENTAL ANALYSIS (MG/L)	B 0.800; As 0.059; Ca 7.75; Cd < 0.005; Co 0.036; Cr < 0.025; Cu < 0.050; Fe 6.80; Hg < 0.005; K 2090; Mg 1.60; Mn < 0.050; Mo 0.043; Na 1960; Ni 0.140; P 33.7; Pb < 0.050; S 26.6; Se < 0.005; Si 7.60; Sn < 0.050; Zn 0.136 and Cl 2980
AMMONIACAL NITROGEN (G N/KG)	3.36
TOTAL NITROGEN (KG/1000KG FM)	3.40
PHOSPHATE (MG/L)	95
SALINITY (G/KG)	7.25

UGhent-Innolab outsourced AD digestate analysis, Flanders (BE)

The liquid fraction of digestate (after on-site processing) was provided for cultivation at different time points, July 2018, December 2019 and February 2020. These liquid samples were paper-filtered, analysed **(Table 2)** and stored at room temperature conditions for use as required to cultivate the algal cultures. The digestate composition was found to be having a seasonal effect, however, the fluctuations within macro-nutrient characteristics were less pronounced. Thus, this aspect is not reported from a seasonal variation perspective. The paper filtered liquid fraction of digestate was then used for algal cultivation in PBR's both at lab-scale and pilot scale. The algal biomass was cultured and harvested at different seasons; the biochemical composition results of the algal biomass are discussed from a seasonal variation perspective. For targeted compounds such as lipids and DHA, biochemical analyses were performed for most of the produced cultures and different sampling during the culture.

Table 2 Characterisation of digestate (sampled on Dec 2019) from UGhent-Innolab, Flanders (BE)

Compund (Unit)	UGent-Innolab outsourced AD DATA
Digestate Origin	Organic biological waste (industrial food waste and source segregated food waste)
DRY MATTER (%)	1.64
DRY ORGANIC MATTER (% DM)	19.84
ACETIC ACID (MG/KG)	< 34
PROPIONIC ACID (MG/KG)	< 16
ISOBUTYRIC ACID (MG/KG)	< 9
BUTYRIC ACID (MG/KG)	< 7
ISOVALERIC ACID (MG/KG)	< 11
VALERIC ACID (MG/KG)	< 12
CAPROIC ACID (MG/KG)	< 24
PH	8.51
CONDUCTIVITY (MS/CM)	36.92
ELEMENTAL ANALYSIS (MG/L)	B 2.52; As <0.05; Ca 52.5; Cd < 0.005; Co 0.132; Cr < 0.025; Cu < 0.050; Fe 3.59; Hg < 0.005; K 3330; Mg 2.68; Mn 0.036; Mo 0.060; Na 3640; Ni 0.272; P 165; Pb < 0.050; S 36.2; Se < 0.005; Si 19.0; Sn < 0.050; Zn 0.545 and Cl 3380
AMMONIACAL NITROGEN (G N/KG)	3.06
TOTAL NITROGEN (KG/1000KG FM)	3.15

COOPERL sourced digestate analysis, Brittany (FR) (sampled on Dec 2020)

The digestate used at the pilot site was provided by Cooperl Arc Atlantique (France) and was the result of the anaerobic digestion of pig manure. For microalgal cultivation, the digestate was treated using ultra-filtration at a pore size of 300 kDa. The composition of the raw digestate can be found in **Table 3.** The filtered digestate was stored in a 4°C fridge. We used two batches of digestate; one for the cultures from July 2020 till September 2020 and another one from September 2020 till January 2021. Nevertheless, we considered these 2 batches were insufficient to address the question of digestate seasonal influence on cultures. Indeed, no obvious difference could be observed between cultures produced with these 2 batches (see results below). The algal biomass was cultured and harvested from July 2020 till January 2021 and analysed for protein, lipid and fatty acid contents allowing a discussion on biomass seasonal variations. We assumed that culture variability batch to batch likely prevail over digestate composition variability.

Table 3 Characterisation of digestate from COOPERL, Brittany (FR)

Compund (Unit)	UGent-Innolab outsourced AD DATA
Digestate Origin	Organic biological waste (industrial food waste and source segregated food waste)
DRY MATTER (%)	0.56
DRY ORGANIC MATTER (% DM)	28.36
ACETIC ACID (MG/KG)	< 35
PROPIONIC ACID (MG/KG)	< 16
ISOBUTYRIC ACID (MG/KG)	< 9
BUTYRIC ACID (MG/KG)	< 7
ISOVALERIC ACID (MG/KG)	< 11
VALERIC ACID (MG/KG)	< 12
CAPROIC ACID (MG/KG)	< 24
PH	8.21
CONDUCTIVITY (MS/CM)	17.92
ELEMENTAL ANALYSIS (MG/L)	B 0.51; As <0.05; Ca 47.5; Cd < 0.005; Co 0.018; Cr < 0.025; Cu < 0.050; Fe 2.16; Hg < 0.005; K 1600; Mg 8.7; Mn 0.050; Mo 0.012; Na 690; Ni 0.072; P 34.4; Pb < 0.050; S 22.8; Se < 0.005; Si 27.4; Sn < 0.050; Zn 0.250 and Cl 875
AMMONIACAL NITROGEN (G N/KG)	1.93
TOTAL NITROGEN (KG/1000KG FM)	1.98
PHOSPHATE (MG/L)	103
SALINITY (G/KG)	2.96

2 CHARACTERISATION OF ALGAL BIOMASS

Langage AD, Devon (UK) - Algal biomass analysis

Biomass was grown, harvested, and concentrated at Langage AD, according to the procedures outlined in the algal cultivation and downstream processing best practice guidelines, available on the ALG-AD website. The biomass cultivated at Langage was characterised at two specific time points to assess for seasonal variation – during the winter (January 2020) and summer (June 2020).

Proteins and carbohydrates

The Scenedesmus sp. biomass grown on digestate (~2.5%) at different seasons (winter (Jan_20) and summer (Jul_20) have a similar biochemical composition in terms of proteins and carbohydrates content (**Figure 1**). A key finding is that the protein content in biomass grown on digestate at different seasons has an average of 20% higher content than the Scenedesmus biomass grown on standard commercial media (e.g. Cell-hi F2P™, Varicon Aqua). Similar results were observed for the green microalgae Chlorella vulgaris grown under the same treatment (digestate) and control conditions (Cell-hi F2P™), these results were recently published and can be found in Fuentes-Grünewald et al., 2021. 2021 (Fuentes-Grünewald et al., 2021). It can be argued that this increment in protein content could be due to the easy assimilation of nitrogen in the form of ammonium (found in digestate) rather than nitrate (found in commercial media) by photosynthetic organisms (Fuentes-Grünewald et al., 2021). This easy assimilation means less energy spent during the proteins construction, consequently a higher content can be expected if a simple nitrogen source such as ammonium is used. The carbohydrate content was found to be similar between treatments (digestate) and control (Cell-hi F2P™) conditions. These promising results allow us to predict a stable, reliable and quality biomass produced throughout the year when digestate is used for autotrophic cultivation.

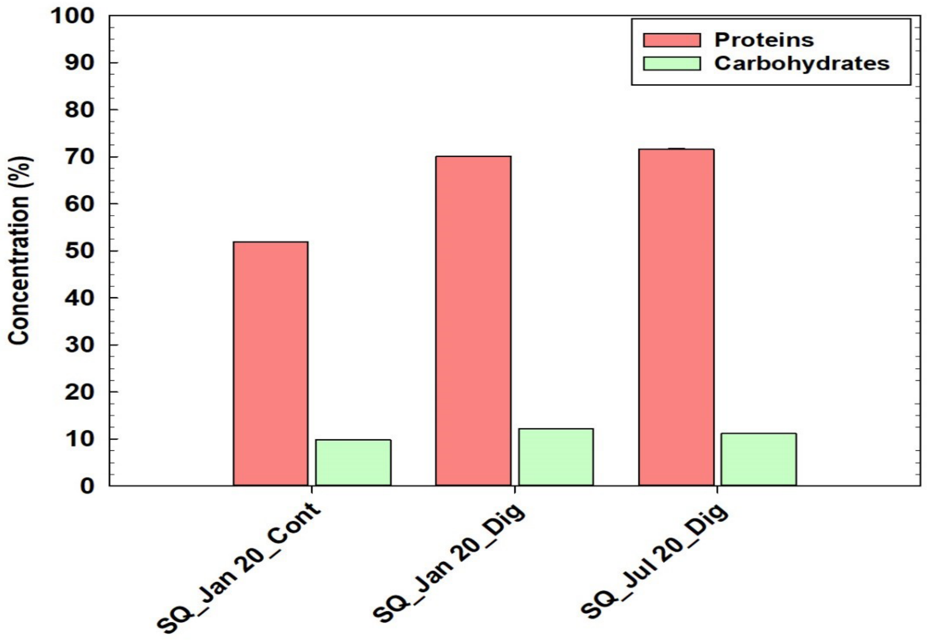

Figure 1 Influence of seasonal variations on the recovery of total proteins and total carbohydrates from Scenedesmus sp. biomass grown on digestate (~2.5%) at different seasons (winter (Jan_20) and summer (Jul_20). The X-axis represents Scenedesmus sp. biomass and time series (in months), where SQ_Jan 20_Cont = Scenedesmus sp. biomass grown in January 2020 using commercial medium (Cell-hi F2P™, Varicon Aqua) referred here as control; SQ_Jan 20_Dig = Scenedesmus sp. biomass grown in January 2020 on digestate (~2.5%) and SQ_Jul 20_Dig = Scenedesmus sp. biomass grown in July 2020 on digestate (~2.5%). The Y-axis represents concentration of total proteins and carbohydrates in % dry weight

Pigments

In the case of pigment composition of the Scenedesmus biomass grown on digestate and in commercial media, results showed a similar trend as the protein content, having a higher and similar pigment concentration (chlorophylls and carotenoids) among the seasons for the biomass grown on digestate compared to the biomass grown on commercial media (**Figure 2**).

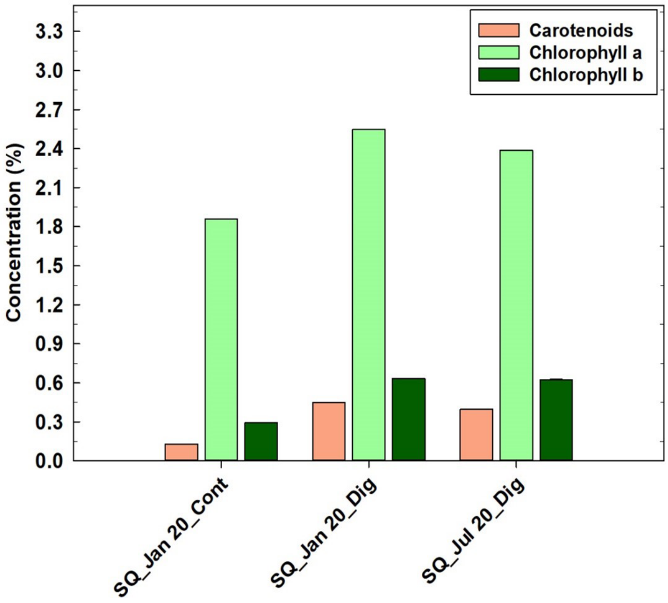

Figure 2: Influence of seasonal variations on the recovery of total carotenoids, chlorophyll a and chlorophyll b from Scenedesmus sp. biomass grown on digestate (~2.5%) at different seasons (winter (Jan_20) and summer (Jul_20). The X-axis represents Scenedesmus sp. biomass and time series (in months), where SQ_Jan 20_Cont = Scenedesmus sp. biomass grown in January 2020 using commercial medium (Cell-hi F2P™, Varicon Aqua) referred here as control; SQ_Jan 20_Dig = Scenedesmus sp. biomass grown in January 2020 on digestate (~2.5%) and SQ_Jul 20_Dig = Scenedesmus sp. biomass grown in July 2020 on digestate (~2.5%). The Y-axis represents concentration of total carotenoids, chlorophyll a and chlorophyll b in % dry weight.

Fatty acids

The total fatty acids content showed a similar trend to the rest of macromolecules (proteins and carbohydrates mainly). Briefly, higher concentration was achieved in biomass grown on digestate during the winter season (SQ_Jan_20_Dig) compared to the control (SQ_Jan_20_Cont). In terms of seasonality, there is a notable difference with a higher content of fatty acids in winter time compared to summer (**Figure 3**). This could be explained by the climatic conditions found in summer, with higher solar radiation and pleasant temperatures in Southern England (Devon), the cells need less energy reserve, using mainly carbohydrates energy for the different metabolic requirements.

Regarding individual fatty acid profiles, as it can be seen in **Figure 4**, a relatively high concentration of Linolenic acid (C18:3n6) was found in Scenedesmus biomass. Similar to the trend found in most of the macromolecules content in this biomass, Linolenic acid content was higher in the biomass grown on digestate than the biomass grown on commercial media, and also a quite similar content was found between the seasons.

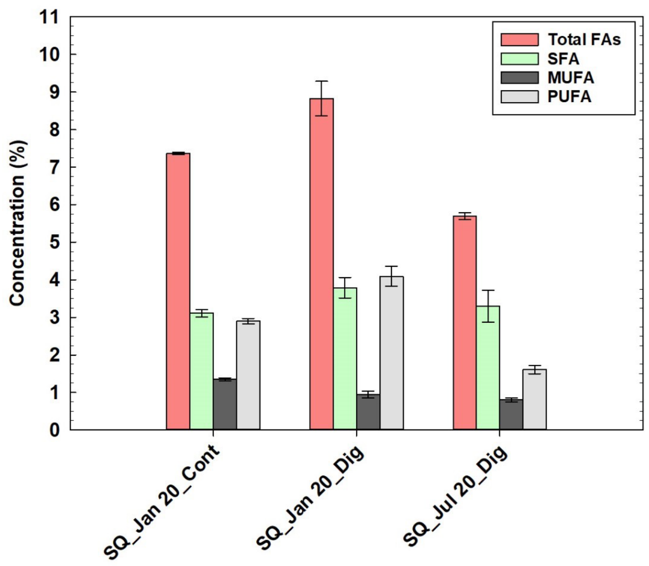

Figure 3 Influence of seasonal variations on the recovery of total (FAs), saturated (SFA), monounsaturated (MUFA) and polyunsaturated fatty acids (PUFA) from Scenedesmus sp. biomass grown on digestate (~2.5%) at different seasons (winter (Jan_20) and summer (Jul_20). The X-axis represents Scenedesmus sp. biomass and time series (in months), where SQ_Jan 20_Cont = Scenedesmus sp. biomass grown in January 2020 using commercial medium (Cell-hi F2P™, Varicon Aqua) referred here as control; SQ_Jan 20_Dig = Scenedesmus sp. biomass grown in January 2020 on digestate (~2.5%) and SQ_Jul 20_Dig = Scenedesmus sp. biomass grown in July 2020 on digestate (~2.5%). The Y-axis represents concentration of total (FAs), saturated (SFA), monounsaturated (MUFA) and polyunsaturated fatty acids (PUFA) in % dry weight.

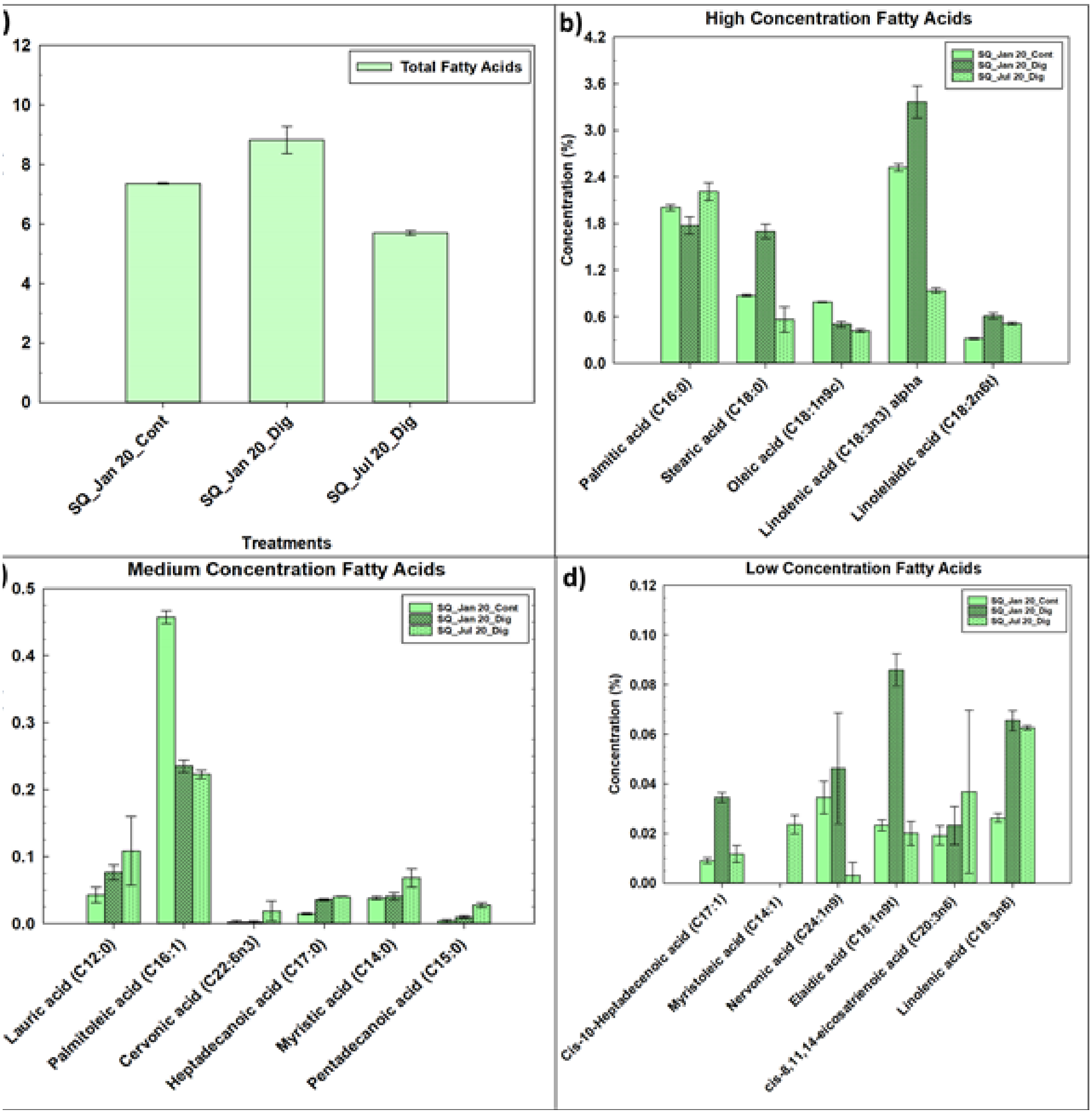

Figure 4 Influence of seasonal variations on the recovery of total (a), high (b), medium (c) and low concentration (d) fatty acids from Scenedesmus sp. biomass grown on digestate (~2.5%) at different seasons (winter (Jan_20) and summer (Jul_20). For figure 4a, the X-axis represents Scenedesmus sp. biomass and time series (in months), where SQ_Jan 20_Cont = Scenedesmus sp. biomass grown in January 2020 using commercial medium (Cell-hi F2P™, Varicon Aqua) referred here as control; SQ_Jan 20_Dig = Scenedesmus sp. biomass grown in January 2020 on digestate (~2.5%) and SQ_Jul 20_Dig = Scenedesmus sp. biomass grown in July 2020 on digestate (~2.5%). For figure 4b, c and d, the X-axis represents fatty acids recovered from Scenedesmus sp. biomass grown on control and digestate medium at different seasons (winter (Jan_20) and summer (Jul_20). The Y-axis represents concentration of total (a), high (b), medium (c) and low concentration (d) fatty acids in % dry weight.

Elemental composition

In the elemental composition of Scenedesmus biomass grown on digestate, again similar results were found in summer and winter. In this case, the biomass grown on the commercial medium shows a relatively high carbon content compared to biomass grown on digestate.

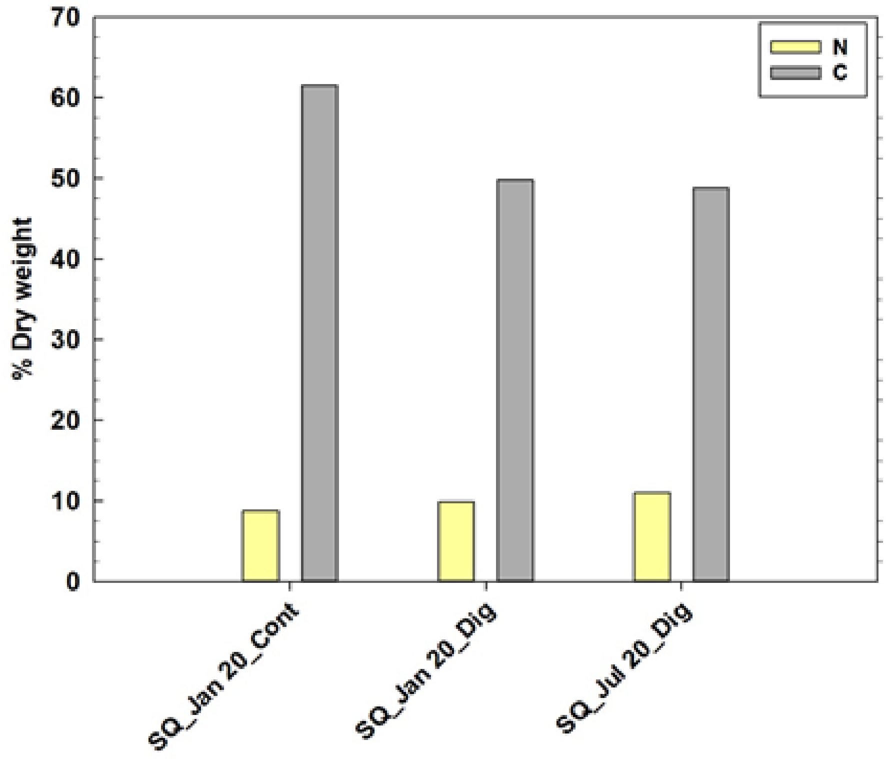

Figure 5 Influence of seasonal variations on the recovery of total C and total N contents from Scenedesmus sp. biomass grown on digestate (~2.5%) at different seasons (winter (Jan_20) and summer (Jul_20). The X-axis represents Scenedesmus sp. biomass and time series (in months), where SQ_Jan 20_Cont = Scenedesmus sp. biomass grown in January 2020 using commercial medium (Cell-hi F2P™, Varicon Aqua) referred here as control; SQ_Jan 20_Dig = Scenedesmus sp. biomass grown in January 2020 on digestate (~2.5%) and SQ_Jul 20_Dig = Scenedesmus sp. biomass grown in July 2020 on digestate (~2.5%). The Y-axis represents concentration of total C and total N contents in % dry weight.

UGhent-Innolab, Flanders (BE) - Algal biomass analysis

Two different reactor configurations were used within UGent-Innolab site to compare lab-scale and pilot scale performances of microalgae growth. The December (winter) studies were performed in a lab-scale PBR (Labfors 5 Lux LED stirred tank reactor, Infors AG, Bottmingen, Switzerland) with working volume of 3 L under controlled lighting (sinusoidal light with a maximum intensity of 100 μmol/m2.s and light/dark photoperiod of 16:8 h) and temperature conditions (18 – 23 °C) with paper-filtered liquid fraction of digestate (PFD) as a nutrient source. A mixed consortium of digestate-acclimatized Chlorella vulgaris and Desmodesmus sp. was cultivated in diluted PFD under non-axenic conditions. The volumetric loading of digestate was 10% v/v PFD (N: 230 mg/L) and additional phosphorus (K2HPO4 solution) was added to balance the N/P ratio and to maintain exponential growth. mum and maximum of 19 °C and 35 °C. A control experiment was also carried out with modified-WC medium (Guillard & Lorenzen, 1972) composed of (mg/L) 36.76 CaCl2, 8.71 K2HPO4, 36.97 MgSO4·7H2O, 12.60 NaHCO3, 85.01 NaNO3, 4.36 Na2·EDTA, 3.15 FeCl3·6H2O, 0.010 CuSO4·5H2O, 0.18 MnCl2·4H2O, 0.022 ZnSO4·7H2O, 0.010 CoCl2·6H2O, 0.006 Na2MoO4·2H2O and 1.00 H3BO3 without additional vitamins. This modified-WC medium was added in intermittent doses to maintain exponential growth, reaching a total nitrogen concentration of 60 mg/L. The harvest of these lab-scale trials was done during December 2019 with Desmodesmus sp. being the dominant culture (approximately 90%) in digestate experiments and Chlorella vulgaris (approximately 70%) in the control experiments.

The August 2020 cultivation was performed in a pilot-scale PBR (LGem horizontal reactor of 550 L). The nitrogen loading was adjusted to 2.5% v/v (N: 80 mg/L) coming solely from digestate. The average temperature in the greenhouse during cultivation was 26 °C, with minimum and maximum of 19 °C and 35 °C.

Proteins and carbohydrates

The August 2020 batch had a lower protein and higher carbohydrate content than the December 2019 batch, even though both were grown on digestate. It is very likely that the duration of the batch, rather than the season, was responsible for the difference in composition observed, as the August batch was run for over a week on low N concentrations, a condition that likely triggered carbohydrate accumulation, while the December batch was harvested in the early stages of stationary phase. Interestingly, when comparing both the December batches, the control run had a lower protein content than the digestate one, which could be attributed to the easier uptake of ammonium compared to nitrate, as already discussed on page 8.

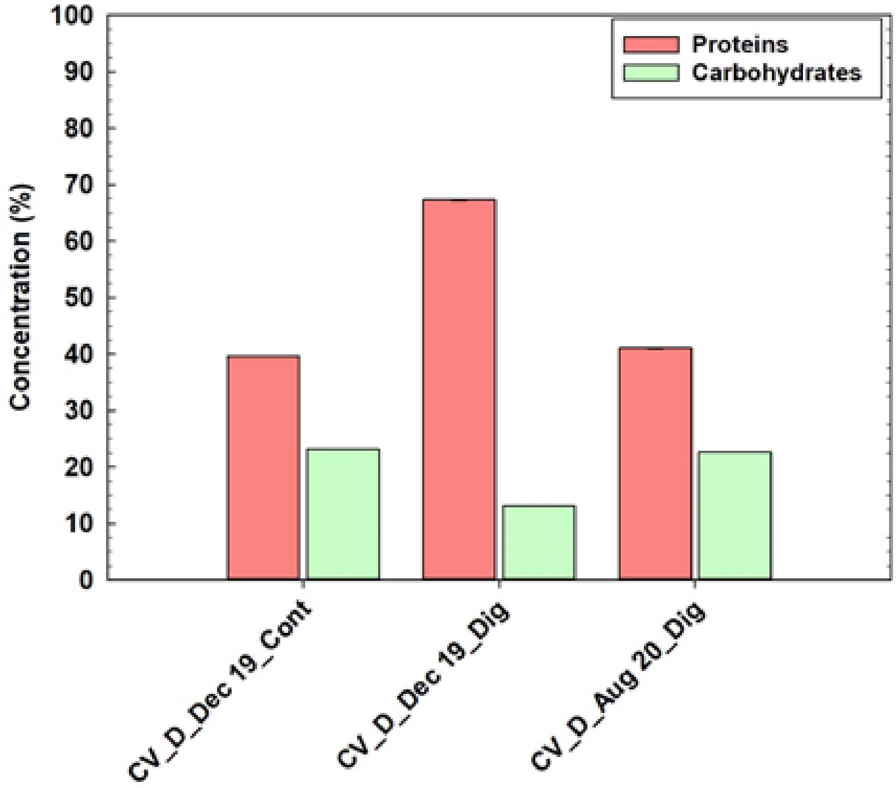

Figure 6 Protein and carbohydrates content of the microalgae biomass produced from different batches: (i) Dec 19_Cont was grown in a lab-scale continuously stirred PBR of 3 L under sinusoidal light with a maximum intensity of 100 µmol/m2.s and light/dark photoperiod of 16:8 h and controlled temperature conditions (18 – 23°C) in modified-WC medium; (ii) Dec 19_Dig was grown at the same conditions as (i) but with 10% (v/v) paper-filtered liquid fraction of digestate as a nutrient source; (iii) Aug 20_Dig was grown in a pilot-scale tubular PBR of 500 L with 2.5% (v/v) paper-filtered liquid fraction of digestate as a nutrient source and an average temperature in the greenhouse during cultivation of 26°C.

Pigments

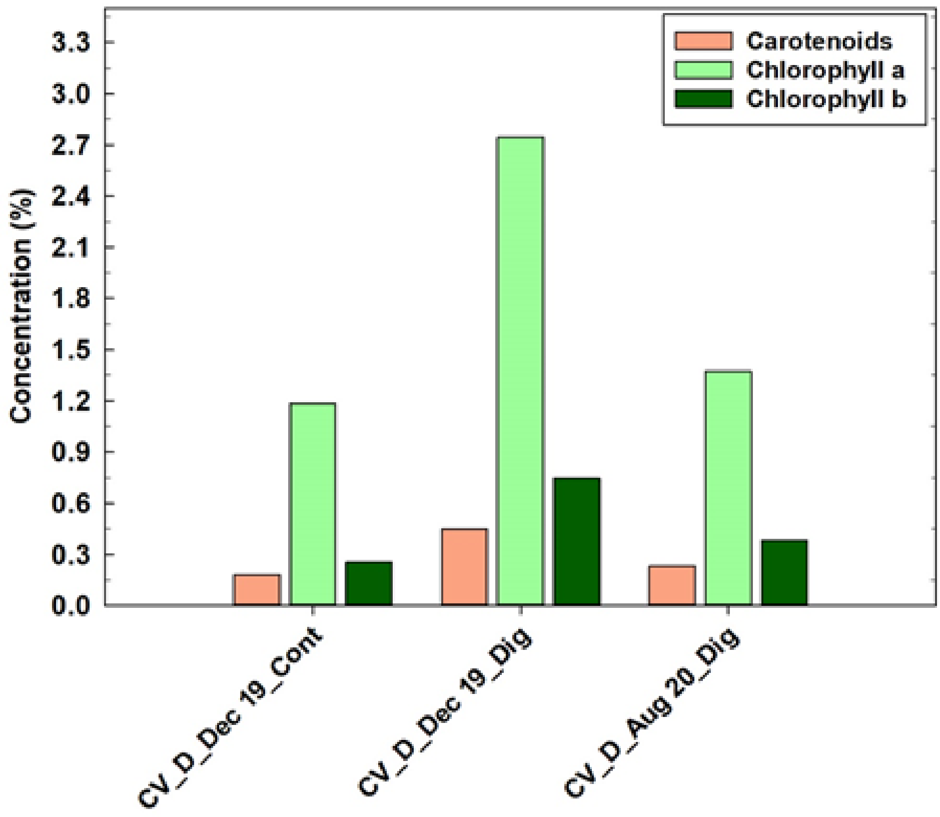

Figure 7 Pigment content of the microalgae biomass produced from different batches: (i) Dec 19_Cont was grown in a lab-scale continuously stirred PBR of 3 L under sinusoidal light with a maximum intensity of 100 µmol/m2.s and light/dark photoperiod of 16:8 h and controlled temperature conditions (18 – 23°C) in modified-WC medium; (ii) Dec 19_Dig was grown at the same conditions as (i) but with 10% (v/v) paper-filtered liquid fraction of digestate as a nutrient source; (iii) Aug 20_Dig was grown in a pilot-scale tubular PBR of 550 L with 2.5% (v/v) paper-filtered liquid fraction of digestate as a nutrient source and an average temperature in the greenhouse during cultivation of 26°C.

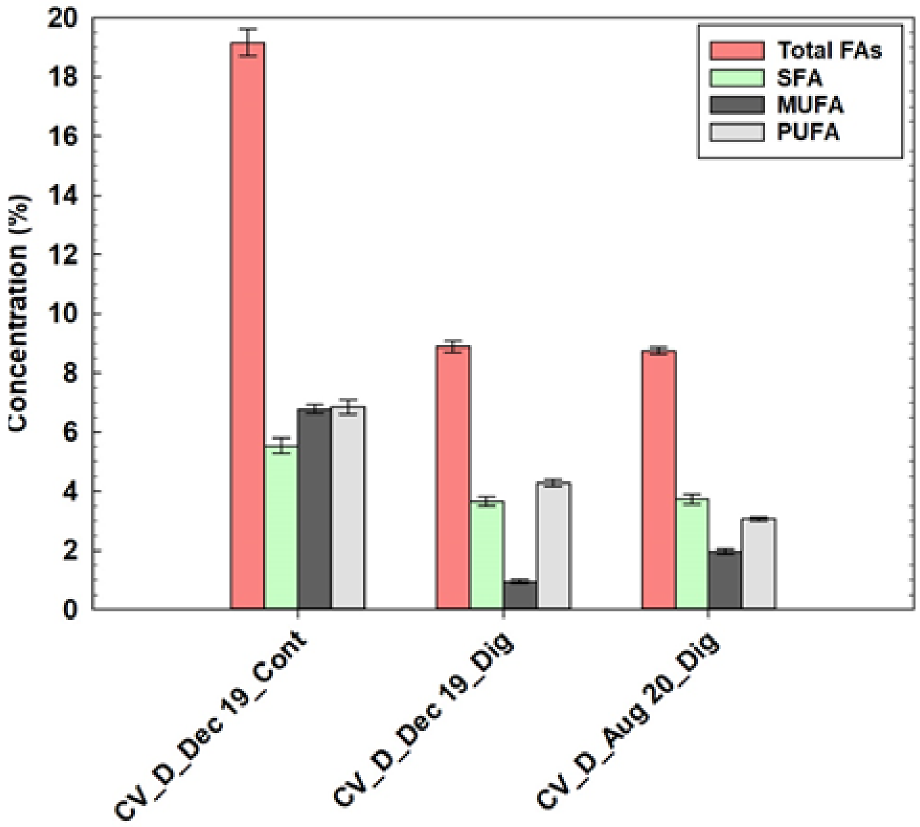

Figure 8 Fatty acids content of the microalgae biomass produced from different batches: (i) Dec 19_Cont was grown in a lab-scale continuously stirred PBR of 3 L under sinusoidal light with a maximum intensity of 100 μmol/m2.s and light/ dark photoperiod of 16:8 h and controlled temperature conditions (18 – 23°C) in modified-WC medium; (ii) Dec 19_Dig was grown at the same conditions as (i) but with 10% (v/v) paper-filtered liquid fraction of digestate as a nutrient source; (iii) Aug 20_ Dig was grown in a pilot-scale PBR of 550 L with 2.5% (v/v) paper-filtered liquid fraction of digestate as a nutrient source and an average temperature in the greenhouse during cultivation of 26°C

The fatty acids composed of SFA, PUFA and MUFAs presented a slightly different trend in the performed lab- and pilot-scale experiments. This can be possibly explained by the different composition of the harvested biomass, as both the batches cultivated on digestate had Desmodesmus sp. as the dominant culture (approximately 90%) while Chlorella vulgaris was dominant (approximately 70%) in the control experiment. Different species may have different accumulation patterns for storage molecules, which can be the reason behind the higher lipid content in the Dec 19_Cont sample

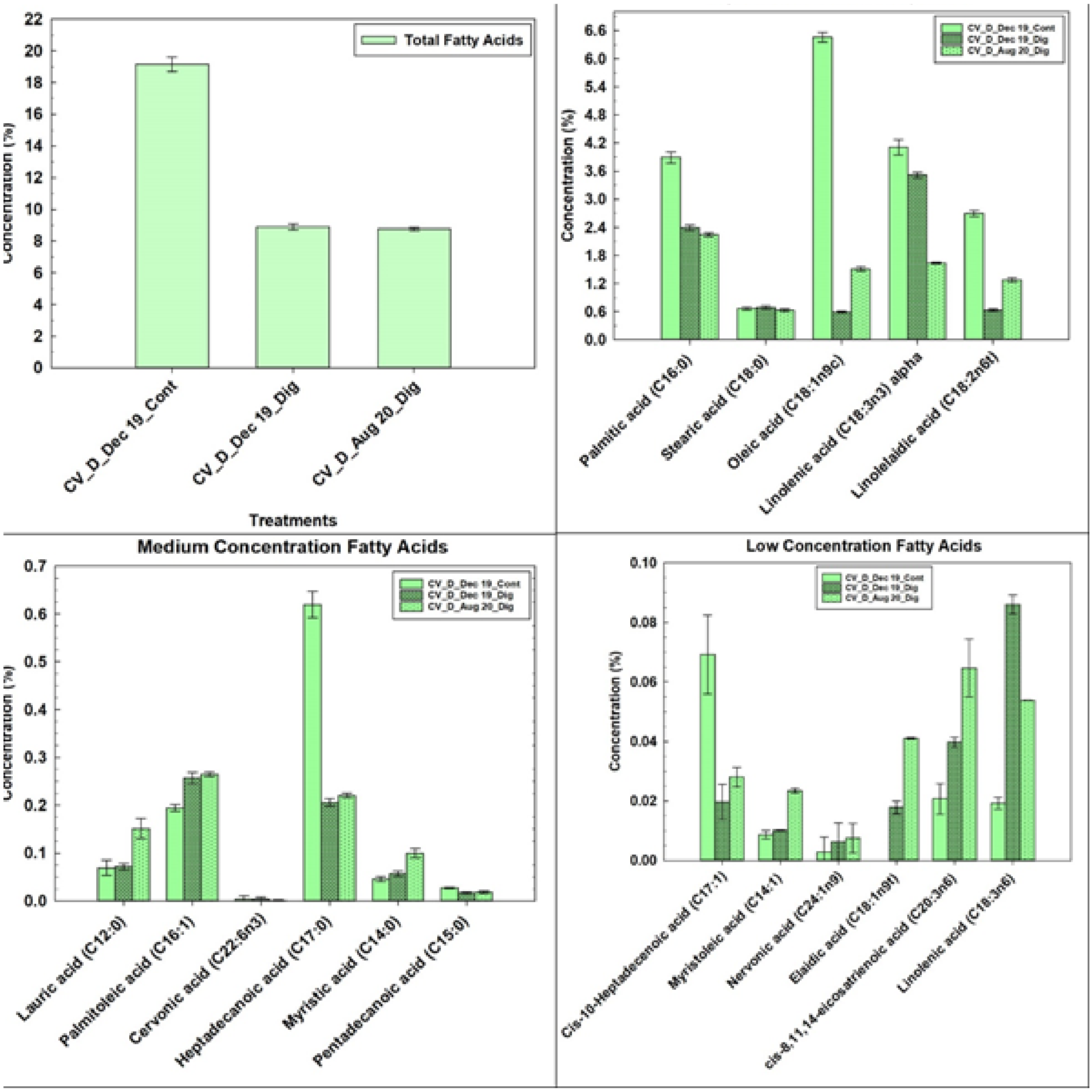

Figure 9 Fatty acids profile of the microalgae biomass produced from different batches: (i) Dec 19_Cont was grown in a lab-scale continuously stirred PBR of 3 L under sinusoidal light with a maximum intensity of 100 μmol/m2.s and light/dark photoperiod of 16:8 h and controlled temperature conditions (18 – 23°C) in modified-WC medium; (ii) Dec 19_Dig was grown at the same conditions as (i) but with 10% (v/v) paper-filtered liquid fraction of digestate as a nutrient source; (iii) Aug 20_Dig was grown in a pilot-scale tubular PBR of 550L with 2.5% (v/v) paper-filtered liquid fraction of digestate as a nutrient source and an average temperature in the greenhouse during cultivation of 26°C

During the control experiments in NO3- dominant modified-WC medium, Palmitic acid (C16:0), Oleic acid (C18:1n-9cis), Linolenic acid (C18:3n-3) alpha and Linolelaidic acid (C18:2n) were present in the highest levels within the biomass dominated by Chlorella and Desmodesmus cultures. These values were much higher than that observed in the digestate grown biomasses at lab- and pilot-scale. However, within digestate-grown samples, the fatty acid composition was observed to be correlated to the scale of operation with Linolenic acid alpha being much higher under lab-scale conditions and Palmitic acid independent of the scale of operation. For medium concentrated fatty acids, similar levels were observed among all the biomasses specifically Stearic acid (C18:0), Lauric acid (C12:0), Palmitoleic acid (C16:1) with exception of Heptadeconic acid (C17:0) which is much higher in the control experiment. On the other hand, a dependence of the seasonality, scale of operation and, source and speciation of N was observed for the remainder of the fatty acid composition but these were present in low to negligible levels.

Elemental Composition

The elemental composition of the biomass expressed in terms of carbon content followed a similar pattern as the carbohydrates plus lipids content. However, the total N contents found were similar across the different conditions, while the protein content was different. As a common practice, the elemental %N, when multiplied by 6.25, provides an estimate of the protein content (direct relation).

However, the observation in this study indicates that there was no such direct correlation established between the protein content and % N values. Such a calculation could generate an unreliable value, as it is not specific for microalgae and has been shown to vary depending on the species, culture composition, amino acid profiling and cultivation conditions.

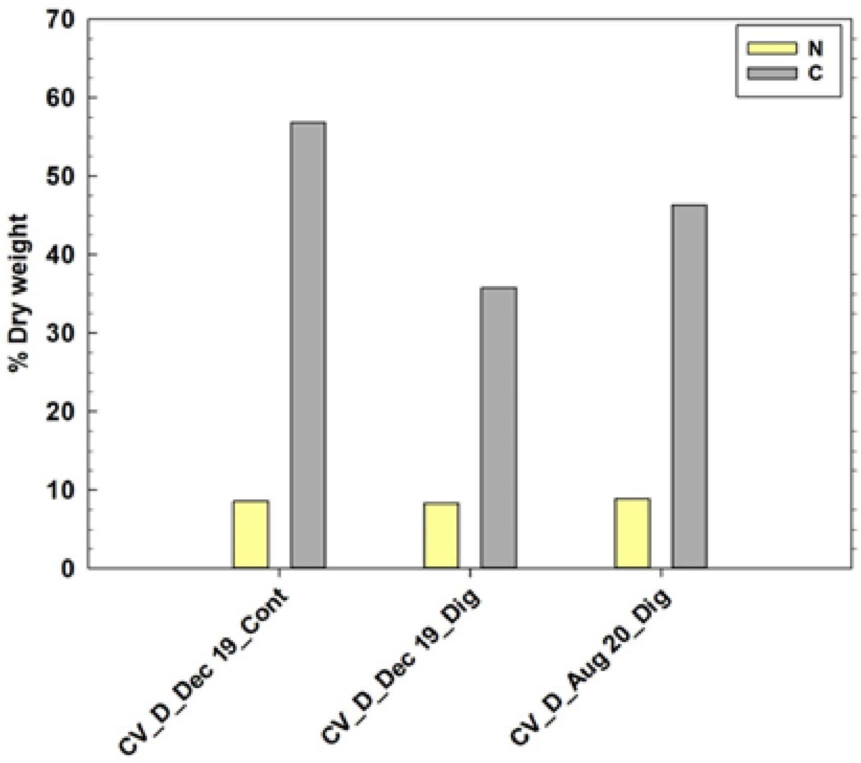

Figure 10 Total C and total N contents of the microalgae biomass produced from different batches: (i) Dec 19_Cont was grown in a lab-scale continuously stirred PBR of 3 L under sinusoidal light with a maximum intensity of 100 µmol/m2.s and light/dark photoperiod of 16:8 h and controlled temperature conditions (18 – 23°C) in modified-WC medium; (ii) Dec 19_Dig was grown at the same conditions as (i) but with 10% (v/v) paper-filtered liquid fraction of digestate as a nutrient source; (iii) Aug 20_Dig was grown in a pilot-scale tubular PBR of 550L with 2.5% (v/v) paper-filtered liquid fraction of digestate as a nutrient source and an average temperature in the greenhouse during cultivation of 26°C

COOPERL, Brittany (FR) - Algal biomass analysis

Pilot scale culture and sampling

The digestate used to grow A. mangrovei was the result of the anaerobic digestion of pig manure at the pilot site (Cooperl, Lamballe). Prior microalgal cultivation, the digestate was treated using ultra-filtration at a pore size of 300 kDa. The composition of the raw digestate can be found above. Two 800 L poly(methylmethacrylate) (PMMA)made cylinders were used to produce large amount of biomass in non-axenic conditions. Water (for process and for cleaning) was supplied by a pump and delivered at top of the cylinders through a rotating nozzle. Agitation and O2 supply in each cylinder were provided by airflow bubbling from the bottom of the cylinder, at a rate of 0.4 volume of air per volume of culture per minute (Air-lift system). -

The medium for the batch culture was composed of industrial scale glucose syrup (final concentration : 24 g/L), sterilised YEP medium (final concentrations : 2 g/L Tryptone; 2 g/L Yeast Extrac), digestate previously filtrated as described above (final concentrations 2,5 %), and sea salt (final concentration 15 g/L). The two 800 L cylinders were filled with 500 L of the pilot scale medium and inoculated with 8L axenic cultures. Temperature of the culture was regulated between 28 and 30°C while pH was maintained above 4.5 by regular addition of 10N NaOH solution. Samples for the cell counts and biochemical analysis were regularly collected during culture growth for 17 batches and results are presented below.

Cell counts and protein contents

A

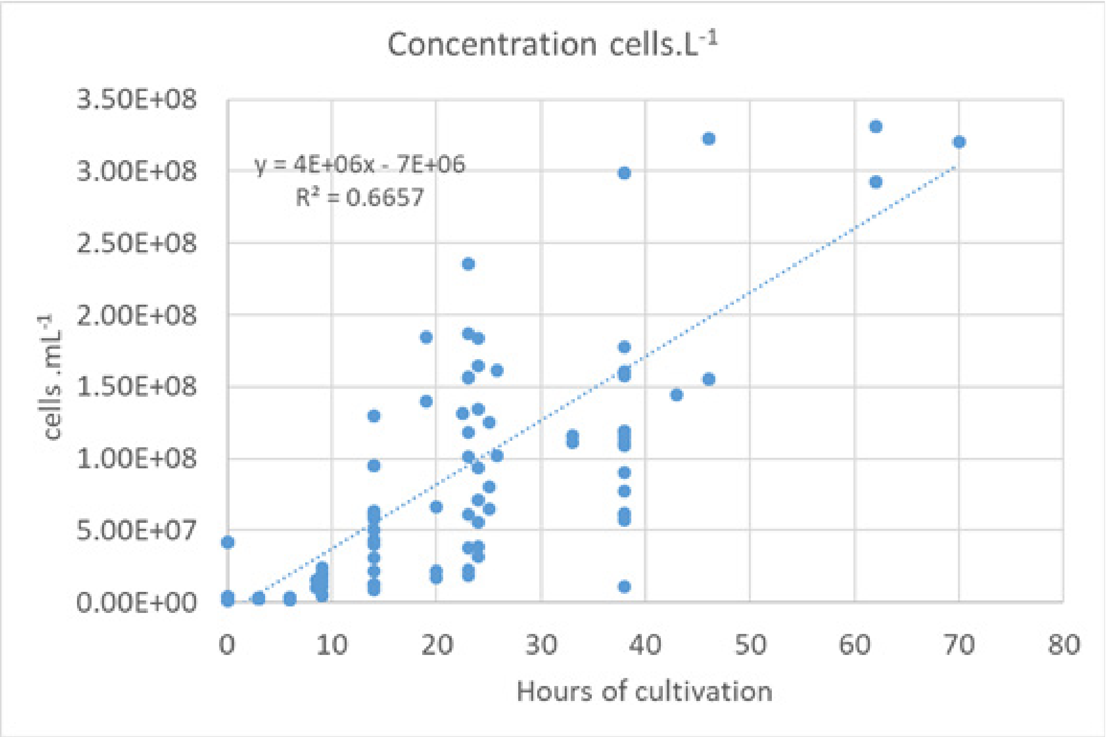

Figure 11 A Cell concentration in cell.mL-1 (A) obtained during cultivation of Auranthiochytrium mangrovei with 2.5% digestate at Cooperl from June 2020 till January 2021

B

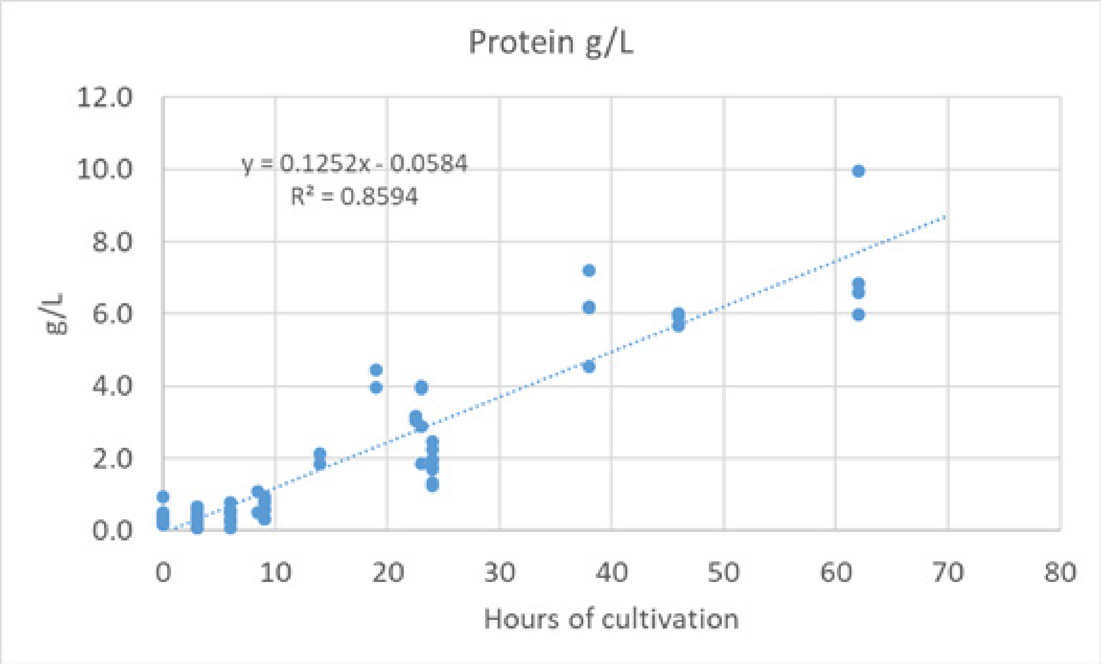

Figure 11 B Protein content in g.L-1 (B) obtained during cultivation of Aueanthiochytrium mangrovei with 2.5% digestate at Cooperl from June 2020 till January 2021

Overall, cell concentration (cell.L-1) and protein content (g.L-1) increased with the duration of culture. The percentage of protein per DW is 57% on average.

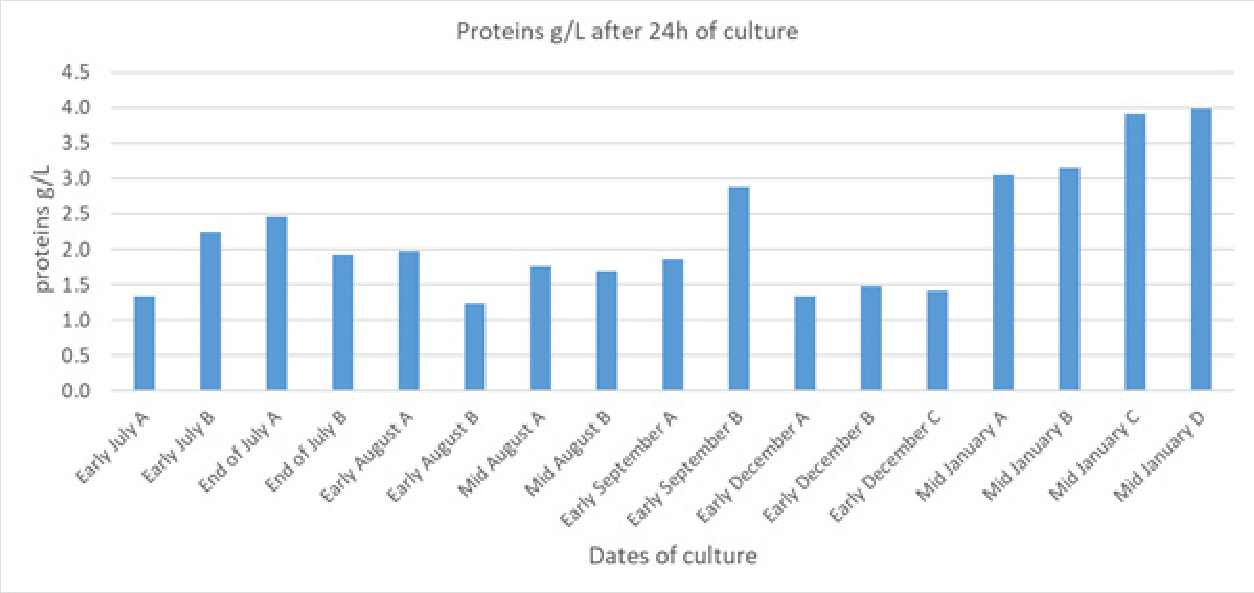

Figure 12 Cell : Seasonal variation of protein content after 20-24 hours of cultivation of Aurantiochytrium mangrovei with 2.5% digestat at Cooperl from June 2020 till January 2021.

When compared at one sampling time (20-24h), the protein content varied from 1.2 g/L to 4.0 g/L. However, these variations from batch to batch cannot be associated clearly to the . seasonal pattern. The highest values in January 2021 can be attributed to improved cultivation practices (temperature regulation, better inoculum etc.). .

Lipid and docosahexaneonic acid (DHA) contents

A

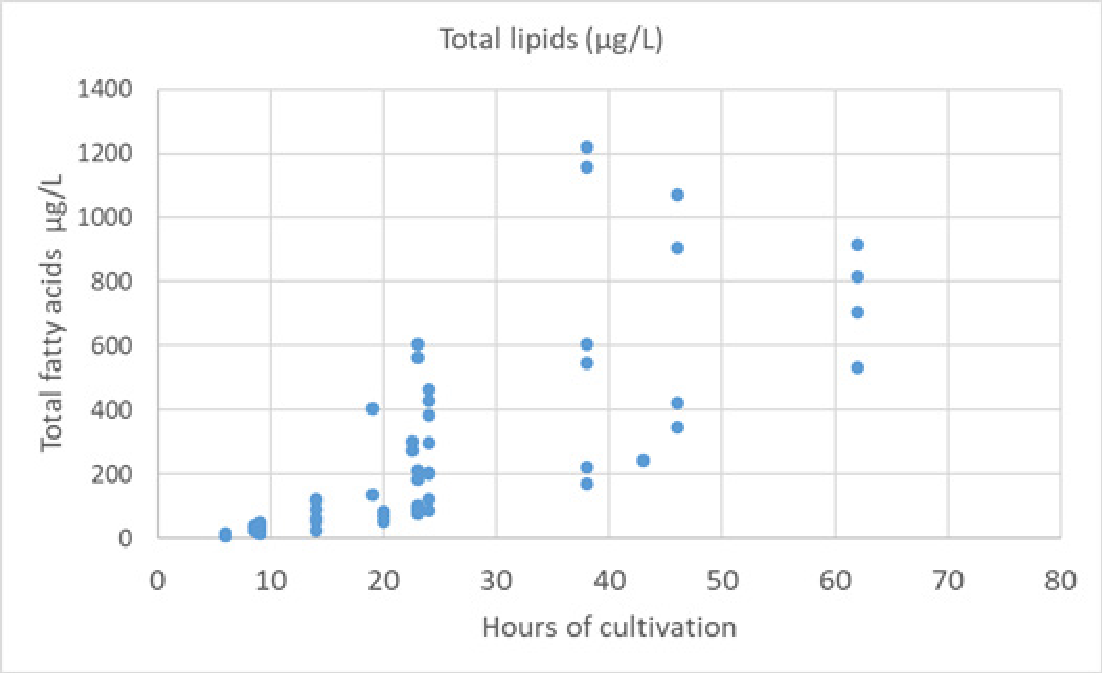

B

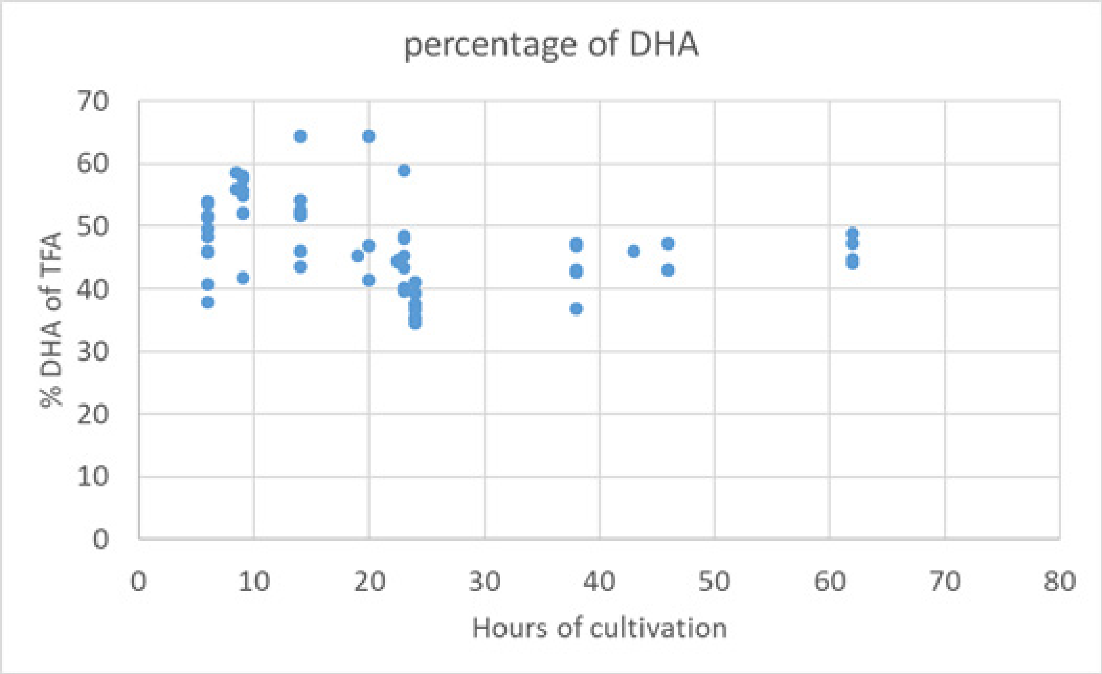

Figure 13 Total fatty acids (TFA) content (A) and percentage of DHA (% TFA) (B) obtained during cultivation of Auranthiochytrium mangrovei with 2.5% digestate at Cooperl from June 2020 till January 2021.

As for the protein content, total fatty acid content increased with duration of culture while the percentage of DHA (targeted valuable compound) remained fairly stagnant during . the culture and according to batches (see below for detailed fatty acid profiles). The mean percentage of total fatty acid per DW is 6%.

A

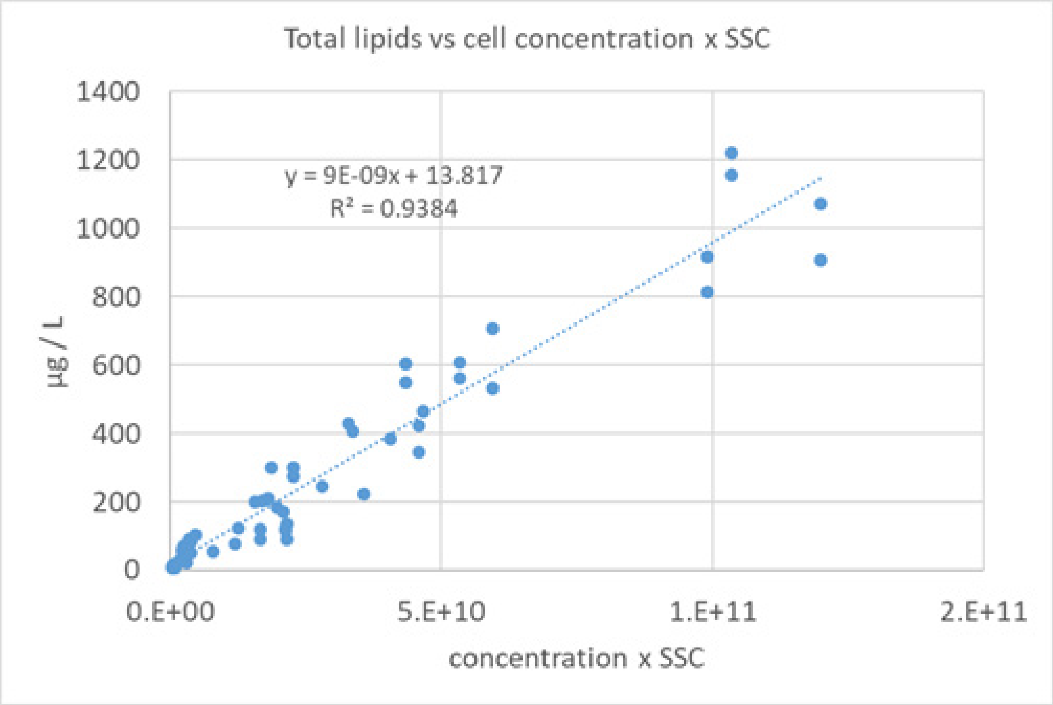

B

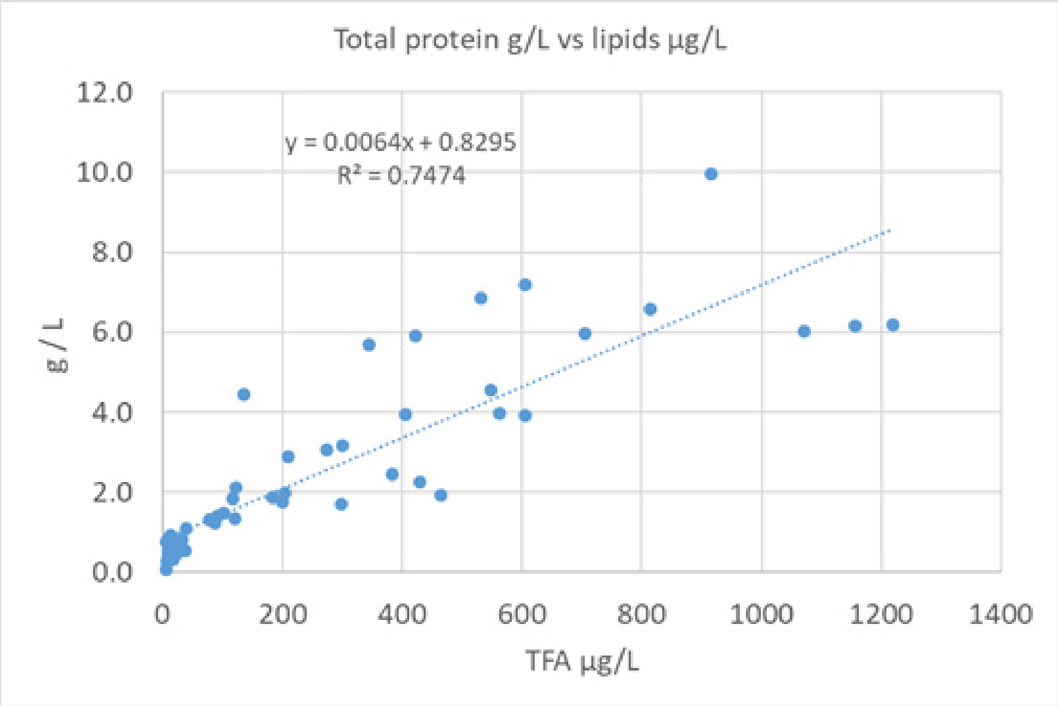

Figure 14 Relationships between total lipids and concentration (cell.L-1) x SSC (side scatter value) obtained flow cytometry and between total lipid content (µg/L) and protein content (g/L) of Auranthiochytrium mangrovei cultivated with 2.5% digestate at Cooperl from June 2020 till January 2021.

We observed a very interesting relationship between total fatty acid expressed in µg/L and the cell concentration multiplied by the side scatter (cell complexity) both measured flow cytometry during culture monitoring. Cell concentration x SSC provided a good estimate of the lipid production by the culture.

This proxy was measured by flow cytometry in few minutes on live without any preparation. It may help to better master the batch-to-batch variability (see below) of lipid production we observed during the course of biomass production for feed trials.

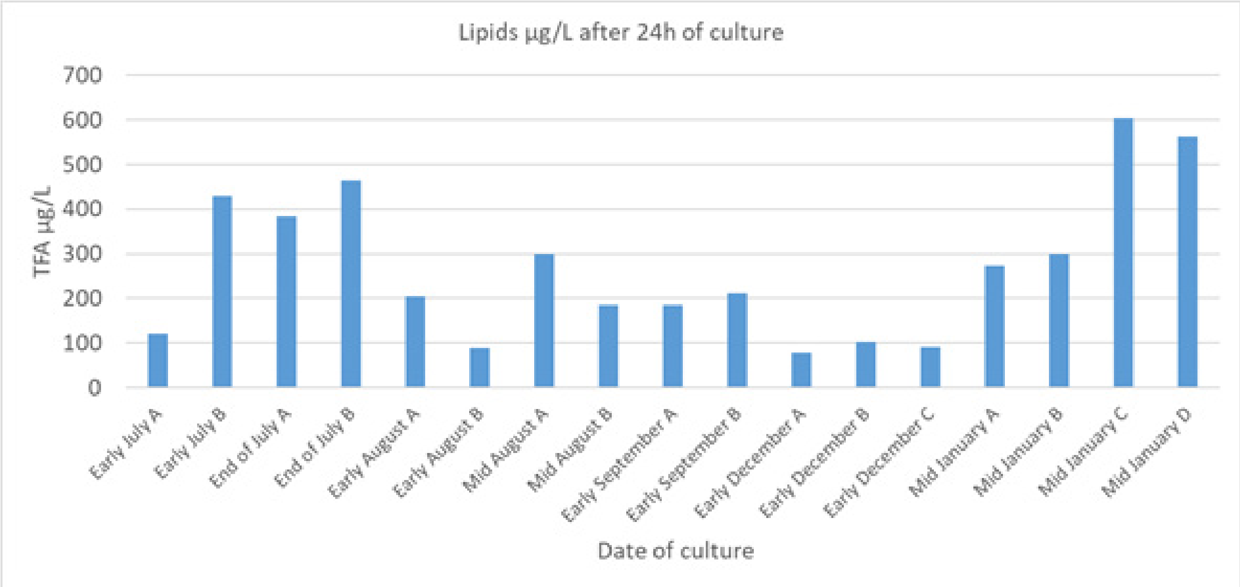

Figure 15 Seasonal variation of total lipid content after 20-24 hours of cultivation of Auranthiochytrium mangrovei with 2.5% digestate at Cooperl from June 2020 till January 2021.

When compared at one sampling time (20-24h), lipid content varied from 0.1 g/L to 0.6 g/L. There is no clear seasonal pattern for lipid content as highest values were obtained in summer and in winter. We may further improve cultivation practices by monitoring in real time by flow cytometry the cell concentration x SSC proxy. For example, lipid synthesis rely on supplied sugar to the culture, such real time proxy may allow adjusting sugar supply during the course of the culture.

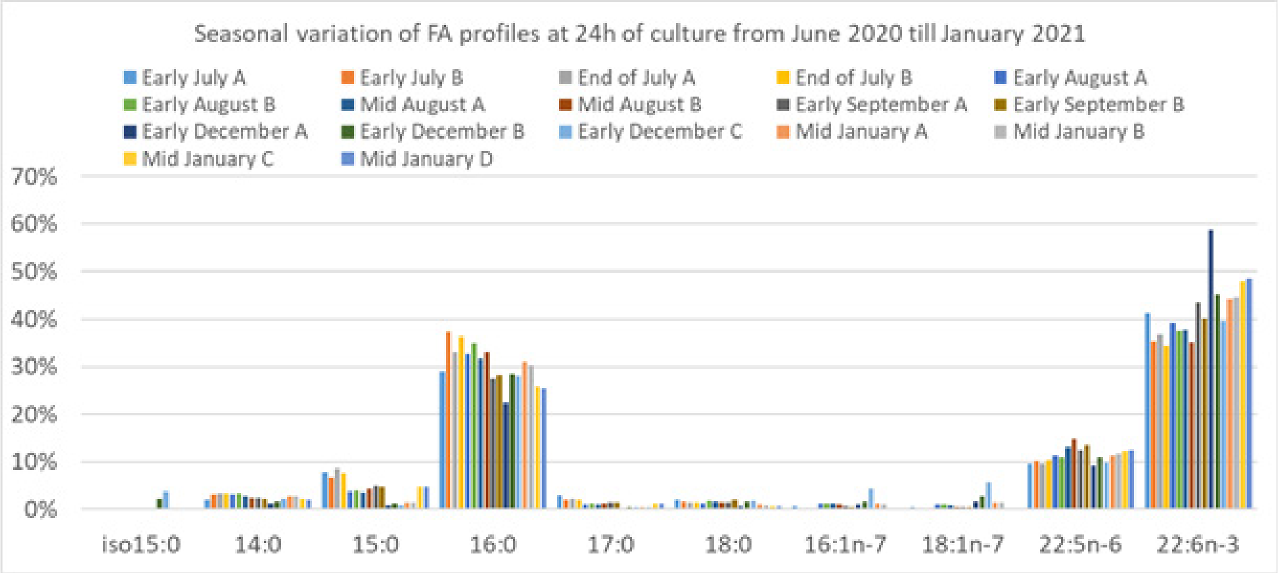

Figure 16 Seasonal variation of fatty acid profiles of Auranthiochytrium mangrovei after 20-24 hours of cultivation with 2.5% digestate at Cooperl from June 2020 till January 2021.

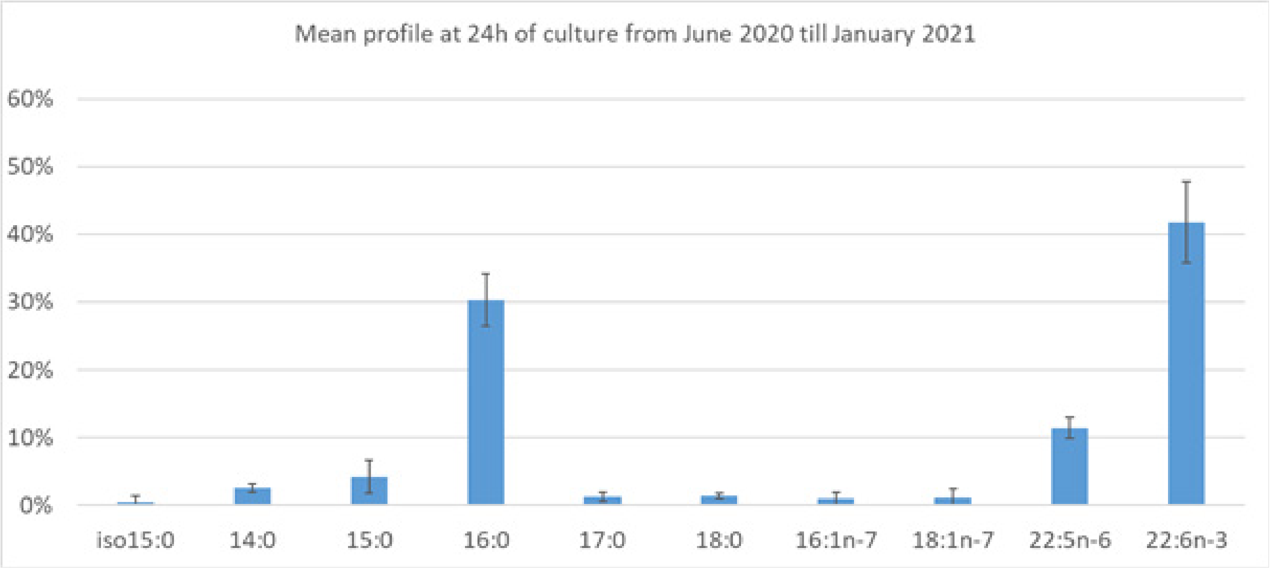

Figure 17 Calculated mean from June 2020 till January 2021 of the fatty acid profile of Auranthiochytrium mangrovei after 20-24 hours of cultivation with 2.5% digestate at Cooperl

The fatty acid profile is dominated by three fatty acids; 22:6n-3, 16:0 and 22:5n-6. The targeted compound (22:6n-3 also called DHA) varied between 35% to 58% with no obvious seasonal pattern. The DHA average production is 0.112 g/L/24h.

3 CONCLUSIONS AND RECOMMENDATIONS

In case of the Langage AD, Devon (UK) pilot, as only two batches of digestate were used during this period of Scenedesmus sp. biomass production, we can not draw any conclusion regarding seasonal variation on digestate quality. Compared to commercial medium, cultivating Scenedesmus sp. on digestate (~2.5%) improved the overall biochemical composition, such as the total protein (from 52% to 72%), fatty acids (only in winter, from 7.36% to 8.83%) and pigments (carotenoids from 0.13% to 0.45% and chlorophylls from 1.86% to 2.55%). Recovery of carbohydrates showed negligible variations. In contrast, fatty acids recovery in summer season (SQ_Jan_20_Dig) was decreased slightly. The increase in protein content is due to the easy assmiliation of nitrogen form (ammonium in case of digestate as opposed to nitrate in the commercial medium). The results presented here highlights the potential of Scenedesmus sp. in bioremediation of excess nutrients in digestate (ammonium form) and producing microalgae biomass with improved macromolecular composition for animal feed and other bio-based applications – validating the circular economy concept. Furthermore, in terms of biochemical composition of Scenedesmus sp. biomass grown on digestate (~2.5%) at different seasons (winter (Jan_20) and summer (Jul_20), no significant variation was noted with respect to recovery of proteins, carbohydrates, key individual fatty acids and pigments. As expected, in case of total fatty acid content, recovery was higher in winter compared to summer as cells need less energy reserve during summer, using mainly carbohydrates energy for the different metabolic requirements. Overall, we found no significant influence of seasonal variations on the quality of Scenedesmus sp. biomass recovered. Ensuring validation of such circular economy concept from a seasonal variation perspective will allow sustainable supply of nutrient-rich biomass throughout the year for the animal feed purposes.

In case of the UGhent-Innolab, Flanders (BE) pilot, working with a consortium of Desmodesmus sp. and Chlorella vulgaris, it seems that the change of growing conditions (i.e., medium used and seasonal variance) mainly affected the ratio of Desmodesmus to Chlorella cells. This in turn alters the cellular composition, with an enhanced effect on the lipid content and composition. The other differences found could be mainly related to starch accumulation due to N depletion rather than the seasonal changes. Therefore, if an algal facility decides to use a consortium to increase the robustness of their operation over long-term, as done at the UGhent-Innolab pilot facility, the produced biomass is bound to suffer from composition fluctuations due to population shift induced by medium or weather changes. Such facilities should opt for a versatile end-applications that are not significantly affected by these compositional changes.

In case of the Cooperl, Brittany (FR) pilot, the Auranthiochytrium mangrovei biomass was cultured and harvested from July 2020 till January 2021 and analysed for protein, lipid, fatty acid and elemental composition. As only two batches of digestate were used during this period of biomass production, we can not draw any conclusion regarding seasonal variation on digestate quality. Indeed, individual batch variability of protein and lipid contents (from 1.2 g/L to 4.0 g/L for proteins and from 1.2 g/L to 4.0 g/L for lipids) likely masked potential seasonal variation of biomass composition and production yield. The overall increase of protein content through the year most likely reflect improvement in managing culture conditions (Silkina A. et al., 2020). Nevertheless, the fatty acid profiles were fairly stable from batch to batch. Furthermore the targeted compound (22:6n-3, DHA) for animal feed averaged at 40% of total fatty acid, even tending to increase toward the end of batch production period.

REFERENCES

Craggs, R. J., Lundquist, T. J., & Benemann, J. R. (2013). Wastewater Treatment and Algal Biofuel Production. Algae for Biofuels and Energy, 153–163. https://doi.org/10.1007/978-94-007-5479-9_9

de Souza, M. F., Rodrigues, M. A., Bon, E. P. da S., & Freitas, S. P. (2019). Interference of starch accumulation in microalgal cell growth measurement. Journal of Applied Phycology, 31(1), 249–254. https://doi.org/10.1007/S10811-018-1566-3/FIGURES/4

Fathi, A. A., Azooz, M. M., & Al-Fredan, M. A. (2013). Phycoremediation and the potential of sustainable algal biofuel production using wastewater. American Journal of Applied Sciences, 10(2), 189–194. https://doi.org/10.3844/AJASSP.2013.189.194

Fernandes, F., Aouamri, N., Fernandes de Souza M., Fuentes-Grünewald, C., Jones, G., Seelam J.S., Sigurnjak I., & Llewellyn, C. (2019). Best Practices for the treatment and preparation of nutrient-rich digestate for algal cultivation. Public Output report of the ALG-AD project. www.nweurope.eu/projects/ALG-AD.

Fernandes, F., Silkina, A., Fuentes-Grünewald, C., Wood, E. E., Ndovela, V. L. S., Oatley-Radcliffe, D. L., Lovitt, R. W., & Llewellyn, C. A. (2020). Valorising nutrient-rich digestate: Dilution, settlement and membrane filtration processing for optimisation as a waste-based media for microalgal cultivation. Waste Management, 118, 197–208. https://doi.org/10.1016/J.WASMAN.2020.08.037

Fuentes-Grünewald, C., Ignacio Gayo-Peláez, J., Ndovela, V., Wood, E., Vijay Kapoore, R., & Anne Llewellyn, C. (2021). Towards a circular economy: A novel microalgal two-step growth approach to treat excess nutrients from digestate and to produce biomass for animal feed. Bioresource Technology, 320, 124349. https://doi.org/10.1016/J.BIORTECH.2020.124349

Guillard, R. R. L., & Lorenzen, C. J. (1972). YELLOW-GREEN ALGAE WITH CHLOROPHYLLIDE C1,2. Journal of Phycology, 8(1), 10–14. https://doi.org/10.1111/J.1529-8817.1972.TB03995.X

Judd, S., van den Broeke, L. J. P., Shurair, M., Kuti, Y., & Znad, H. (2015). Algal remediation of CO2 and nutrient discharges: A review. Water Research, 87, 356–366. https://doi.org/10.1016/J.WATRES.2015.08.021

Luo, S., Berges, J. A., He, Z., & Young, E. B. (2017). Algal-microbial community collaboration for energy recovery and nutrient remediation from wastewater in integrated photobioelectrochemical systems. Algal Research, 24, 527–539. https://doi.org/10.1016/J.ALGAL.2016.10.006

Olguín, E. J. (2012). Dual purpose microalgae–bacteria-based systems that treat wastewater and produce biodiesel and chemical products within a Biorefinery. Biotechnology Advances, 30(5), 1031–1046. https://doi.org/10.1016/J.BIOTECHADV.2012.05.001

Papadimitriou, E. K., Barton, J. R., & Stentiford, E. I. (2008). Sources and levels of potentially toxic elements in the biodegradable fraction of autoclaved non-segregated household waste and its compost/ digestate. Waste Management and Research, 26(5), 419–430. https://doi.org/10.1177/0734242X08088697

Silkina A., Fernandes F., Fuentes-Grünewald C., Llewellyn C.A., Ndovela V., Gayo Peláez J.I., de la Broise D., Soudant P., Chauchat L., Seelam J.S., & Fernandes de Souza M. (2020). Best practices for microalgal production using nutrient-rich digestate as a waste-based medium. Public Output report of the ALG-AD project. .

Silkina, A., Ginnever, N. E., Fernandes, F., & Fuentes-Grünewald, C. (2019). Large-Scale Waste Bio-Remediation Using Microalgae Cultivation as a Platform. Energies 2019, Vol. 12, Page 2772, 12(14), 2772. https://doi.org/10.3390/EN12142772

Stiles, W. A. V., Styles, D., Chapman, S. P., Esteves, S., Bywater, A., Melville, L., Silkina, A., Lupatsch, I., Fuentes Grünewald, C., Lovitt, R., Chaloner, T., Bull, A., Morris, C., & Llewellyn, C. A. (2018). Using microalgae in the circular economy to valorise anaerobic digestate: challenges and opportunities. Bioresource Technology, 267, 732–742. https://doi.org/10.1016/J.BIORTECH.2018.07.100

Tambone, F., Adani, F., Gigliotti, G., Volpe, D., Fabbri, C., & Provenzano, M. R. (2013). Organic matter characterization during the anaerobic digestion of different biomasses by means of CPMAS 13C NMR spectroscopy. Biomass and Bioenergy, 48, 111–120. https://doi.org/10.1016/J.BIOMBIOE.2012.11.006
