# Supplementary material for: Scale-Up to Pilot of a Non-Axenic Culture of Thraustochytrids Using Digestate from Methanization as Nitrogen Source
Source: Mar Drugs. 2022 Aug 2;20(8):499. doi: 10.3390/md20080499 (PMC9410245; doi:10.3390/md20080499)
Supplement: Supplementary file 1 [file marinedrugs-20-00499-s001.zip › marinedrugs-1809880-supplementary.pdf]

Scale-up to pilot of a non-axenic culture of Thraustochytrids using digestate from methanisation as nitrogen source.

Denis de la Broise[1]*, Luc Chauchat[1], Mariana Ventura[1], Maurean Guerreiro[1], Teo Michez[1], Thibaud Vinet[2], Nicolas Gautron[1], Fabienne Le Grand[1], Antoine Bideau[1], Nelly Le Goïc[1], Adeline Bidault[1], Christophe Lambert[1] and Philippe Soudant[1]*

[1] Univ Brest, CNRS, IRD, Ifremer, UMR6539 LEMAR, F-29280, Plouzané, France.
[2] DENITRAL, Lamballe, France
* Correspondence: philippe.soudant@univ-brest.fr & denis.de-labroise@univ-brest.fr

Supplementary files

Table S1: Chemical characterisation of digestate from COOPERL organic biological waste (pig manure) in Lamballe (Brittany, France)

Compound/Unit	Values
Digestate Origin	
Dry Matter (%)	0.56
Dry organic matter (% DM)	28.36
Acetic acid (mg/kg)	< 35
Propionic acid (mg/kg)	< 16
Isobutyric acid (mg/kg)	< 9
Butyric acid (mg/kg)	< 7
Isovaleric acid (mg/kg)	< 11
Valeric acid (mg/kg)	< 12
Caproic acid (mg/kg)	< 24
pH	8.21
Conductivity (mS/cm)	17.92
Elemental analysis (mg/l)	B 0.51; As <0.05; Ca 47.5; Cd < 0.005; Co 0.018; Cr < 0.025; Cu < 0.050; Fe 2.16; Hg < 0.005; K 1600; Mg 8.7; Mn 0.050; Mo 0.012; Na 690; Ni 0.072; P 34.4; Pb < 0.050; S 22.8; Se < 0.005; Si 27.4; Sn < 0.050; Zn 0.250 and Cl 875
Ammoniacal nitrogen (g N/kg)	1.93
Total nitrogen (kg/1000kg FM)	1.98
Phosphate (mg/l)	103
Salinity (g/kg)	2.96

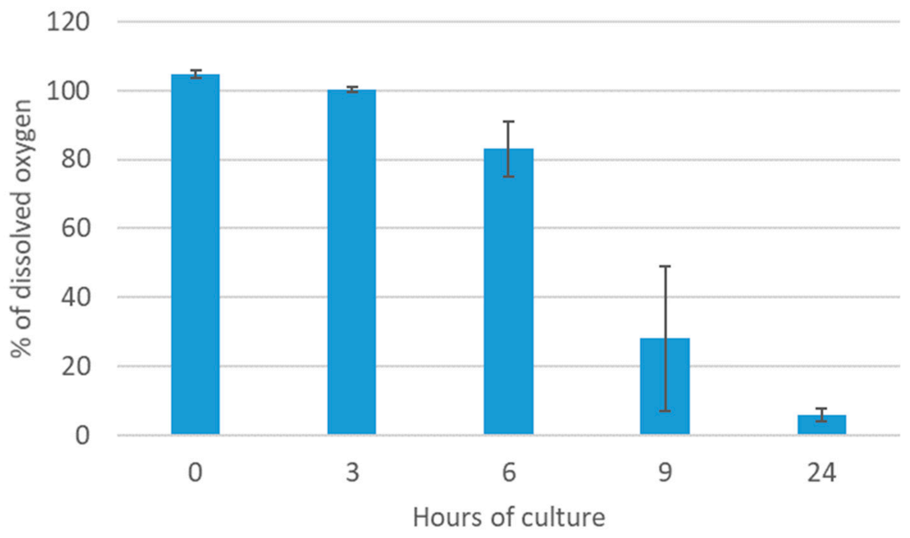

Figure S1: Percentages of dissolved oxygen of *A. mangrovei* cultivated in 800 L cylinder at 0, 3, 6 9, and 24 h. (n=11 batches, Error bars= S.D.).

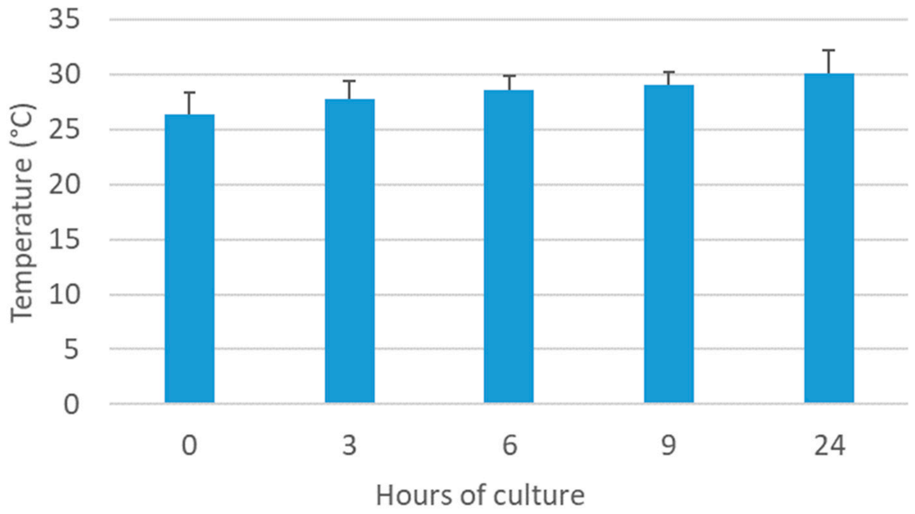

Figure S2: Mean values of *A. mangrovei* culture temperature in 800 L cylinder at 0, 3, 6, 9 and 24 h. (n=11 batches, Error bars= S.D.).

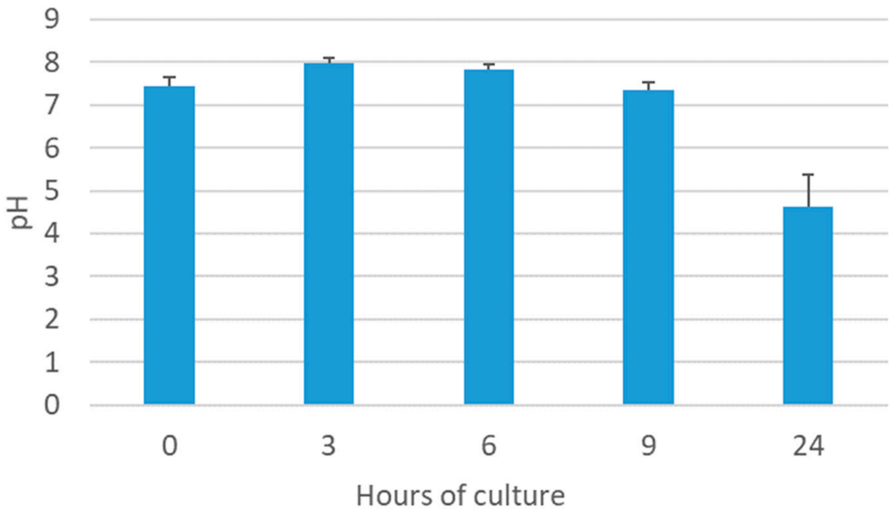

Figure S3: Mean values of *A. mangrovei* culture pH in 800 L cylinder at 0, 3, 6, 9 and 24 h. (n=11 batches, Error bars= S.D.).
